# Contrasting Microbial Taxonomic and Functional Colonisation Patterns in Wild Populations of the Pan‐Palaeotropical C4 Grass, *Themeda triandra*

**DOI:** 10.1111/pce.70205

**Published:** 2025-09-26

**Authors:** Riley J. Hodgson, Christian Cando‐Dumancela, Tarryn Davies, Elizabeth A. Dinsdale, Michael P. Doane, Robert A. Edwards, Craig Liddicoat, Shawn D. Peddle, Sunita A. Ramesh, Jake M. Robinson, Martin F. Breed

**Affiliations:** ^1^ College of Science and Engineering Flinders University Bedford Park South Australia Australia; ^2^ Flinders Accelerator for Microbiome Exploration Flinders University Bedford Park South Australia Australia

**Keywords:** aridity, endosphere, microbial ecology, plant–soil (below‐ground) interactions, rhizosphere, shotgun metagenomics, *Themeda triandra*, two‐step selection process

## Abstract

The interactions between native plants and soil microbiota are not well characterised, despite growing recognition of their importance for host plant fitness and ecological functioning. We used shotgun metagenomics to examine microbial taxonomic and functional colonisation patterns in wild populations of the pan‐palaeotropical C4 grass, *Themeda triandra*, across a globally representative aridity gradient (aridity index 0.318–0.903). We investigated these patterns through the two‐step selection process whereby microbes are recruited from bulk soils into rhizospheres (soil on the root surface), and root interiors (endospheres). We provide clear evidence of this process through decreasing microbial taxonomic diversity from bulk soil to *T. triandra* roots. Surprisingly, microbial functional potential showed the opposite trend: the diversity of potential functions (exponent of Shannon's diversity) increased from bulk soil to the rhizosphere and endosphere, but functional richness did not. Finally, we found that increasing aridity was associated with rhizospheres that were more compositionally similar, yet remained highly diverse in functional potential. Overall, aridity is strongly associated with the root‐associated microbiome of *T. triandra*, selecting for microbiota that likely support plant resilience under dry conditions. Furthermore, microbial functional potential closely tracks taxonomic composition and aridity trends, highlighting how native plants can shape their microbial communities.

## Summary statement

Our study shows that microbial functional diversity diverges from taxonomic patterns, revealing hidden complexity in plant–microbe interactions and advancing understanding of how microbes influence plant responses to environmental conditions.

## 1 Introduction

Soils are home to 59% of all species (Anthony et al. 2023), including many microbes that form belowground associations with plants. These microbiota have important functional roles, such as supporting the fitness of plants through mutualistic plant‐microbe interactions (Bever 1994). Host plants invest resources that sustain and prioritise specific microbial functions. In return, mutualistic microbes influence plant metabolic processes, provide fitness advantages to their hosts, aid plant resource acquisition and increase plant tolerance to environmental stressors (Petipas et al. 2021). Beneficial interactions between plants and soil microbiota can also shift under changing environmental conditions, with increasing aridity thought to intensify plant reliance on mutualistic microbes as conditions become more stressful (Jiang et al. 2024). However, the processes that control how these functionally important microbiota colonise, or are recruited into plant roots, are complex and poorly understood in natural ecosystems, especially where plants must navigate variable and changing biotic and abiotic conditions (Bulgarelli et al. 2013).

The two‐step selection process is a well‐established theoretical framework to view the colonisation of roots by microbes but is poorly studied outside of model species such as *Arabidopsis* (Bulgarelli et al. 2012), *Setaria viridis or Setaria pumila* (Escobar Rodríguez et al. 2018) and crop species such as tomatoes, rice, maize and soybean (Cavaglieri et al. 2009; Richter‐Heitmann et al. 2016; Barajas et al. 2020; Xun et al. 2024). This process includes the active recruitment of microbes by host plants, first from bulk soils into plant rhizospheres (soil on the surface of roots) and then from rhizospheres into roots (the endosphere) (Bulgarelli et al. 2013; Lundberg et al. 2012). In *Arabidopsis*, for instance, bacterial and fungal communities are well known to shift among the bulk soils, rhizospheres and endospheres, with a progressive decline in taxonomic alpha diversity as microbiota are filtered into the endosphere (Bulgarelli et al. 2012). Alternative theoretical frameworks include vertical transmissions of microbiota into plant seeds directly from parental plant flowers or via pollinators (Abdelfattah et al. 2023), or internal transport through plant vascular tissue from leaves (phyllosphere microbiota) (Chi et al. 2005). Despite their importance, functional investigations of these colonisation patterns—especially in non‐model plant species—remain scarce, limiting our understanding of host‐microbe dynamics under natural conditions. It also remains uncertain how environmental change impacts this two‐step selection process in plants under natural conditions.

The C4 grass *Themeda triandra* (Forssk.), is a keystone species with a pan‐palaeotropical distribution across much of Australia, Asia and Africa, often dominating grassland ecosystems (Snyman et al. 2013). It provides important ecosystem services by maintaining soil health (e.g., shaping physical structure and microbiota) and supporting native biodiversity (Snyman et al. 2013). While this species is known to be colonised and receive growth benefits from soil microbiota (Hodgson et al. 2024; Petipas et al. 2017), the different roles provided by their functional genes are unexplored. In this field study, we used a natural experimental design and shotgun metagenomics to investigate the colonisation patterns of microbiota, and their accompanying gene functions, in wild *T. triandra* across a globally representative aridity gradient. Aridity represents an important global condition that shapes vegetation, productivity and the structure and function of soil microbial communities. Its importance is expected to increase under climate change, as many regions are projected to become drier, especially in southern Australia (Guerin et al. 2018), intensifying water stress on ecosystems and altering plant‐microbe interactions that underpin ecosystem resilience and function (Delgado‐Baquerizo et al. 2013; Maestre et al. 2015). As such, understanding plant‐microbial dynamics will not only reveal the selective processes that shape root‐associated microbial functions in native grasslands but also clarify the role of these functions in supporting plant persistence in ecologically important regions under future climate scenarios.

We hypothesised that (1) the microbial taxonomic and functional colonisation patterns in *T. triandra* would align with the two‐step selection process (i.e., community and diversity filtering from bulk soil into roots); (2) there will be strong positive correlations between microbial taxonomic and functional colonisation patterns, where we expect that higher bacterial species diversity will be associated with higher functional diversity (irrespective of the two‐step selection process); and, (3) aridity will modulate both taxonomic and functional colonisation patterns, with higher aridity populations recruiting microbiota linked to water stress tolerance and drought resilience.

## 2 Methods

### 2.1 Study Design

**Figure 1:**
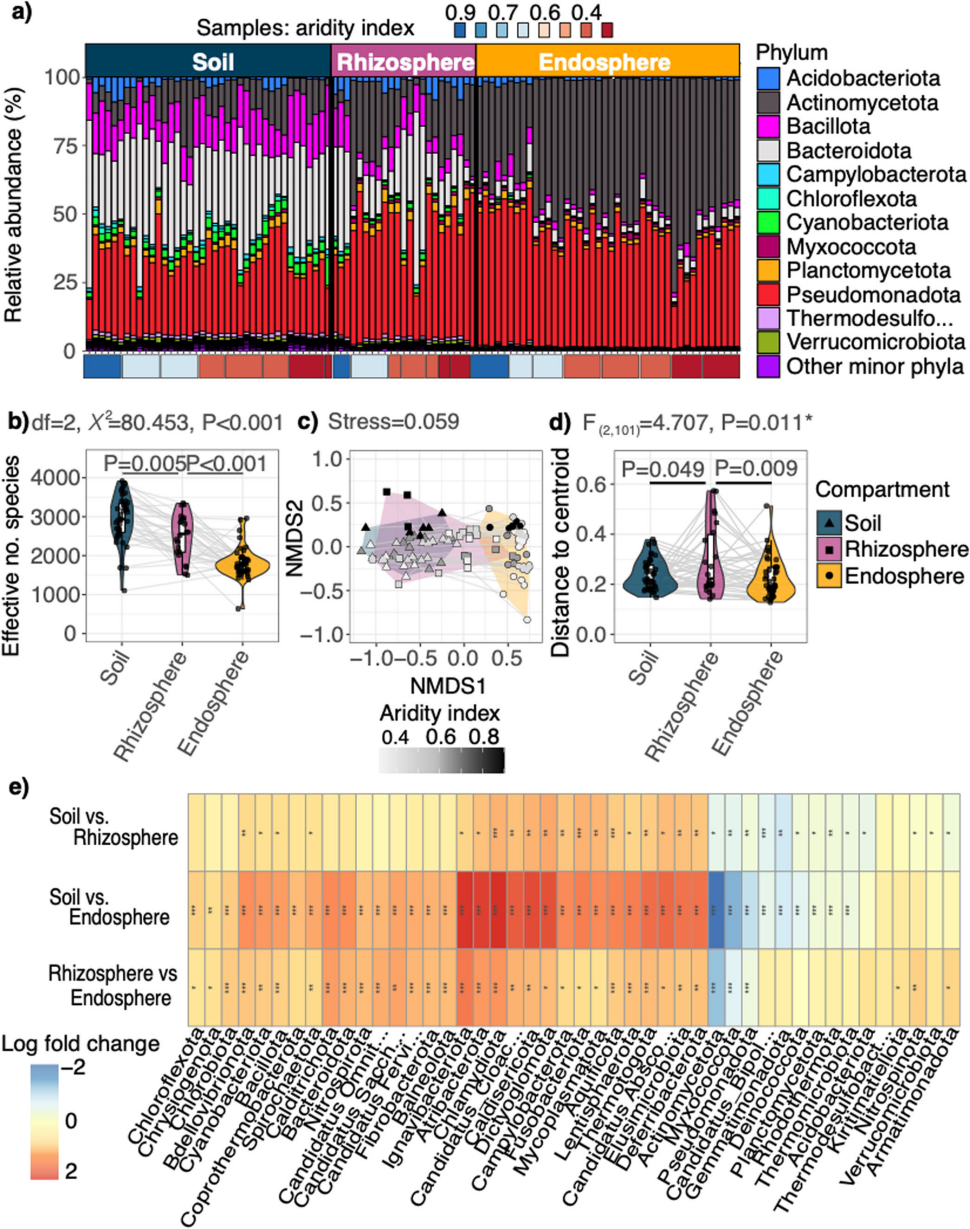
Alpha and beta diversity metrics for normalised bacterial species abundances associated with plant compartments (i.e., bulk soil, rhizosphere, endosphere). (a) Relative abundances of bacterial phyla across all bulk soil, rhizosphere and endosphere samples. Sample labels are ordered by aridity index and box colours denote plant compartment. (b) Alpha diversity estimates for bacteria associated within their plant compartments for the effective number of species (see Table S7 for statistical output). (c) Bacterial community compositions with plant compartments are shown via NMDS ordinations with Bray–Curtis distances (stress = 0.059; Table S8) (beta diversity). Point shape and hull colours represent samples from each different plant compartment, and point colour shows the aridity index. (d) Beta dispersion differences are represented by distance to centroid estimates for each compartment (see Table S9). (e) Heatmap showing all differentially abundant bacterial phyla across bulk soils, rhizosphere and endosphere at the 0.05 significance level based on log fold change (see Table S13 for details, including full names of abbreviated phyla). Log fold changes are reported as Group 1 vs. Group 2, where Group 2 is the reference category (i.e., positive values indicate greater abundance in Group 1 relative to Group 2).

We sampled the bulk soils, rhizospheres and endospheres of six randomly selected *T. triandra* plants in eight populations that occurred across a southern Australian aridity gradient (32.66–35.07°S, 137.71–139.10°E; Table S1; Figure S1). Sites had mean annual aridity index values of 0.318–0.903, mean annual precipitation of 392–776 mm and mean annual temperatures of 14.6°C–16.5°C and elevation ranged from 147 to 407 m above sea level (Table S1). Sampling across these compartments utilised a space‐for‐time substitution approach (i.e., where spatially distinct plant compartments can represent sequential stages of microbial transition). Aridity data were collected from the Atlas of Living Australia (Belbin et al. 2021; ALA 2014) via the mean annual aridity index layer (annual precipitation/annual potential evaporation; Figure S1b,c). Using this aridity index, low values correspond to more arid conditions (i.e., typically hotter/drier conditions; hereafter, high aridity) (UNEP 1992; Middleton and Thomas 1997). We used a Mantel test to assess whether our sampling design was influenced by spatial autocorrelation, by comparing pairwise aridity and geographic distances. The non‐significant result (*p* = 0.489, *r* = –0.021; Figure S1c) indicates no evidence of spatial autocorrelation, that is, geographically closer sites did not tend to have more similar aridity levels. Furthermore, based on the known global distribution of *T. triandra* (GBIF.org 2023), we estimate that the aridity gradient sampled in this study encompasses the natural distribution of approximately 41%–87% of all global occurrence data sourced from the Atlas of Living Australia (Belbin et al. 2021; ALA 2014) and version 3 of the Global Aridity Index and Potential Evapotranspiration Database (Global‐AI_PET; Zomer et al. 2022; Figure 1c), respectively (Figure S1). In this study, we do not experimentally distinguish between plant‐driven microbial shifts across compartments (i.e., active recruitment of microbiota) and microbial‐dependent changes (i.e., colonisation).

### 2.2 DNA Extraction, Amplification and Sequencing

We collected bulk soils within 30 cm of the north and south of each *T. triandra* plant, and combined these samples for downstream analysis, transporting them on ice from the field and storing them at −20°C for later DNA extraction (Figure S1e). Microbiota from the rhizospheres were isolated following the protocol from Hodgson et al. (2024). Briefly, sampled roots were washed in 0.02% Silwet L‐77 amended PBS buffer and vortexed before being filtered at 100 µm and centrifuged before DNA extraction. We also isolated *T. triandra* endospheres by removing bacteria and DNA from root surfaces, and subsequently extracting DNA directly from these ‘cleaned’ root tissues as detailed in Hodgson et al. (2025). Here, roots were sonicated on ice in 0.02% Silwet L‐77 amended PBS buffer at 30% amplitude for 5 × 30 s alternating burst/rest periods (5 min total) to remove external bacteria and DNA. The roots then underwent a series of five additional washes in this sterilised, amended PBS buffer solution (5 min). To obtain the final endosphere samples, roots were pulverised with metal beads for 1 min in a bead‐beating solution (PowerSoil Kit, Qiagen, Hilden, Germany). Sterilisation was validated by plating final wash solutions on Luria‐Bertani agar plates (Hodgson et al. 2024). Following the manufacturer's protocols, DNA extractions were performed on the soil, rhizosphere and endosphere samples using the DNeasy PowerLyzer PowerSoil Kit (Qiagen, Hilden, Germany).

### 2.3 Shotgun Metagenomic Data Analysis

We performed shotgun metagenomics sequencing on each of the *T. triandra* microbiota samples across our aridity gradient, generating high‐quality sequence data appropriate for fine‐scale taxonomic and functional annotation. We successfully sequenced 43 endospheres, 22 rhizospheres and 39 bulk soils (*n* = 104). Libraries were prepared using Accel‐NGS 2S DNA Library Kits from Swift Biosciences Inc. (London, United Kingdom) and sequenced at the South Australian Genomics Centre (Adelaide, Australia). For sample sequencing, an equimolar pool was prepared and denatured for DNA Nanoball (DNB) generation using the MGI DNBSEQ‐G400 platform with 300 bp reads. All bioinformatics was done using DeepThought high‐performance computing (Flinders University 2021). Data cleaning was done with fastp v0.23.2 (Chen et al. 2018), which included trimming adaptors from DNB sequences. Contaminant DNA from *T. triandra* was removed using reference genomes via Bowtie2 v2.4.1 (Langmead and Salzberg 2012; National Centre for Biotechnology Information NCBI 2021). Taxonomic IDs were assigned using Kraken2 v2.0.7 (Wood et al. 2019). We used Bracken (Lu et al. 2017) to estimate abundances of taxa, then KrakenTools (Lu et al. 2022) to generate a taxonomic abundance table for downstream analysis, using species‐level assignments. Potential gene functions were assigned to reads using SUPER‐FOCUS v1.6 (Silva et al. 2015), according to each functional subsystem from the SEED database (Overbeek et al. 2004), referred to as ‘functions’, hereafter.

### 2.4 Statistics

Statistics were done in R version 4.2.2 (R Core Team 2022). We performed relative abundance normalisations on our taxonomic and functional datasets to account for differences in sequencing depth across our samples. We ran detailed downstream analysis on relative functional gene abundances, but we also isolated six different categories at SEED subsystem level 1 to explore in greater detail (Overbeek et al. 2004). These six functional categories included: motility and chemotaxis (movement and sensing), nitrogen metabolism, phosphorus metabolism, regulation and cell signalling (which captures quorum sensing and biofilm production dynamics), stress response functions and secondary metabolism.

#### 2.4.1 Taxonomic and Functional Diversity Analysis

We calculated the relative abundance of the top bacterial phyla (based on reads classified at the Phylum level) and potential functional categories (at subsystem level 1). Alpha diversity was assessed by computing the effective number of species and functions, using the exponential of Shannon's diversity index (Jost 2006). This transformation enhances interpretability by expressing diversity as the number of equally abundant species or functions that would yield the same Shannon index. We used linear mixed‐effects models to explore how plant compartments (soil, rhizosphere, endosphere), sampling population and aridity index (each included as fixed factors) affected alpha diversity metrics (richness, effective no. of species/functions and Pielou's evenness index). Plant ID was treated as a random factor to account for bias in repeated measures of individual plants across the root/soil compartments.

We used non‐metric multidimensional scaling ordination (NMDS) with Bray–Curtis distances to explore differences in taxonomic and functional community composition. We then tested for differences between group centroids via permutational multivariate analysis of variance and for homogeneity of group dispersions by calculating the distance to centroid measures.

To compare compositional (beta diversity) changes across our aridity gradient, we calculated the average Bray–Curtis distance of each sample to every other sample. We tested the relationship of the aridity index on these distances by calculating the slope of the linear trend between the aridity index and Bray–Curtis distances. Using bootstrapped (*B* = 2000) 95% CIs, we assessed whether the model slope overlapped zero. See Tables S2 and S3 for a more detailed summary of all statistical tests comparing aridity with alpha and beta diversity, and the relative abundance of taxonomic and functional reads.

For bacterial species and each subsystem, shared and unique functions were visualised across all populations using petal diagrams.

To assess bacterial rarity differences across compartments, we classified species into abundance groups following Xue et al. (2018) based on their average relative abundance within each sampling site, for each plant compartment. Abundance‐based assessments are commonly used approaches to assess the abundance levels of species, especially rare taxa (RT) that are often influential contributors to ecosystem community dynamics (Lynch and Neufeld 2015; Pedrós‐Alió 2012). Species with ≥ 1% relative abundance within sites were considered abundant taxa (AT), those with < 0.01% as RT, and those in between as moderate taxa (MT). Species with ≥ 0.01% abundance across all sites but ≥ 1% in at least one site were classified as conditionally abundant taxa (CAT), while those always < 1% but < 0.01% in at least one site were conditionally rare taxa (CRT). Species ranging from < 0.01% in one site to ≥ 1% in another were considered conditionally rare and abundant taxa.

#### 2.4.2 Differential Abundance Analysis

We used differential abundance analysis to evaluate differences in bacterial phyla and functions across each of the sampled compartments (i.e., soils, rhizospheres and endospheres) using global and pairwise tests through the *ancombc2* function from the R package ANCOMBC on non‐normalised count data (H. Lin and Peddada 2020). Differential abundance across the aridity gradient was done by allocating samples to high, medium and low aridity categories (aridity index > 0.6 = low; > 0.4 but ≤ 0.6 = medium; and ≤ 0.4 = high aridity), using low aridity as the reference group.

#### 2.4.3 Canonical Correspondence Analysis (CCA)

To predict the environmental drivers of both species‐level taxonomic diversity and functional genes across our microbiomes, we ran CCA across all bulk soil, rhizosphere and endosphere samples (explanatory variables included are in Table S4). Correlated explanatory variables (*r* > 0.75) were removed. We then performed forward and backward selection of the included explanatory variables using the *ordistep* function in the R package, vegan (Oksanen et al. 2019).

### 2.5 Network Analysis

We conducted co‐occurrence network analysis of microbial functional processes within low, medium and high aridity categories (aridity index > 0.6, > 0.4–0.6 and ≤ 0.4). This analysis identified hub functions that may perform keystone roles supporting functional pathways, and shows the complex relationships among key taxonomic and functional properties of each plant compartment. Networks and hub functions were compared across bulk soils, rhizospheres and endospheres at the lowest functional level, focusing on positive or negative associations. Only functions with > 100 sequences were reported. Associations were calculated using SparCC correlations (edge thresholds, |SparCC| ≥ 0.3; *p* < 0.05), and significance was estimated via 200 permutations (Friedman and Alm 2012; Kurtz et al. 2015). Networks were visualised with the R packages *igraph* (Csardi et al. 2006), and within each network, the number of samples in the functional categories at subsystem level 1 ranged from 5 to 17 (Table S1). Hub functions were identified as the top 20 bacterial functions ranked by node degree and closeness centrality.

## 3 Results

### 3.1 Taxonomic and Functional Alpha Diversity

Across our samples and plant compartments, we generated taxonomic libraries with a total of 26 919 111 reads (~258 838 per sample), with 9835 unique bacterial species (~7998 per sample) (Table S5). Our functional libraries contained a total of 40 520 567 reads (~385 910 per sample), with 31 167 unique functions (~11 799 per sample) (Table S5; see Table S6 for proportions of reads across the six functional categories at SEED subsystem level 1).

We identified 13 phyla representing 98% of reads assigned to bacteria with abundance estimates greater than 1.5%, and represented 11.8% of all species (Figure 1a). Furthermore, we also found that 88.2% of species were rare versus the 11.8% of taxa > 0.1% relative abundance per sample across all sites and compartments. Furthermore, bacterial species were delineated into different rarity and abundance categories across each plant compartment (soil, rhizosphere and endosphere). Across all compartments, MT consistently accounted for the largest share of both species and reads, especially in soil and rhizosphere (~46%). In contrast, the endosphere was distinct, with a much higher proportion of RT, contributing over 35% of species and reads. Abundant (AT), conditionally abundant (CAT) and CRT were present at similar proportions in soil and rhizosphere, while their representation declined notably in the endosphere. This indicates a shift in community structure from dominance by moderate and conditionally AT in the soil and rhizosphere toward a higher relative contribution of RT in the endosphere, suggesting increased rarity and reduced prevalence of consistently abundant species deeper into the root‐associated compartments (Table 1).

**Table 1:** Bacterial ASVs allocated to six relative abundance categories.

Compartment	Abundance category	Number of species	Number of reads
Soil ~	Abundant taxa (AT)	5 (10.417%)	240 (10.417%)
	Conditionally abundant taxa (CAT)	7 (14.583%)	336 (14.583%)
	Conditionally rare taxa (CRT)	12 (25%)	576 (25%)
	Moderate taxa (MT)	22 (45.833%)	1056 (45.833%)
	Rare taxa (RT)	2 (4.167%)	96 (4.167%)
Rhizosphere ~	Abundant taxa (AT)	5 (10.417%)	240 (10.417%)
	Conditionally abundant taxa (CAT)	6 (12.5%)	288 (12.5%)
	Conditionally rare taxa (CRT)	9 (18.75%)	432 (18.75%)
	Moderate taxa (MT)	22 (45.833%)	1056 (45.833%)
	Rare taxa (RT)	6 (12.5%)	288 (12.5%)
Endosphere ~	Abundant taxa (AT)	5 (10.417%)	240 (10.417%)
	Conditionally abundant taxa (CAT)	2 (4.167%)	96 (4.167%)
	Conditionally rare taxa (CRT)	6 (12.5%)	288 (12.5%)
	Moderate taxa (MT)	18 (37.5%)	864 (37.5%)
	Rare taxa (RT)	17 (35.417%)	816 (35.417%)

We observed a decreasing effective number of bacterial species from bulk soil into the rhizosphere and the endosphere compartments for our *T. triandra* plants (mean effective no. species ±SD: bulk soils = 2935 ± 619, rhizospheres = 2491 ± 575, endospheres = 1824 ± 400; Table S7; Figure 1b; LMEM: df = 2, χ² = 95.27, *p* < 0.001). In addition, we found that bacterial community compositions were significantly different across each of the compartments (Table S8; Figure 1c) with aridity index (Table S2; Figure 1c), and with sampling population (Table S8; Figure S2). Furthermore, we found that the average distance to centroid values decreased from the rhizospheres into bulk soils, and endospheres (Table S9; Figure 1d; PermDispersions: *F*_(2,101)_ = 4.71, *p* = 0.011).

**Figure 2:**
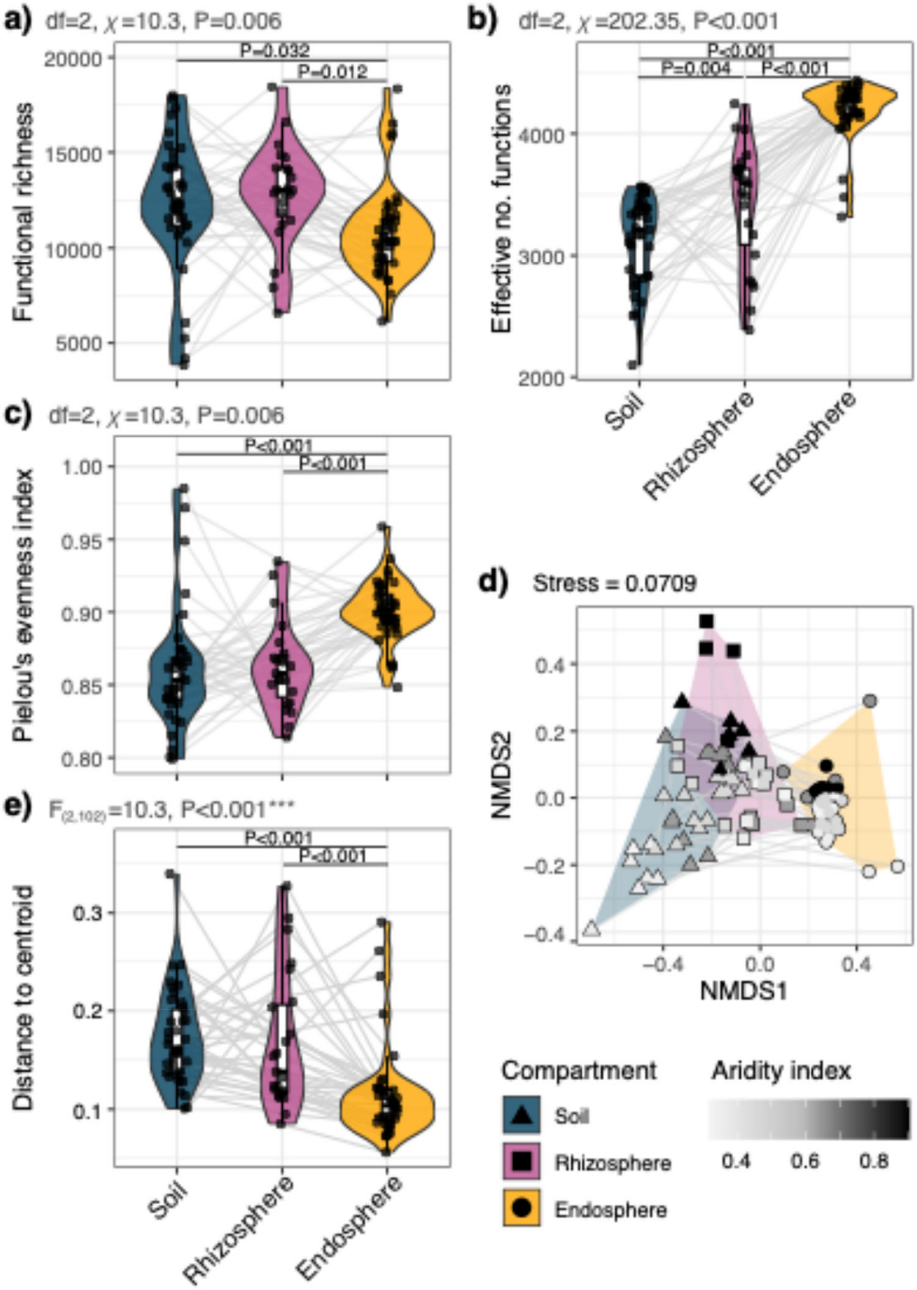
Alpha and beta diversity metrics for normalised functional gene abundances associated with plant compartments (bulk soil, rhizospheres and endospheres). Alpha diversity estimates for microbial genes associated within their plant compartments for (a) functional gene richness, (b) the effective number of functions and (c) Pielou's evenness index (see Table S10). Light grey lines connect the data points with shared plant ID across the bulk soils, rhizospheres and endospheres. Beta diversity for estimates across functional gene compositions with plant compartments is shown via (d) NMDS ordinations with Bray–Curtis distances (stress = 0.071; Table S8). Shape and hulls show the sample compartments, whereas point colour shows the aridity index. (e) Beta dispersion is represented by distance to centroid estimates (see Table S9). [Color figure can be viewed at wileyonlinelibrary.com]

There was decreasing richness of observed gene functions from bulk soils and rhizospheres into the endosphere (mean richness ±SD: bulk soils = 12 336 ± 3335, rhizospheres = 12 817 ± 2664, endospheres = 10 767 ± 2371) (Table S10; Figure 2a; LMEM: df = 2, χ² = 10.3, *p* = 0.005). However, the effective number of functions (the exponent of Shannon's diversity index) showed an increase in alpha diversity from bulk soil to rhizospheres, and then to endospheres (bulk soils = 3128 ± 356, rhizospheres = 3420 ± 503, endospheres = 4204 ± 230; Table S10; Figure 2b; LMEM: df = 2, χ² = 202.35, *p* < 0.001). Pielou's evenness index revealed that functional gene distributions were more even in endospheres than in rhizospheres and bulk soils (Table S10; Figure 2c; LMEM: df = 2, χ² = 43.45, *p* < 0.001). Together, these results show that when functional diversity is normalised using Shannon's diversity‐derived approaches (which account for higher evenness in the endosphere), it increases from bulk soil to the endosphere.

**Figure 3:**
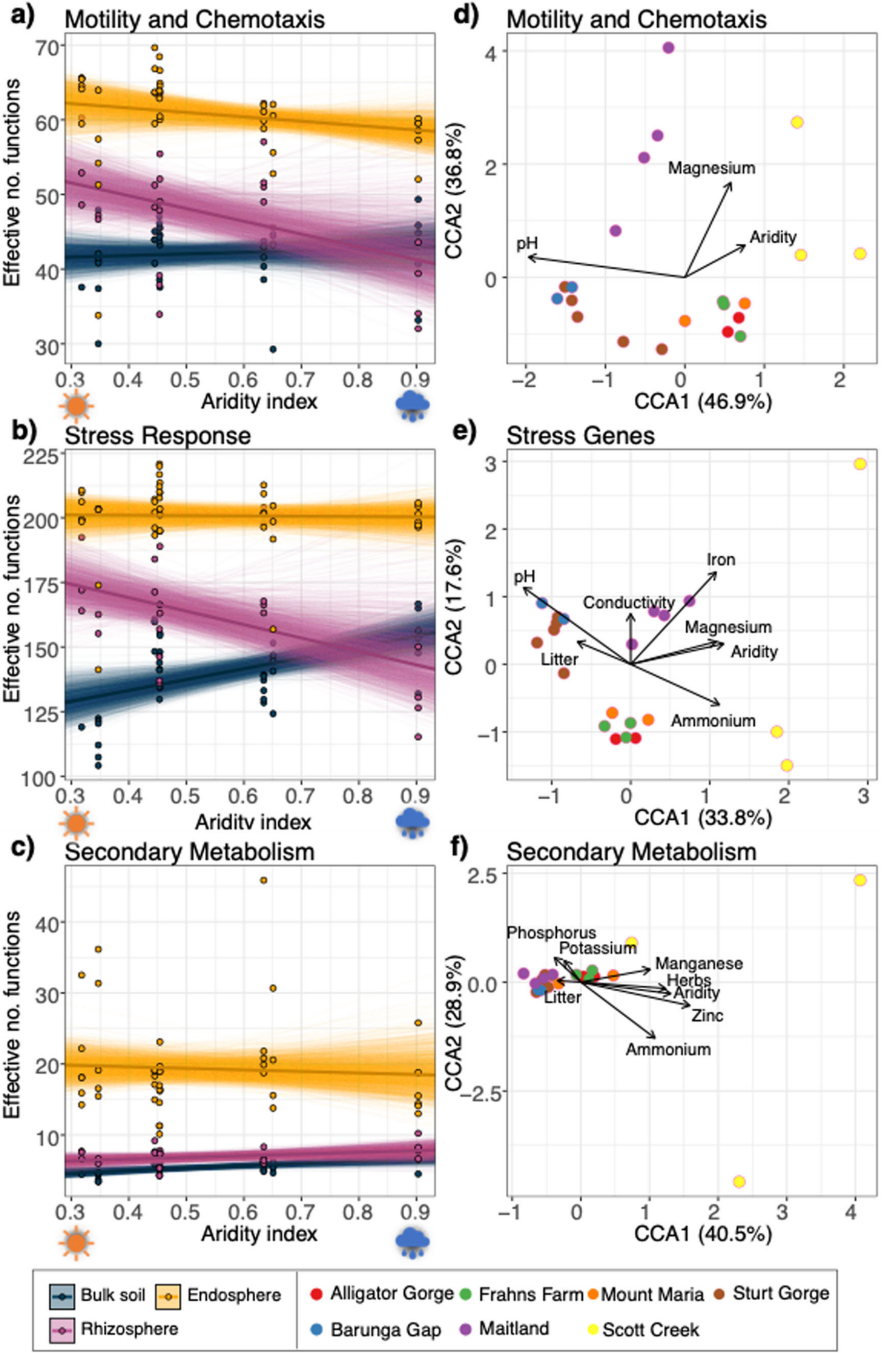
Changes in the diversity of functions in plant compartments and canonical correspondence analysis (CCA) showing the effect of the environmental variables on microbial functional structure in *T. triandra* rhizospheres. Aridity index is plotted against the alpha diversity for: (a) motility and chemotaxis, (b) stress response and (c) secondary metabolism, showing 2000 bootstrapped estimates (see Table S2 for all bootstrapped statistical output). CCAs show genes at subsystem level 1 attributed to (d) motility and chemotaxis, (b) stress response and (f) secondary metabolism. Vectors represent significant variables associated with gene community compositions. Points are coloured by sampling population. The remaining bulk soil, rhizosphere and endosphere CCAs are found in Figures S35–S37, see Table S4 for explanation of explanatory variables. [Color figure can be viewed at wileyonlinelibrary.com]

The motility, chemotaxis, and stress response, functional subsystems were associated with increased effective functional diversity in the rhizospheres with higher aridity levels (lower aridity index values) (Figure 3a,b; Table S2). We also observed the inverse trend for functional diversity of bulk soils in relation to stress response genes, which increased with decreasing aridity levels (high aridity index values) (Figure 3b; Table S2). However, responses to aridity varied across functional categories; for example, we did not see evidence of directional trends with the effective number of functions for secondary metabolism (Table S2; Figure 3c; Figures S3 and S4). Within the root compartments, we found a positive correlation between the effective number of species and functions, showing that within each compartment a greater number of bacterial species were associated with more functional genes (Table S11; Figure S5a; LMEM: χ² = 29.28, *p* < 0.001). However, this was a highly compartment‐dependent association—when compartment effects were not accounted for in the models, functions actually declined with species richness (Figure S5b).

### 3.2 Taxonomic and Functional Gene Compositions

Bulk soils, rhizospheres and endospheres produced distinct compositions of their functional gene profiles (Figure 2) that were affected by aridity (Figure 2d; also see Figures S6–S11). These compartment and aridity differences persisted across each of the subsystem profiles, including: motility and chemotaxis, stress response genes, nitrogen metabolism, phosphorus metabolism, regulation and cell signalling and secondary metabolism. However, our principal coordinates analyses (PCoA) showed a tendency toward saturation in the ordinance space, known as the horseshoe effect (see Morton et al. 2017). To account for this, we also examined variation along alternative PCoA axes (Axes 1, 2 and 3) (Table S12; Figures S12 and S13). Furthermore, the beta dispersions, or distance‐to‐centroid metrics, showing compositional variability within the whole functional communities also revealed increasingly similar compositions (i.e., tighter groupings) towards the endospheres, while soils and rhizospheres were comparatively varied (Figure 2e), and this relationship was also consistent within each functional subsystem, described above (Figure S14).

**Figure 4:**
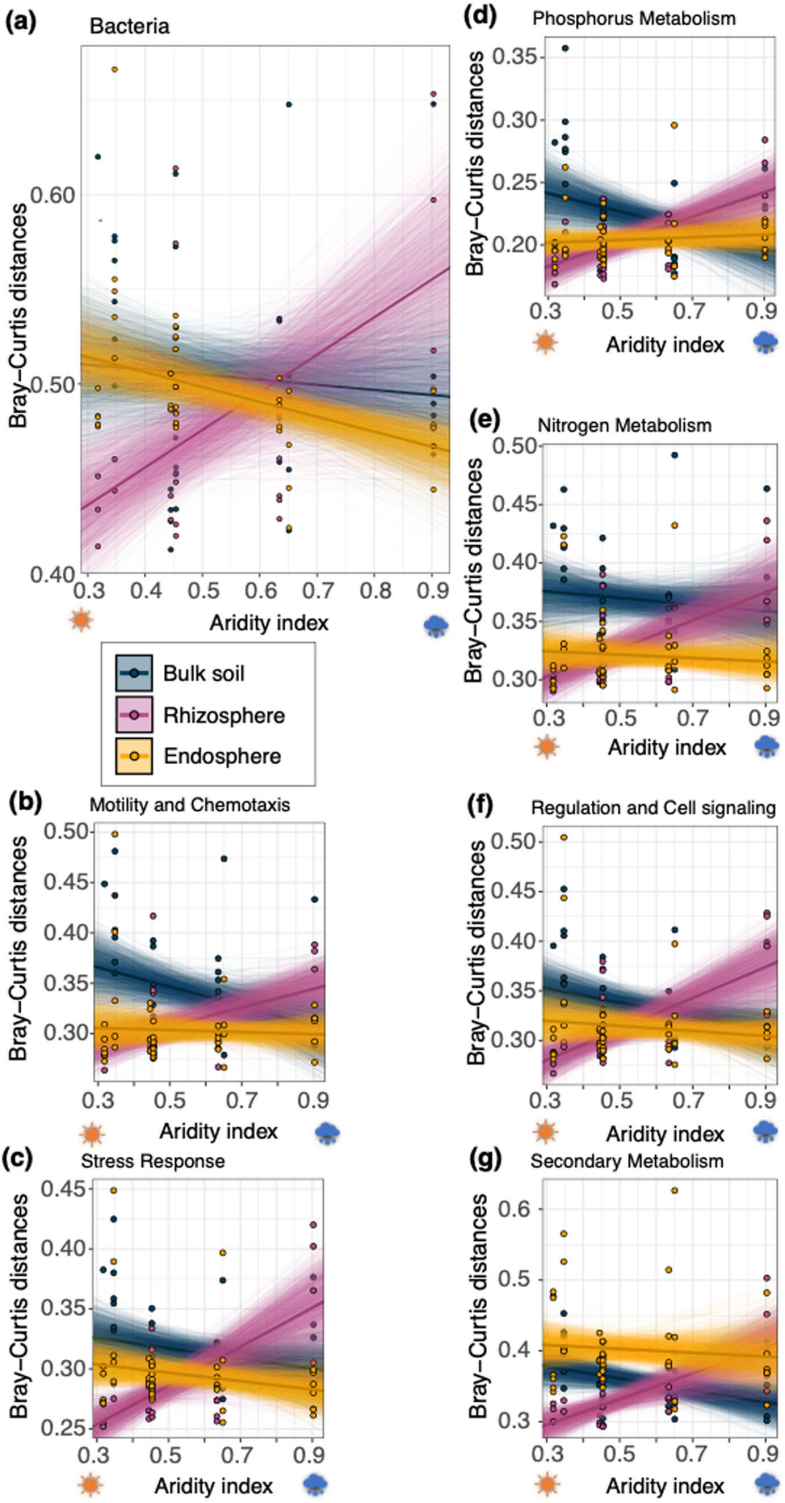
Aridity associates with the homogeneity of taxonomic and functional microbiomes differently across plant compartments. (a) Beta diversity of average Bray–Curtis distances of each bacterial taxonomic community, and beta diversity (Bray–Curtis distances) of functions at SEED subsystem level 1: (b) motility and chemotaxis, stress responses, phosphorus metabolism, nitrogen metabolism, regulation and cell signalling and secondary metabolism. For all comparisons, the mean estimate and 2000 bootstrapped estimates are plotted to give an indication of each relationship (see Tables S2 and S3 for all bootstrapped statistical output). [Color figure can be viewed at wileyonlinelibrary.com]

Aridity had a compartment‐specific association with the homogeneity of different taxonomic and functional communities based on the mean Bray–Curtis distances. For both the bacterial taxonomic and microbial functions (visualised using the six subsystem categories), the rhizosphere showed the strongest response to aridity, consistently becoming more dissimilar (i.e., heterogeneous) as aridity decreased (i.e., we see increases in the average Bray–Curtis distance with aridity index; Figure 4, see Tables S2 and S3 for details). In bulk soils, there was generally a positive correlation with aridity; communities became more similar, taxonomically and functionally, as aridity decreased (Figure 4, see Tables S2 and S3 for details). In endospheres, there was no meaningful change in the compositional distances for functions with aridity, although taxonomic compositions became slightly more homogenous as aridity decreased (Figure 4; Tables S2 and S3).

Overall, we consistently found that more bacterial species and functions in each subsystem were shared (i.e., found in at least one site of every population) than were isolated to each population alone (see Figures S15–S21).

### 3.3 Differentially Abundant Taxonomy and Functions

We found many differentially abundant bacterial phyla across the soils, rhizospheres and endospheres (Figure 1e; Table S13). Among those with the highest log fold change in abundance from the endosphere to rhizosphere, and endosphere to bulk soil were Actinomycetota (−1.794 and −2.691, respectively), Myxococcota (−1.02 and −1.984, respectively) and Pseudomonadota (−0.735 and −1.374, respectively) (Figure 1a,e). Comparatively, phyla that had a positive log fold change in abundances from the endospheres into the rhizospheres and soils were: Bacteroidota (0.929 and 1.231, respectively), Cyanobacteriota (0.712 and 1234, respectively), Ignavibacteriota (1.438 and 2.033, respectively), Atribacterota (1.151 and 1.915, respectively) and Chlamydiota (1.192 and 2.084, respectively) (among others) (Figure 1a,e; Table S13).

We focused our analysis on the differences between soils, rhizospheres and endospheres, primarily across three main functional subsystems: secondary metabolism, motility and chemotaxis and nitrogen metabolism (see Table S14 and Figures S22–S27 for all differences between soils, rhizosphere and endospheres at SEED subsystem 1). For secondary metabolism, there were more abundances of functional genes associated with plant a variety of growth hormones, defence and plant‐microbial interactions in *T. triandra* endospheres (see Figure S25). Within the motility subsystem, *T. triandra* endospheres also had more chemotaxis functions, but lower abundances of functions related to flagellar and non‐flagellar movement of bacteria (see Figure S22). Finally, we found that the abundances of nitrogen fixation functions decreased in the endospheres, but we found more functions related to nitric oxide synthase (see Figure S23). Differential abundance analysis showed changes in a low number of functions across the aridity gradient (see Figures S28–S33).

### 3.4 Canonical Correspondence Analysis

Our CCAs revealed variable associations between all soil physicochemical and environmental variables with bacterial taxonomic and functional communities within each of the *T. triandra* microbial compartments. After highly correlated variables were removed (Figure S34a), the taxonomic bulk soil communities were associated with pH, aridity, shrubs and *T. triandra* density (Figure S34b). In the rhizospheres, bacterial community compositions corresponded with aridity, pH, magnesium and litter levels (Figure S34c). In the endospheres, however, bacterial communities had significant structural associations with aridity, electrical conductivity, calcium and copper levels, in addition to pH, graminoids and canopy cover (Figure S34d).

The functional CCAs corresponding to the remaining subsystems revealed numerous other environmental associations (Figures S35–S37; Tables S4). The rhizosphere functions associated with motility were significantly structured by pH, magnesium and aridity (Figure 3d). In the stress responses, we observed important drivers from pH, ammonium, electrical conductivity, litter, aridity, iron and magnesium levels (Figure 3e). Finally, the secondary metabolism functions, which included plant hormone functions (among others), corresponded with ammonium, potassium, phosphorus, electrical conductivity, zinc and magnesium, and herbs (Figure 3f).

### 3.5 Network Analysis

**Figure 5:**
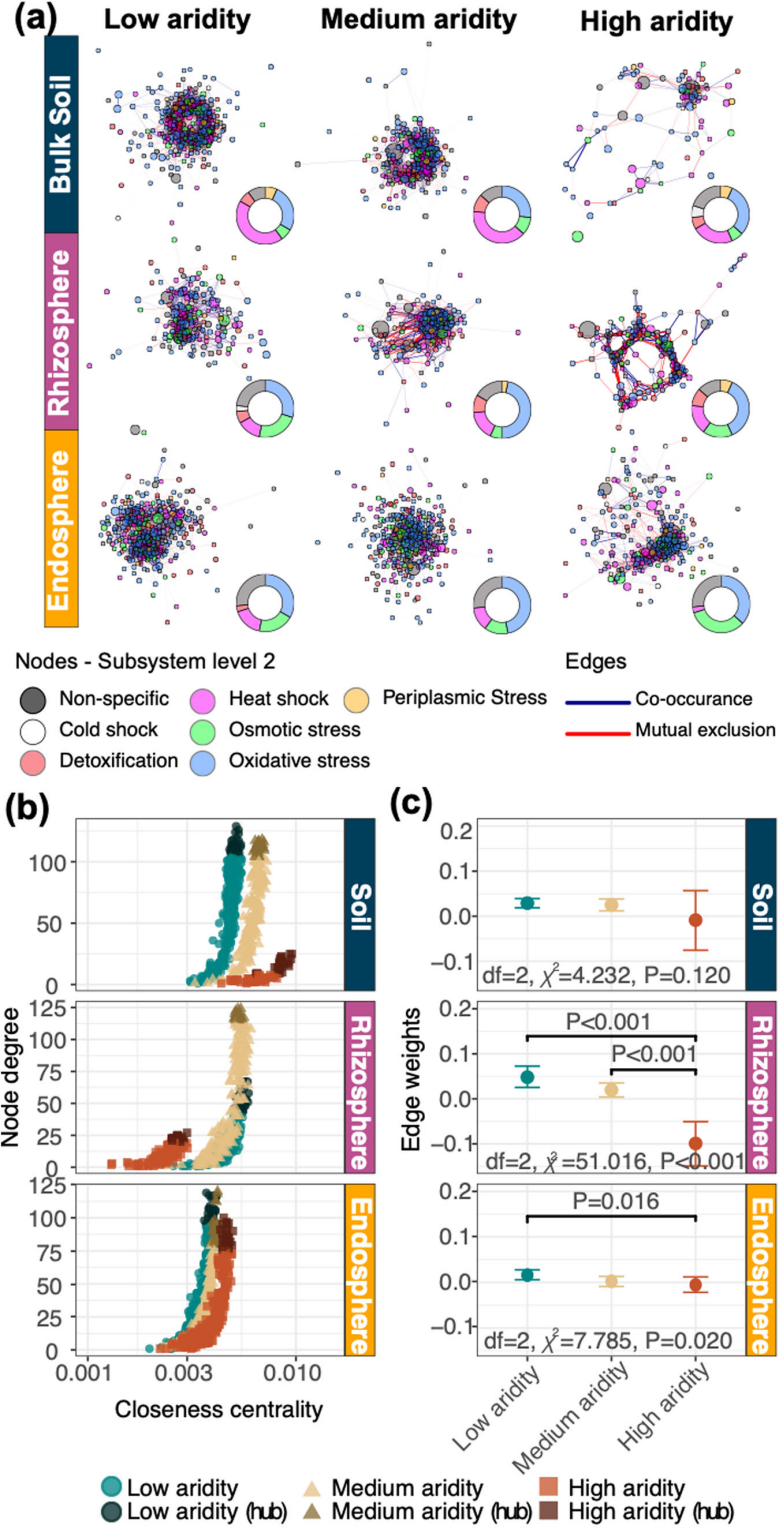
Network analysis showing the stress response functions across *T. triandra* compartments (bulk soil, rhizosphere and endosphere), across low, medium and high aridity levels. (a) Each network analysis comprises nodes representing functional processes of stress response genes, coloured at the subsystem level 2. Node size shows the relative abundance of each function, connected via positive (blue) or negative (red) edges. Doughnut plots indicate the proportion of functions at subsystem level 2 for each network. (b) Hub functions were chosen as the top‐ranked functions by node degree and closeness centrality (Table S16). (c) Mean edge weights with upper and lower CIs showing the degree of positive (co‐occurrences) versus negative associations (mutual exclusion) between functions in low to high aridity across all plant compartments. For reporting of all statistical output for Kruskal–Wallis tests, see Table S15. [Color figure can be viewed at wileyonlinelibrary.com]

**Figure 6:**
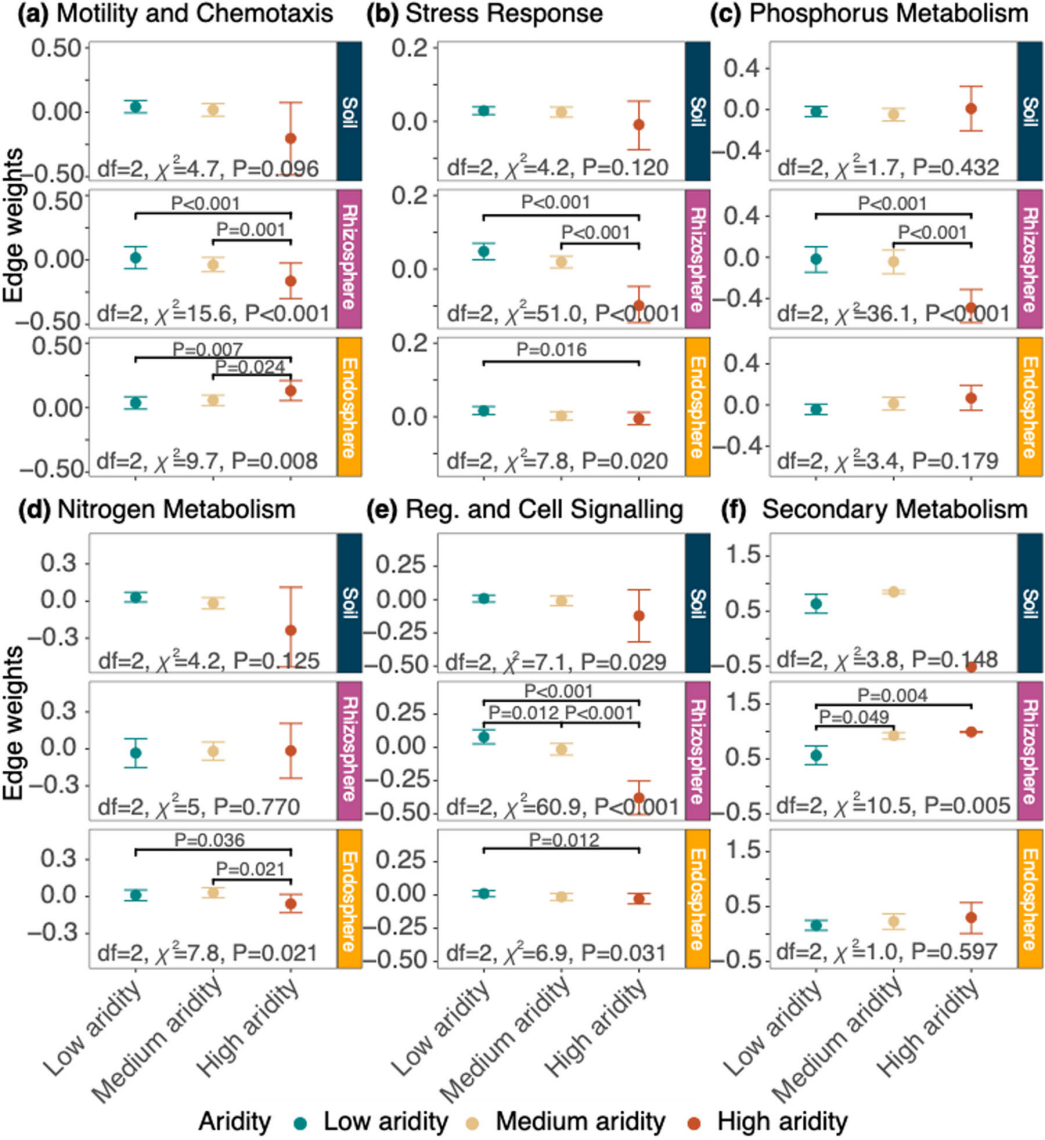
Network analysis edge weights across six functional levels in *T. triandra* microbial compartments (bulk soil, rhizosphere and endosphere), across low, medium and high aridity levels. Mean edge weights with upper and lower CIs showing the degree of positive (co‐occurrences) versus negative associations (mutual exclusion) in (a) motility and chemotaxis, (b) stress response, (c) phosphorus metabolism, (d) nitrogen metabolism, (e) regulation and cell signalling and (f) secondary metabolism. For reporting of all statistical output for Kruskal–Wallis tests, see Table S15. [Color figure can be viewed at wileyonlinelibrary.com]

We conducted co‐occurrence network analysis across six gene function categories, three plant compartments (soil, rhizosphere, endosphere) and three aridity levels (low, medium, high) (Table S1), focusing on stress response functions (Figure 5). This approach revealed how key bacterial and functional properties vary across plant compartments and aridity levels, highlighting components that likely contribute to important metabolic processes. Mean edge weights across the remaining five functional subsystems were also analysed (Table S15; Figure 6). In bulk soil, stress response function networks had higher closeness centrality in high‐aridity networks (0.0072 ± 0.0013 SD, *n* = 7) compared to low and medium aridity networks (0.0062 ± 0.0006, *n* = 13; 0.0047 ± 0.0005, *n* = 16, respectively). Rhizosphere centrality was lower in high aridity (0.0024 ± 0.0003, *n* = 5) than in the medium (0.0049 ± 0.0007, *n* = 9) and low (0.0046 ± 0.0007, *n* = 9) aridity networks (Figure 5b). The endosphere centrality remained consistent across the high, medium and low aridity levels (0.0039 ± 0.0006, *n* = 11; 0.0035 ± 0.0004, *n* = 17; and 0.0034 ± 0.0004, *n* = 17, respectively). In all the bulk soils, rhizospheres and endospheres, mean node degree generally decreased from the medium (61.5 ± 34.1 SD, *n* = 16; 63.0 ± 42.8, *n* = 9; 30 ± 26.2, *n* = 17, respectively) and low aridity (63.9 ± 35.4, *n* = 13; 21.7 ± 17.1, *n* = 9; and 40.8 ± 31.2, *n* = 17, respectively), into the high aridity (8.8 ± 6.1, *n* = 7; 12.1 ± 6.8, *n* = 5; and 32.5 ± 28.2, *n* = 11, respectively) networks (Figure 5b).

The top 20 hub functions in each aridity and compartment network (Table S16) primarily related to heat stress in soils, accounting for 43%, 40% and 23% of the low, medium and high aridity networks, respectively (Figure 5). Oxidative stress hub functions were also important, comprising 30% in low aridity rhizosphere networks and increasing to 47% and 37% in medium and high aridity networks, respectively. In endosphere networks, oxidative stress functions comprised 33% of the hub functions in low aridity networks, rising to 47% and 37% in medium and high aridity, respectively (Figure 5). The proportion of osmotic stress hub functions increased from 7% in soil to 17% in rhizosphere and 33% in endosphere high aridity networks. Comparatively, in the low‐aridity networks, osmotic stress comprised 7%, 23% and 20% of soil, rhizosphere and endosphere hub functions, respectively, while in medium aridity networks, it comprised 10%, 7% and 13%, respectively (Figure 5).

## 4 Discussion

This study advances our understanding of plant‐root colonisation by soil microbiota in wild plant roots by using shotgun metagenomics on soils, rhizospheres and endospheres of *T. triandra*—a globally important C4 grass species. We report clear evidence that the two‐step selection process—the colonisation of microbes first from bulk soils into plant rhizospheres, and then from rhizospheres into endospheres via host plant regulation— was acting on bacterial communities across an aridity gradient. Most endosphere microbiota were a subset of those in the bulk soil, supporting our first hypothesis. Surprisingly, the predicted microbial functions showed the opposing trend—a novel finding that goes against our second hypothesis, and highlights the enriched diversity (Shannon's index and evenness) of microbial functions recruited by host plants. In support of our third hypothesis, we also show that increasing aridity modulated taxonomic and functional recruitment by host plants, most noticeably influencing rhizospheres. These communities became more similar and exhibited highly diverse stress response functions. As such, *T. triandra* plants likely benefit from microbiota via the retention of a diverse suite of potential microbial functions under more arid conditions (particularly stress response genes). By expanding the two‐step selection model to incorporate potential microbial functions, our study not only advances our knowledge of plant‐soil ecology in wild *T. triandra* populations but also provides applications to the restoration of grasslands.

### 4.1 Contrasting Taxonomic and Functional Microbial Colonisation

We show that bacterial taxonomic alpha diversity decreased from bulk soils into rhizospheres, then into root endospheres, supporting our first hypothesis and aligning with previous findings for the two‐step selection process (Bulgarelli et al. 2012; Lundberg et al. 2012), and global trends (Ling et al. 2022). However, we observed that microbial functions showed the opposite trend; the diversity of potential functions increased from bulk soil to rhizospheres and into the endospheres. This enriched functional diversity is likely produced by the increased evenness (i.e., via Pielou's evenness index) among different types of functions predicted within the endospheres. The relative abundance weighting in Shannon's diversity index means that greater evenness of predicted functions leads to higher overall diversity. Given the significant changes in microbial profiles observed from rhizospheres into endospheres, this new insight likely reflects substantially different functional requirements of the host plant across these two compartments, and the strong influence of the plant's immune system (Adeleke et al. 2021). While we acknowledge that our functional and taxonomic annotations may be subject to biases, our study contributes valuable new insights into how the two‐step selection process operates through a functional ecology lens.

### 4.2 Variation in Endosphere Functional Profiles

We report that endosphere functional and taxonomic profiles converged into more homogeneous communities than in bulk soils and rhizospheres. This suggests that *T. triandra* strongly regulates the entry of microbiota into its endospheres, maintaining a common functional capacity, despite wide geographic distances and varying levels of aridity. We observed converging patterns in endosphere functional communities across almost all functional groupings measured at subsystem level 1 (i.e., motility and chemotaxis, nitrogen metabolism, phosphorus metabolism, regulation and cell signalling and stress response functions). Only secondary metabolism functions were more heterogeneous in root endospheres compared to rhizospheres and bulk soils (discussed below). The selection pressures for endosphere colonisation are likely driven by the functional needs of *T. triandra* and host traits promoting mutually beneficial interactions (Bulgarelli et al. 2013).

All endosphere samples consistently showed increases in flagellar movement and chemotaxis functions, which are important for the movement and navigation of bacteria (Bulgarelli et al. 2013), and are enriched in rhizspheres of tomatoes (*Solanum lycopersicum*) (Barajas et al. 2020). Endospheres also became more similar by excluding functions like microbial gliding, which help bacteria travel through biofilms and are commonly found in rhizospheres and soils (Bhattacharyya et al. 2023). Comparable trends occurred for nitrogen metabolism, where we found increased abundances of genes associated with nitric oxide synthases in the endospheres—crucial for signalling between plants and their associated microbiota, and which play key roles in helping plants respond to oxidative and drought stress, although they may also aid microbiota in tolerating the host plant's immune system (Shah et al. 2023). Conversely, nitrogen‐fixing functions were less abundant in endospheres, being more prevalent in bulk soils where they are involved in well‐described processes within the nitrogen cycle, aligning with global trends observed in rhizosphere studies (Stein and Klotz 2016; Ling et al. 2022). These functional roles may differentially affect the fitness of colonising microbiota within the endosphere (e.g., optimised movement and navigation) while contributing to improved host growth and fitness (e.g., enhanced stress responses). Furthermore, the consistent recruitment and exclusion of microbial functions into the endospheres by host plants suggest strong symbiotic community assembly processes.

Unlike motility and nitrogen metabolism, the secondary metabolism functions were more heterogeneous in *T. triandra* endospheres compared to the rhizospheres and soils. Secondary metabolism functions are typically involved with survival adaptations, defence or derived environmental responses and are not necessarily essential for growth (Khare et al. 2018; Srivastava and Raghuwanshi 2023). Specifically, we observed changes in functions across biosynthesis and degradation of key metabolites like auxins, flavonoids and phenylpropanoids, which likely support plant responses to environmental stresses and pathogens, and maintain structural integrity during growth (Kincses et al. 2024). We also suspect that functions associated with clavulanic acid and phenazine metabolites may shape plant‐microbe interactions due to their noted associations with antibiotics (Wang et al. 2021). Strong regulation of microbial entry into endospheres is observed in many plant species and can be influenced by factors including growth stage, genotype and geography (Lumibao et al. 2022). Nevertheless, the high‐endosphere variation in secondary metabolism functions provides a unique and novel finding that highlights the diverse ecological needs of different *T. triandra* populations across their natural distribution.

### 4.3 Aridity and Rhizosphere Associations

We show that taxonomic and functional diversity of *T. triandra* rhizospheres increased with aridity, but not in soils or endospheres. This increased diversity was found across stress responses, motility and nitrogen metabolism, including functions related to (but not limited to) oxidative stress, flagellar movement of microbiota and nitric oxide synthases. Functional diversity and ecological complexity are often associated with ecosystem stability and resilience (Oliver et al. 2015; Thorogood et al. 2023; Guo et al. 2024). Accordingly, greater predicted functional diversity in *T. triandra* rhizospheres may reflect environmental filtering or host‐mediated selection for microbiomes that confer functional stability and resilience under arid conditions, thereby buffering plants against vulnerability to water stress (Louca et al. 2018; Ling et al. 2022). This dynamic could support a stable and efficient network of metabolic processes (Puente‐Sánchez et al. 2024; Ramond et al. 2025).

We require additional studies to more confidently establish the ecological relationship between microbial taxa and functions in these plant compartments. That is, to determine whether the observed patterns reflect functional redundancy of higher‐level metabolic traits or functional complementarity (e.g., through niche differentiation among community‐aggregated traits or non‐overlapping functions) (Blüthgen and Alexandra‐Maria 2011; Ramond et al. 2025). This could be achieved using metagenome‐assembled genomes, which provide both taxonomic and functional characterisation of microbial genomes (Thorogood et al. 2023; Ramond et al. 2025). Likewise, meta‐transcriptomic or proteomic data can directly measure active metabolic processes occurring between microbiota and host plants (Armengaud 2023; Ramond et al. 2025). These approaches could show whether functional stability arises from increased buffering capacity in response to environmental change (altered biotic or abiotic conditions), and whether taxa involved are functional generalists or specialists.

In the *T. triandra* bulk soil communities, we found that alpha diversity of the stress response functions was lower for arid populations. Previous work has shown that host/environment‐driven changes more strongly shape microbiomes of drier grasslands over temperate grasslands (Zhong et al. 2022). Furthermore, endosphere diversity is seen to change with plant needs across species in extreme environments (Harrison and Griffin 2020). Indeed, a meta‐analysis by Ling et al. (2022) has shown that rhizosphere microbial communities are also enriched in functional traits compared to bulk soils, particularly under environmental stress. Our findings highlight the impact *T. triandra* has on its rhizospheres to potentially counter stress from elevated aridity by recruiting high microbial functional diversity around its roots.

In lower aridity populations, we also found that rhizospheres were more heterogeneous compared to those in higher aridity environments (increasing community dissimilarity). Coupled with the higher alpha diversity in arid rhizospheres, this suggests that higher aridity promotes microbial communities that span a similar taxonomic range while being functionally diverse, a complexity not seen in the general soil environment (Lumibao et al. 2022). Evidence of stronger microbial selection pressures reinforces our third hypothesis that arid conditions alter host plant recruitment and colonisation dynamics. Overall, we provide new evidence of these dynamics in the C4 grass *T. triandra*, with microbe‐mediated assistance under natural arid conditions.

### 4.4 Stress Response Functions With Varying Aridity

In our stress response network analysis, the hub functions highlight that the microbiomes across bulk soils, rhizospheres and endospheres likely have mechanisms to cope with heat shock, oxidative stress and osmotic stress—especially in high aridity populations. Oxidative stress responses are important mechanisms for plants to thrive in low water conditions by addressing the buildup of reactive oxygen species, which can be toxic to plants and tend to accumulate in tissues when under environmental stress (Berrios and Rentsch 2022). Furthermore, osmotic stress directly affects cellular water potential responses, which are crucial for sustaining microbial community resilience and functioning under dehydration (Bremer and Krämer 2019). Ultimately, the hub functions associated with heat shock, oxidative and osmotic stress likely facilitate important connections that help *T. triandra* and its microbiota to thrive in hot, arid environments. Future studies should more explicitly investigate how metabolic processes linked to water availability and changing temperatures shape the structure and function of *T. triandra*‐associated soil microbial communities.

Across the stress response and regulation and cell signalling networks, edge weights were unexpectedly more negative in high aridity populations of the rhizospheres and endospheres, indicating mutual exclusion among functional processes. This could be due to heightened resource competition among microbiota within our gradient, particularly if highly specialised functions are being selected at the expense of others (i.e., strong niche partitioning) (Q. Lin et al. 2021). Only the rhizosphere secondary metabolism genes, and endosphere motility and chemotaxis genes had more positive edge weights in the high aridity networks, compared to low aridity networks. This suggests convergence towards functional niches, or more cooperation across functional processes with stronger mutualistic relationships between microbiota (e.g., possibly involving more complex and interconnected pathways of organic molecule synthesis and/or degradation).

### 4.5 Patterns of Community Variation

We found that only a small proportion of species and functions were unique to any given sampling population or compartment. This contrasts with previous amplicon‐based studies, which have shown more unique taxonomic variation among *T. triandra* populations (Hodgson et al. 2025). It is possible, however, that these differences might arise due to differences in taxonomic resolution of the two approaches. Despite the high occurrence of functions common to the endospheres, we still observed significant functional community differentiation across populations. While there appear to be commonalities in the availability of microbial functions to host plants, there was still strong community differentiation across endosphere profiles in each population, driven by changes in soils and vegetation (Fitzpatrick et al. 2018).

## 5 Conclusion

Our study reveals key functional differences within *T. triandra* root systems across an aridity gradient. We found that endospheres exhibit higher functional diversity than both rhizospheres and bulk soils. This arose due to expanded functional evenness within endospheres, despite declines in functional richness through the two‐step selection process. Furthermore, changes in alpha and beta diversity suggest that in arid populations, rhizospheres foster increasingly homogenous microbiomes with high functional diversity, which likely bolsters the resilience of *T. triandra* to water‐limited environments by supporting key microbial functions under stressful conditions. Ultimately, *T. triandra* actively facilitates symbioses with microbiota in its rhizospheres and endospheres by driving specific functional profiles that impact host metabolism and select for high microbial fitness. These findings advance our understanding of functional plant‐microbial dynamics in grasslands and offer new insights for the restoration and management of grasslands under climate change.

## Conflicts of Interest

The authors declare no conflicts of interest.

## Supporting information

PCE‐Supplementary Information‐PR2‐clean2.

## Data Availability

The data supporting the findings of this study are openly available on Figshare at https://doi.org/10.25451/flinders.27619635, Sequence data are available in the NCBI Sequence Read Archive (SRA) at PRJNA1332270.
