# Supplementary material for: Contrasting Microbial Taxonomic and Functional Colonisation Patterns in Wild Populations of the Pan‐Palaeotropical C4 Grass, Themeda triandra
Source: Plant Cell Environ. 2025 Sep 26;49(1):209–25. doi: 10.1111/pce.70205 (PMC12675983; doi:10.1111/pce.70205)
Supplement: Supplementary file 1 — PCE‐Supplementary Information‐PR2‐clean2. [file PCE-49-209-s001.docx]

**SUPPLEMENTARY METHODS**

*Global* T. triandra *distribution with aridity*

We compared global aridity distributions of *T. triandra* to our sample sites using the R package terra (Hijmans, 2023) based on aridity data from version 3 of the Global Aridity Index and Potential Evapotranspiration Database (Global-AI_PET; Fig. 1c; Zomer et al., 2022). We then compared global occurrences of *T. triandra* based on records from the Global Biodiversity Information Facility between 2000 and 2023 (GBIF.org, 2023). Using the Atlas of Living Australia aridity index data, we found that our sampled aridity gradient covered 87% of all global occurrences (Belbin, 2011), while reevaluating our sampling sites using values from the Global-AI_PET database, the aridity gradient covered 41% of the recorded global occurrence (Fig. 1d; Zomer et al., 2022). This discrepancy may be due to data smoothing in the coarser global climatic data compared to more detailed fine-resolution patterns in the Atlas of Living Australia data. However, we argue this allows for a well-replicated and representative aridity gradient within which we could study *T. triandra* microbial dynamics.

*Vegetation surveys*

At each study population (Figure S1a, e), *T. triandra* density estimates and vegetation assessments were taken alongside plant and soil samples within 25 m x 25 m quadrats. *T. triandra* density was measured from five 4 m x 4 m quadrats within the target area. To characterise vegetation at each population, we ran six point-intercept transects within our 25 x 25 m quadrats (Bonham 2013). Each transect was spaced 5 m apart in a North-South direction and involved observations of the occurrence of plant species found every meter. Functional categories for the vegetation were as follows: graminoids, herbs (forbs), shrubs, trees/canopy cover, litter, and bare earth (exposed dirt or rock). Where more than one functional unit occurred at a given point, all were recorded (Figure S1f).

*Analysis of plant and soil physicochemical conditions*

We collected *T. triandra* leaf samples and analysed them for nutrient concentrations, as were the bulk soil samples that were collected at 30 cm from the base of these plants, along with other physicochemical conditions. Soil physicochemical conditions at 30 cm from host included: phosphorus and potassium (Colwell 1965), sulphur (KCl 40 method) (Blair et al. 1991), organic carbon (Walkley and Armstrong 1934), nitrate, ammonium, electrical conductivity and pH (CaCl_2_) at CSBP Laboratories (Bibra Lake, Australia), in addition to mean gravimetric water content (McPherson et al. 2018), which correlated with mean aridity index values across sampling sites (Figure S4). Nutrient analysis in *T. triandra* root and leaf samples were also conducted at CSBP Laboratories (Bibra Lake, Australia) using inductively coupled plasma (ICP) spectroscopy to measure trace elements and macronutrients within the plant, including: nitrogen, phosphorus, potassium, sulphur, copper, zinc, manganese, calcium, magnesium, sodium, iron, and boron.

**Figure S1. Sampling sites and aridity index with schematic of *Themeda triandra* plant compartments**. (a) Pan-palaeotropical distribution of *T. triandra* based on observations (points) from the Global Biodiversity Information Facility between years 2000-2023 (GBIF.org 2023). Points are likely underrepresented geographically across its distribution due to different practises of obtaining reliable records of occurrence. The colour gradient represents mean annual aridity index from Version 3 of the Global Aridity Index and Potential Evapotranspiration Database (Zomer et al. 2022). (b) Map of sampling sites (points) with aridity index with sourced from the Global AI-PET database (version 3), (c) Mantel test showing no correlation between comparisons of site-site geographic distances and aridity distances across each of the sampling sites (Mantel: *p* = 0.489; r = -0.021). (d) Density plot of global *T. triandra* occurrences based on records from the Global Biodiversity Information Facility between the years 2000–2023 with upper and lower limits from the Atlas of Living Australia aridity index data which included 87% of *T. triandra* occurrences (blue bar), and the Global AI-PET database (version 3) which included 41% of *T. triandra* occurrences (red bar). (e) Diagram of *T. triandra* plant compartments showing bulk soil, rhizosphere, and endospheres microbiota. (f) Photographs of sampling populations across (i) Alligator Gorge, (ii) Burunga Gap, (iii) Frahn’s Farm, (iv) Neagles Rock, (v) Maitland, (vi) Mount Maria, (vii) Scott Creek, and (viii) Sturt Gorge. (g) Bar plot of relative abundance of functional vegetation groups in each site. Functional categories for the vegetation were as follows: graminoids, forbs (herbs), shrubs, trees/canopy cover, litter, and bare earth (exposed dirt or rock).

**Figure S2. Non-metric multidimensional scaling ordination with Bray-Curtis distances showing bacterial taxonomic community differences across sampling sites.** Plots represent: (a) bulk soils (triangles), (b) rhizospheres (squares), and (c) endospheres (circles) (see Table S4).

**Figure S3. Alpha diversity with aridity index.** Effective number of functions across plant compartment (bulk soil=blue, rhizosphere=pink, endosphere=yellow). Functional annotations include: (a) motility and chemotaxis, (b) stress genes, (c) nitrogen metabolism, (d) phosphorus metabolism, (e) regulation and cell signalling, and (f) secondary metabolism. For full statistical output, see Table S2

**Figure S4. Functional richness across mean aridity index.** Colour represents plant compartment (bulk soil=blue, rhizosphere=pink, endosphere=yellow) across several key functional gene categories. Functional annotations include: (a) motility and chemotaxis, (b) stress genes, (c) nitrogen metabolism, (d) phosphorus metabolism, (e) regulation and cell signalling, and (f) secondary metabolism. For full statistical output, see Table S2

**Figure S5. The alpha diversity of bacterial species of samples is correlated with the alpha diversity of microbial functions.** (a) Effective number of species for each plant compartment (bulk soil=blue, rhizosphere=pink, endosphere=yellow) increases with their effective number of functions. (b) An increase in effective number of species for all samples is correlated with a general decrease in the effective number of functions. Effective number of species/functions represents the exponential transformation of Shannon’s diversity index. Density plots above the x and y axes, represent the distribution of samples showing the differences in median values across each compartment. For full statistical output, see Table S11.

**Figure S6.** **Relative abundance of microbial functional processes pertaining to motility and chemotaxis genes across all bulk soil, rhizosphere, endosphere samples.** Sample labels are coloured by aridity index of sampling sites, whereas bar labels indicate plant compartment (bulk soil = blue, rhizosphere = pink, and endosphere = yellow).

**Figure S7.** **Relative abundance of microbial functional processes pertaining to nitrogen metabolism across all bulk soil, rhizosphere, endosphere samples.** Sample labels are coloured by aridity index of sampling sites, whereas bar labels indicate plant compartment (bulk soil = blue, rhizosphere = pink, and endosphere = yellow).

**Figure S8.** **Relative abundance of microbial functional processes pertaining to phosphorus metabolism across all bulk soil, rhizosphere, endosphere samples**. Sample labels are coloured by aridity index of sampling sites, whereas bar labels indicate plant compartment (bulk soil = blue, rhizosphere = pink, and endosphere = yellow).

**Figure S9. Relative abundance of microbial functional processes pertaining to secondary metabolism across all bulk soil, rhizosphere, endosphere samples**. Sample labels are coloured by aridity index of sampling sites, whereas bar labels indicate plant compartment (bulk soil = blue, rhizosphere = pink, and endosphere = yellow).

**Figure S10.** **Relative abundance of microbial functional processes pertaining to regulation and cell signalling across all bulk soil, rhizosphere, endosphere** **samples**. Sample labels are coloured by aridity index of sampling sites, whereas bar labels indicate plant compartment (bulk soil = blue, rhizosphere = pink, and endosphere = yellow).

**Figure S11.** **Relative abundance of microbial functional processes pertaining to stress responses across all bulk soil, rhizosphere, endosphere samples**. Sample labels are coloured by aridity index of sampling sites, whereas bar labels indicate plant compartment (bulk soil = blue, rhizosphere = pink, and endosphere = yellow).

**Figure S12. Principal coordinates analysis with Bray-Curtis distances for six different functional gene categories annotated to subsystem 1, showing principal coordinates 1 (axis.1) and 2 (axis.2)**. Point shape and hull colours represent the samples belonging to the different plant compartments (bulk soil =blue, rhizosphere = pink, endosphere =yellow), whereas point colour shows the mean annual aridity index. Functional annotations include: (a) motility and chemotaxis, (b) stress genes, (c) nitrogen metabolism, (d) phosphorus metabolism, (e) regulation and cell signalling, and (f) secondary metabolism (see Table S12 for statistical output on distance to centroid estimates of samples to their respective compartments).

** Figure S13. Principal coordinates analysis with Bray-Curtis distances for six different functional gene categories annotated to subsystem 1, showing principal coordinates 2 (axis.2) and 3 (axis.3)**. Point shape and hull colours represent the samples belonging to the different plant compartments (bulk soil =blue, rhizosphere = pink, endosphere =yellow), whereas point colour shows the mean annual aridity index. Functional annotations include: (a) motility and chemotaxis, (b) stress genes, (c) nitrogen metabolism, (d) phosphorus metabolism, (e) regulation and cell signalling, and (f) secondary metabolism (see Table S12 for statistical output on distance to centroid estimates of samples to their respective compartments).

**Figure S14. Distance to centroid estimates for functional genes annotated to subsystem 1, based on principle coordinates analyses (PCoA).** Colour represent the samples belonging to the different plant compartments (bulk soil =blue, rhizosphere = pink, endosphere =yellow). Functional annotations include: (a) motility and chemotaxis, (b) stress genes, (c) nitrogen metabolism, (d) phosphorus metabolism, (e) regulation and cell signalling, and (f) secondary metabolism**.** See Table S12 for full statistical output.

**Figure S15. Counts of unique and shared bacterial species across each *T. triandra* compartment and site.** (a) Venn diagram showing unique and overlapping bacterial species across bulk soils rhizospheres and endospheres**.** Petal diagram showing counts that reveal which species are unique or common to each sampling site across: (b) bulk soils, (c) rhizospheres and (d) endospheres.

**Figure S16. Petal diagram** **showing counts for motility and chemotaxis functions.** Counts show which functions are unique within each site or common to all sites across: (a) bulk soils, (b) rhizospheres and (c) endospheres.

**Figure S17. Petal diagram** **showing counts for nitrogen metabolism functions.** Counts show which functions are unique within each site or common to all sites across: (a) bulk soils, (b) rhizospheres and (c) endospheres.

**Figure S18. Petal diagram** **showing counts for phosphorus metabolism functions.** Counts show which functions are unique within each site or common to all sites across: (a) bulk soils, (b) rhizospheres and (c) endospheres.

**Figure S19. Petal diagram** **showing counts for secondary metabolism functions.** Counts show which functions are unique within each site or common to all sites across: (a) bulk soils, (b) rhizospheres and (c) endospheres.

**Figure S20. Petal diagram** **showing counts for regulation and cell signalling functions.** Counts show which functions are unique within each site or common to all sites across: (a) bulk soils, (b) rhizospheres and (c) endospheres.

**Figure S21. Petal diagram** **showing counts for stress response functions.** Counts show which functions are unique within each site or common to all sites across: (a) bulk soils, (b) rhizospheres and (c) endospheres.

**Figure S22. Heatmap of differentially abundant motility and chemotaxis functions annotated at subsystem level 3.** Comparisons show the differences across plant compartments (bulk soils, rhizospheres and endospheres). Asterisks show significance at P<0.05 = ‘*’, P<0.01 = ‘**’, and P<0.001 = ‘***’ (see Table S14 for detail).

**Figure S23. Heatmap of differentially abundant nitrogen metabolism functions annotated at subsystem level 3.** Comparisons show the differences across plant compartments (bulk soils, rhizospheres and endospheres). Asterisks show significance at P<0.05 = ‘*’, P<0.01 = ‘**’, and P<0.001 = ‘***’ (see Table S14 for detail).

**Figure S24. Heatmap of differentially abundant phosphorus metabolism functions annotated at subsystem level 3.** Comparisons show the differences across plant compartments (bulk soils, rhizospheres and endospheres). Asterisks show significance at P<0.05 = ‘*’, P<0.01 = ‘**’, and P<0.001 = ‘***’ (see Table S14 for detail).

**Figure S25. Heatmap of differentially abundant secondary metabolism functions annotated at subsystem level 3.** Comparisons show the differences across plant compartments (bulk soils, rhizospheres and endospheres). Asterisks show significance at P<0.05 = ‘*’, P<0.01 = ‘**’, and P<0.001 = ‘***’ (see Table S14 for detail).

**Figure S26. Heatmap of differentially abundant regulation and cell signalling functions annotated at subsystem level 3.** Comparisons show the differences across plant compartments (bulk soils, rhizospheres and endospheres). Asterisks show significance at P<0.05 = ‘*’, P<0.01 = ‘**’, and P<0.001 = ‘***’ (see Table S14 for detail).

**Figure S27. Heatmap of differentially abundant stress response functions annotated at subsystem level 3.** Comparisons show the differences across plant compartments (bulk soils, rhizospheres and endospheres). Asterisks show significance at P<0.05 = ‘*’, P<0.01 = ‘**’, and P<0.001 = ‘***’ (see Table S14 for detail).

**Figure S28. Heatmap of differentially abundant motility and chemotaxis across aridity groups annotated at subsystem level 3.** Intercept differences compare the low aridity functions to a grand mean. Asterisks show significance at P<0.05 = ‘*’, P<0.01 = ‘**’, and P<0.001 = ‘***’ (see data accessibility statement for detailed statistical output).

**Figure S29. Heatmap of differentially abundant nitrogen metabolism functions across aridity groups annotated at subsystem level 3.** Intercept differences compare the low aridity functions to a grand mean. Asterisks show significance at P<0.05 = ‘*’, P<0.01 = ‘**’, and P<0.001 = ‘***’ (see data accessibility statement for detailed statistical output).

**Figure S30. Heatmap of differentially abundant phosphorus metabolism functions across aridity groups annotated at subsystem level 3.** Intercept differences compare the low aridity functions to a grand mean. Asterisks show significance at P<0.05 = ‘*’, P<0.01 = ‘**’, and P<0.001 = ‘***’ (see data accessibility statement for detailed statistical output).

**Figure S31. Heatmap of differentially abundant secondary metabolism functions across aridity groups annotated at subsystem level 3.** Intercept differences compare the low aridity functions to a grand mean. Asterisks show significance at P<0.05 = ‘*’, P<0.01 = ‘**’, and P<0.001 = ‘***’ (see data accessibility statement for detailed statistical output).

**Figure S32. Heatmap of differentially abundant regulation and cell signalling functions across aridity groups annotated at subsystem level 3.** Intercept differences compare the low aridity functions to a grand mean. Asterisks show significance at P<0.05 = ‘*’, P<0.01 = ‘**’, and P<0.001 = ‘***’ (see Data accessibility statement for detailed statistical output).

**Figure S33. Heatmap of differentially abundant stress response functions across aridity groups annotated at subsystem level 3.** Intercept differences compare the low aridity functions to a grand mean. Asterisks show significance at P<0.05 = ‘*’, P<0.01 = ‘**’, and P<0.001 = ‘***’ (see Data accessibility statement for detailed statistical output).

**Figure S34. Canonical correspondence analysis (CCA) on bacterial taxonomic community structure**. (a) Covariates with r < 0.75 were removed from CCA analyses. CCAs were constructed against environmental predictor variables in (b) bulk soil, (c) rhizosphere, and (d) endosphere communities. Sample points are coloured by sampling site.

**Figure S35. Canonical correspondence analysis on microbial functional community structure against environmental predictor variables in bulk soils.** Functional gene categories include (a) motility and chemotaxis, (b) stress response, (c) nitrogen metabolism, (d) phosphorus metabolism, (e) secondary metabolism, and (f) regulation and cell signalling**.** Coloured points represent samples belonging to each sampling site, black vectors indicate significant environmental variable associations.

**Figure S36. Canonical correspondence analysis on microbial functional community structure against environmental predictor variables in rhizospheres.** Functional gene categories include (a) motility and chemotaxis, (b) stress response, (c) nitrogen metabolism, (d) phosphorus metabolism, (e) secondary metabolism, and (f) regulation and cell signalling. Coloured points represent samples belonging to each sampling site, black vectors indicate significant environmental variable associations.

**Figure S37. Canonical correspondence analysis on microbial functional community structure against environmental predictor variables in endospheres.** Functional gene categories include (a) motility and chemotaxis, (b) stress response, (c) nitrogen metabolism, (d) phosphorus metabolism, (e) regulation and cell signalling**.** Coloured points represent samples belonging to each sampling site, black vectors indicate significant environmental variable associations.

**Table S1.** *T. triandra* sampling sites across southern Australia. This table includes sampling locations, aridity index data, and aridity classifications used during differential abundance and analysis with ANCOMBC. We also provide the number of successfully sequenced samples in each site and plant compartment included in our downstream analysis, and the number of samples included in each plant compartment (Soil, rhizosphere, endosphere) for each aridity category used to calculate out co-occurrence network analyses. Data was sourced from the Atlas of Living Australia (Belbin, 2011) using the following data layers: the mean annual aridity index (ALA, 2014), mean annual precipitation (CSIRO Ecology Services, 2010a), mean annual temperature (CSIRO Ecology Services, 2010b), and elevation above sea level (Commonwealth of Australia, 2009).

| **Site name** | **Latitude, longitude** | **Climate parameters** | | | |  | **Samples (n)** | | | **Sampling date** | **Aridity category** | **Number of samples (n) per aridity level** | | |
| --- | --- | --- | --- | --- | --- | --- | --- | --- | --- | --- | --- | --- | --- | --- |
|  |  | **Aridity index** | **Precipitation (mm)** | **Temperature (ºC)** | **Elevation (m)** |  | **Soil** | **Rhiz.** | **Endo.** |  |  | **Soil** | **Rhiz.** | **Endo.** |
| Mount Maria | -32.65862, 138.08985 | 0.3179 | 432 | 16.5 | 324 |  | 1 | 3 | 6 | 16/12/2021 | High | 7 | 5 | 11 |
| Barlunga Gap | -33.82, 138.17392 | 0.3469 | 392 | 16.4 | 147 |  | 6 | 2 | 5 | 14/12/2021 |  |  |  |  |
| Alligator Gorge | -32.71487, 138.10172 | 0.4450 | 531 | 15.3 | 489 |  | 4 | 2 | 5 | 15/12/2021 | Medium | 16 | 9 | 17 |
| Maitland | -34.37366, 137.71203 | 0.4532 | 460 | 16.3 | 190 |  | 6 | 4 | 6 | 21/12/2021 |  |  |  |  |
| Frahn’s Farm | -35.07231, 139.09781 | 0.4539 | 428 | 15.4 | 180 |  | 6 | 3 | 6 | 19/12/2021 |  |  |  |  |
| Sturt Gorge | -35.03311, 138.57324 | 0.6345 | 574 | 16.5 | 103 |  | 6 | 5 | 5 | 13/12/2021 | Low | 13 | 9 | 17 |
| Neagles Rock | -33.85031, 138.60674 | 0.6507 | 616 | 14.6 | 407 |  | 1 | 0 | 6 | 14/12/2021 |  |  |  |  |
| Scott Creek | -35.0872, 138.67266 | 0.9031 | 776 | 15.1 | 267 |  | 6 | 4 | 6 | 19/12/2021 |  |  |  |  |

**Table S2.** Upper and lower 95% confidence intervals for linear mixed-effect model estimates of the slope coefficient denoting a trend relationship between functional subsystems (level 1) of *Themeda triandra* soil/root microbiomes (i.e., see response variables listed) and mean annual aridity index, and the coefficient of determination (R^2^), following bootstrapping at 2000 permutations. Subsystem denotes the functional process at subsystem level 1: motility = motility and chemotaxis, stress = stress response, nitrogen = nitrogen metabolism, phosphorus = phosphorus metabolism, signal = regulation and cell signalling, and secondary metabolism. Compartment refers to whether these associations were tested with functions in *T. triandra* endospheres, rhizosphere, or bulk soils. Trend denotes the significant directional relationship in response to increasing aridity index (where high aridity index corresponds to increasingly wetter conditions, and low aridity index reflects drier conditions; i.e., a positive trend means the response variable increases with wetter conditions).

| **Response** | **Subsystem** | **Compartment** | **R^2^: Lower confidence interval** | **R^2^: Upper confidence interval** | **Estimate: Lower confidence interval** | **Estimate: Upper confidence interval** | **Trend** |
| --- | --- | --- | --- | --- | --- | --- | --- |
| Beta diversity ~  (Bray-Curtis distances) | motility | endosphere | 0.0000 | 0.0268 | -0.1137 | 0.035 | None |
|  | motility | rhizosphere | 0.0019 | 0.5947 | 0.0048 | 0.1568 | Positive |
|  | motility | soil | 0.0004 | 0.3187 | -0.1825 | 0.0221 | None |
|  | stress | endosphere | 0.0000 | 0.1435 | -0.1044 | 0.0107 | None |
|  | stress | rhizosphere | 0.0886 | 0.7645 | 0.0541 | 0.2494 | Positive |
|  | stress | soil | 0.0001 | 0.2397 | -0.1106 | 0.0366 | None |
|  | nitrogen | endosphere | 0.000 | 0.074 | -0.0676 | 0.0304 | None |
|  | nitrogen | rhizosphere | 0.0226 | 0.6326 | 0.0357 | 0.1975 | Positive |
|  | nitrogen | soil | 0.0000 | 0.1274 | -0.1044 | 0.0702 | None |
|  | phosphorus | endosphere | 0.0000 | 0.0754 | -0.0203 | 0.0454 | None |
|  | phosphorus | rhizosphere | 0.0616 | 0.6841 | 0.0394 | 0.1543 | Positive |
|  | phosphorus | soil | 0.0016 | 0.3217 | -0.1486 | -0.0013 | Negative |
|  | signal | endosphere | 0.0000 | 0.0906 | -0.1262 | 0.0244 | None |
|  | signal | rhizosphere | 0.0375 | 0.7237 | 0.0435 | 0.2314 | Positive |
|  | signal | soil | 0.0002 | 0.2976 | -0.1403 | 0.0111 |  |
|  | secondary metabolism | endosphere | 0.0000 | 0.0812 | -0.1247 | 0.0849 | None |
|  | secondary metabolism | rhizosphere | 0.0857 | 0.7320 | 0.0641 | 0.2989 | Positive |
|  | secondary metabolism | soil | 0.0015 | 0.3437 | -0.1611 | -0.0039 | Negative |
| Alpha diversity ~  (Richness of functions) | motility | endosphere | 0.0004 | 0.2936 | -7.55 | 76.04 | None |
|  | motility | rhizosphere | 0.0000 | 0.1980 | -34.07 | 73.07 | None |
|  | motility | soil | 0.0000 | 0.0171 | -89.77 | 54.368 | None |
|  | stress | endosphere | 0.0002 | 0.2939 | -48 | 266.6 | None |
|  | stress | rhizosphere | 0.0003 | 0.3262 | -36.9 | 365.3 | None |
|  | stress | soil | 0.0000 | 0.0590 | -355.3 | 193.46 | None |
|  | nitrogen | endosphere | 0.0001 | 0.2187 | -23.1 | 74.96 | None |
|  | nitrogen | rhizosphere | 0.0001 | 0.2683 | -26.32 | 96.88 | None |
|  | nitrogen | soil | 0.0000 | 0.0635 | -97.58 | 54.87 | None |
|  | phosphorus | endosphere | 0.0010 | 0.3479 | -1.47 | 60.46 | None |
|  | phosphorus | rhizosphere | 0.0001 | 0.2478 | -15.29 | 65.22 | None |
|  | phosphorus | soil | 0.0000 | 0.0285 | -59.403 | 38.683 | None |
|  | signal | endosphere | 0.0002 | 0.2858 | -33.25 | 184.55 | None |
|  | signal | rhizosphere | 0.0008 | 0.3168 | -8.4 | 262.3 | None |
|  | signal | soil | 0.0000 | 0.0402 | -14.39 | 26.77 | None |
|  | secondary metabolism | endosphere | 0.0000 | 0.0000 | -23.9613 | 17.9999 | None |
|  | secondary metabolism | rhizosphere | 0.0002 | 0.3272 | -2.79 | 52.21 | None |
|  | secondary metabolism | soil | 0.0001 | 0.1970 | -61.51 | 18.56 | None |
| Alpha diversity ~  (Effective no. functions) | motility | endosphere | 0.0000 | 0. 3322 | -13.187 | 6.722 | None |
|  | motility | rhizosphere | 0.0024 | 0.5325 | -30.82 | -1.36 | Negative |
|  | motility | soil | 0.0000 | 0.0912 | -7.532 | 9.931 | None |
|  | stress | endosphere | 0.0000 | 0.0022 | -18.333 | 25.395 | None |
|  | stress | rhizosphere | 0.0036 | 0.5750 | -88.04 | -1.14 | Negative |
|  | stress | soil | 0.0258 | 0.4823 | 14.28 | 63.76 | Positive |
|  | nitrogen | endosphere | 0.0052 | 0.2738 | 1.646 | 12.909 | Positive |
|  | nitrogen | rhizosphere | 0.0101 | 0.6302 | -25.79 | -2.81 | Negative |
|  | nitrogen | soil | 0.0000 | 0.0635 | -6.916 | 9.843 | None |
|  | phosphorus | endosphere | 0.0401 | 0.4046 | 3.225 | 9.818 | Positive |
|  | phosphorus | rhizosphere | 0.0018 | 0.5681 | -15.259 | 0.534 | None |
|  | phosphorus | soil | 0.0745 | 0.4982 | 3.661 | 11.959 | Positive |
|  | signal | endosphere | 0.0002 | 0.3477 | -22 | 0.08 | None |
|  | signal | rhizosphere | 0.0008 | 0.4691 | -41.71 | 3.78 | None |
|  | signal | soil | 0.0000 | 0.3084 | -211.14 | 130.3 | None |
|  | secondary metabolism | endosphere | 0.0000 | 0.0388 | -12.167 | 9.179 | None |
|  | secondary metabolism | rhizosphere | 0.0015 | 0.4358 | -0.282 | 6.03 | None |
|  | secondary metabolism | soil | 0.0438 | 0.6264 | 1.236 | 4.968 | Positive |
| Functional gene relative abundance (%) ~ | motility | endosphere | 0.0122 | 0.3092 | 0.1525 | 0.7833 | Positive |
|  | motility | rhizosphere | 0.0004 | 0.2945 | -0.7116 | 0.0611 | None |
|  | motility | soil | 0.0105 | 0.4205 | 0.1665 | 1.1414 | Positive |
|  | stress | endosphere | 0.0005 | 0.2292 | -0.2662 | -0.0076 | Negative |
|  | stress | rhizosphere | 0.0000 | 0.0006 | -0.3769 | 0.6685 | None |
|  | stress | soil | 0.0716 | 0.4520 | 0.665 | 1.97 | Positive |
|  | nitrogen | endosphere | 0.0000 | 0.1055 | -0.1795 | 0.0757 | None |
|  | nitrogen | rhizosphere | 0.0012 | 0.5083 | -0.4657 | 0.0436 | None |
|  | nitrogen | soil | 0.0001 | 0.3014 | -0.124 | 0.3465 | None |
|  | phosphorus | endosphere | 0.0001 | 0.2348 | -0.1369 | 0.0296 | None |
|  | phosphorus | rhizosphere | 0.0000 | 0.0279 | -0.2017 | 0.1573 | None |
|  | phosphorus | soil | 0.0000 | 0.0062 | -0.1209 | 0.1447 | None |
|  | signal | endosphere | 0.0000 | 0.0005 | -0.1899 | 0.1518 | None |
|  | signal | rhizosphere | 0.000 | 0.111 | -0.1227 | 0.3295 | None |
|  | signal | soil | 0.0392 | 0.4837 | 0.154 | 0.6785 | Positive |
|  | secondary metabolism | endosphere | 0.0000 | 0.0278 | -0.0564 | 0.1011 | None |
|  | secondary metabolism | rhizosphere | 0.0041 | 0.6476 | -0.1138 | -0.0102 | Negative |
|  | secondary metabolism | soil | 0.0017 | 0.3213 | -0.1121 | -0.0061 | Negative |

**Table S3.** Upper and lower 95% confidence intervals for linear mixed effect model estimates of the slope coefficient denoting a trend between *Themeda triandra* soil/root bacterial taxonomic beta diversity (Bray-Curtis distances), R^2^, and mean annual aridity index following bootstrapping at 2000 permutations. Compartment refers to whether these associations were tested in *T. triandra* endospheres, rhizospheres, or bulk soils. Trend denotes the directional relationship in response to increasing aridity index (where high aridity index corresponds to increasingly wetter conditions, and low aridity index reflects drier conditions, i.e., a positive trend means the response variable increases with wetter conditions).

| **Response** | **Compartment** | | **R2: Lower confidence interval** | **R2: Upper confidence interval** | **Estimate: Lower confidence interval** | **Estimate: Upper confidence interval** | **Trend** |
| --- | --- | --- | --- | --- | --- | --- | --- |
| Beta diversity ~  (Bray-Curtis distances) | Endosphere | 0.0454 | | 0.3003 | -0.1570 | -0.0375 | Negative |
|  | Rhizosphere | 0.0044 | | 0.6779 | 0.0472 | 0.3420 | Positive |
|  | Soil | 0.0000 | | 0.0714 | -0.1229 | 0.1252 | None |

**Table S4.** Explanatory variables included in canonical correspondence analysis (CCA) across *T. triandra* bulk soils, rhizospheres and endospheres.

| **Model variables** | | **Description** |
| --- | --- | --- |
|  | Aboveground biomass | Aboveground biomass of the host plant that each sample attributed to (bulk soil, rhizosphere or endosphere) |
|  | Aridity | Mean annual aridity index for each sample site, Atlas of Living Australia (Belbin 2011, ALA 2014) |
|  | Functional vegetation, | Relative abundance at each site for: graminoids, herbs, shrubs, canopy cover (trees), litter, and bare soil. |
|  | Latitude | Latitude coordinates for each sampling site |
|  | Longitude | Longitude coordinates for each sampling site |
|  | Physicochemical measurements (for bulk soil analyses only) | Ammonium nitrogen, nitrate, phosphorus, potassium, sulphur, organic carbon, electrical conductivity and pH (CaCl2) contained in the soil sampled at either 2 m or 30 cm from host plants |
|  | Density | Values pertaining to the density of *T. triandra* individuals for each sampling site |
|  | Trace elements and macronutrients (for rhizosphere analyses only) | Boron, calcium, copper, iron, magnesium, manganese, phosphorus, potassium, sodium, sulphur, total nitrogen, and zinc contained in the leaf tissue in sampled host plants |

**Table S5.** Sample sequencing information for taxonomic and functional annotation. Reads shows taxonomic and functional library sizes. The number of unique species and functions are also represented. All values reported are after data processing (i.e., removal of non-representative taxa, and quality control filtering).

| **Sample ID** | **Compartment** | **Site** | **Plant ID** | **Aridity index** | **Reads (taxonomy)** | **Unique Species (bacteria)** | **Reads (functions)** | **Unique functions** |
| --- | --- | --- | --- | --- | --- | --- | --- | --- |
| EA2 | endosphere | Sturt Gorge | A2 | 0.6345 | 148,881 | 7,546 | 168,228 | 11,712 |
| EA3 | endosphere | Sturt Gorge | A3 | 0.6345 | 197,097 | 7,800 | 228,762 | 12,622 |
| EA4 | endosphere | Sturt Gorge | A4 | 0.6345 | 176,143 | 7,958 | 205,948 | 12,399 |
| EA5 | endosphere | Sturt Gorge | A5 | 0.6345 | 1,295,071 | 9,157 | 1,339,565 | 18,345 |
| EA6 | endosphere | Sturt Gorge | A6 | 0.6345 | 1,544,200 | 9,061 | 1,603,085 | 18,441 |
| EB1 | endosphere | Barunga Gap | B1 | 0.3469 | 70,931 | 5,721 | 56,953 | 7,899 |
| EB2 | endosphere | Barunga Gap | B2 | 0.3469 | 264,316 | 7,959 | 274,509 | 12,865 |
| EB3 | endosphere | Barunga Gap | B3 | 0.3469 | 71,402 | 7,111 | 65,065 | 8,658 |
| EB5 | endosphere | Barunga Gap | B5 | 0.3469 | 384,699 | 8,086 | 401,163 | 14,189 |
| EB6 | endosphere | Barunga Gap | B6 | 0.3469 | 231,724 | 7,951 | 260,666 | 13,059 |
| EC1 | endosphere | Neagles Rock | C1 | 0.6507 | 22,793 | 5,744 | 26,975 | 6,580 |
| EC2 | endosphere | Neagles Rock | C2 | 0.6507 | 409,149 | 8,641 | 550,736 | 15,406 |
| EC3 | endosphere | Neagles Rock | C3 | 0.6507 | 98,305 | 7,325 | 117,101 | 10,836 |
| EC4 | endosphere | Neagles Rock | C4 | 0.6507 | 365,726 | 9,084 | 583,958 | 14,859 |
| ED2 | endosphere | Alligator Gorge | D2 | 0.4450 | 141,325 | 7,319 | 160,209 | 11,887 |
| ED3 | endosphere | Alligator Gorge | D3 | 0.4450 | 137,210 | 7,252 | 158,492 | 11,229 |
| ED4 | endosphere | Alligator Gorge | D4 | 0.4450 | 187,026 | 7,520 | 203,397 | 12,400 |
| ED5 | endosphere | Alligator Gorge | D5 | 0.4450 | 229,766 | 7,801 | 245,451 | 12,923 |
| ED6 | endosphere | Alligator Gorge | D6 | 0.4450 | 432,808 | 7,878 | 488,464 | 14,716 |
| EE1 | endosphere | Mount Maria | E1 | 0.3179 | 820,531 | 8,740 | 888,789 | 16,588 |
| EE2 | endosphere | Mount Maria | E2 | 0.3179 | 327,595 | 8,250 | 353,716 | 14,114 |
| EE3 | endosphere | Mount Maria | E3 | 0.3179 | 269,652 | 7,878 | 294,926 | 13,110 |
| EE4 | endosphere | Mount Maria | E4 | 0.3179 | 324,325 | 7,891 | 372,015 | 14,052 |
| EE5 | endosphere | Mount Maria | E5 | 0.3179 | 238,982 | 7,555 | 271,555 | 12,827 |
| EE6 | endosphere | Mount Maria | E6 | 0.3179 | 129,403 | 7,697 | 141,205 | 11,447 |
| EF1 | endosphere | Frahns Farm | F1 | 0.4539 | 234,588 | 7,727 | 277,724 | 13,685 |
| EF2 | endosphere | Frahns Farm | F2 | 0.4539 | 227,154 | 7,779 | 264,451 | 13,021 |
| EF3 | endosphere | Frahns Farm | F3 | 0.4539 | 353,919 | 8,040 | 408,296 | 14,239 |
| EF4 | endosphere | Frahns Farm | F4 | 0.4539 | 240,658 | 7,738 | 263,158 | 13,115 |
| EF5 | endosphere | Frahns Farm | F5 | 0.4539 | 227,168 | 7,773 | 263,060 | 13,215 |
| EF6 | endosphere | Frahns Farm | F6 | 0.4539 | 148,847 | 7,436 | 164,995 | 11,526 |
| EG1 | endosphere | Scott Creek | G1 | 0.9031 | 564,358 | 8,430 | 737,550 | 15,364 |
| EG2 | endosphere | Scott Creek | G2 | 0.9031 | 566,530 | 8,708 | 772,271 | 16,035 |
| EG3 | endosphere | Scott Creek | G3 | 0.9031 | 358,633 | 8,387 | 464,984 | 14,137 |
| EG4 | endosphere | Scott Creek | G4 | 0.9031 | 125,461 | 6,958 | 156,709 | 11,062 |
| EG5 | endosphere | Scott Creek | G5 | 0.9031 | 174,958 | 8,618 | 218,455 | 12,880 |
| EG6 | endosphere | Scott Creek | G6 | 0.9031 | 767,711 | 8,789 | 1,009,624 | 16,987 |
| EH1 | endosphere | Maitland | H1 | 0.4532 | 1,171,644 | 9,050 | 1,302,793 | 17,825 |
| EH2 | endosphere | Maitland | H2 | 0.4532 | 1,496,065 | 8,884 | 1,552,796 | 17,979 |
| EH3 | endosphere | Maitland | H3 | 0.4532 | 505,697 | 8,154 | 541,837 | 14,978 |
| EH4 | endosphere | Maitland | H4 | 0.4532 | 370,951 | 7,981 | 408,061 | 14,540 |
| EH5 | endosphere | Maitland | H5 | 0.4532 | 216,793 | 6,791 | 215,328 | 12,081 |
| EH6 | endosphere | Maitland | H6 | 0.4532 | 432,286 | 8,127 | 464,166 | 14,225 |
| RA1 | rhizosphere | Sturt Gorge | A1 | 0.6345 | 979,435 | 8,865 | 124,7696 | 17,327 |
| RA2 | rhizosphere | Sturt Gorge | A2 | 0.6345 | 196,556 | 8,039 | 273,390 | 12,459 |
| RA4 | rhizosphere | Sturt Gorge | A4 | 0.6345 | 169,331 | 8,467 | 328,812 | 12,010 |
| RA5 | rhizosphere | Sturt Gorge | A5 | 0.6345 | 195,745 | 8,610 | 365,039 | 12,368 |
| RA6 | rhizosphere | Sturt Gorge | A6 | 0.6345 | 186,873 | 8,595 | 336,609 | 11,891 |
| RB1 | rhizosphere | Barunga Gap | B1 | 0.3469 | 112,966 | 8,035 | 212,035 | 10,655 |
| RB2 | rhizosphere | Barunga Gap | B2 | 0.3469 | 183,869 | 8,551 | 349,185 | 12,025 |
| RD1 | rhizosphere | Alligator Gorge | D1 | 0.4450 | 228,563 | 8,647 | 443,737 | 13,329 |
| RD6 | rhizosphere | Alligator Gorge | D6 | 0.4450 | 145,898 | 8,539 | 305,006 | 11,544 |
| RE1 | rhizosphere | Mount Maria | E1 | 0.3179 | 172,850 | 8,563 | 356,732 | 12,279 |
| RE3 | rhizosphere | Mount Maria | E3 | 0.3179 | 110,858 | 8,410 | 237,807 | 10,897 |
| RE4 | rhizosphere | Mount Maria | E4 | 0.3179 | 398,165 | 8,626 | 623,623 | 15,223 |
| RF3 | rhizosphere | Frahns Farm | F3 | 0.4539 | 298,688 | 8,796 | 673,559 | 13,349 |
| RF4 | rhizosphere | Frahns Farm | F4 | 0.4539 | 198,734 | 8,595 | 382,951 | 13,157 |
| RF5 | rhizosphere | Frahns Farm | F5 | 0.4539 | 116,606 | 8,342 | 251,405 | 11,611 |
| RG3 | rhizosphere | Scott Creek | G3 | 0.9031 | 497,08 | 7,466 | 118,077 | 8,851 |
| RG4 | rhizosphere | Scott Creek | G4 | 0.9031 | 7,115 | 3,128 | 11,911 | 3,843 |
| RG5 | rhizosphere | Scott Creek | G5 | 0.9031 | 12,214 | 4,362 | 23,651 | 5,246 |
| RG6 | rhizosphere | Scott Creek | G6 | 0.9031 | NA | NA | 13,609 | 4,181 |
| RH1 | rhizosphere | Maitland | H1 | 0.4532 | 19,271 | 4,986 | 44,488 | 6,050 |
| RH3 | rhizosphere | Maitland | H3 | 0.4532 | 128,490 | 8,354 | 257,185 | 11,120 |
| RH4 | rhizosphere | Maitland | H4 | 0.4532 | 179,149 | 8,240 | 398,595 | 10,286 |
| RH6 | rhizosphere | Maitland | H6 | 0.4532 | 158,816 | 8,540 | 384,701 | 11,222 |
| SA1 | soil | Sturt Gorge | A1 | 0.6345 | 119,443 | 8,500 | 264,693 | 10,268 |
| SA2 | soil | Sturt Gorge | A2 | 0.6345 | 72,704 | 7,675 | 171,912 | 8,648 |
| SA3 | soil | Sturt Gorge | A3 | 0.6345 | 87,519 | 8,126 | 190,795 | 9,474 |
| SA4 | soil | Sturt Gorge | A4 | 0.6345 | 68,022 | 8,006 | 155,098 | 9,204 |
| SA5 | soil | Sturt Gorge | A5 | 0.6345 | 137,506 | 8,640 | 292,297 | 10,950 |
| SA6 | soil | Sturt Gorge | A6 | 0.6345 | 108,078 | 8,273 | 246,037 | 9,986 |
| SB1 | soil | Barunga Gap | B1 | 0.3469 | 90,468 | 7,741 | 277,375 | 8,903 |
| SB2 | soil | Barunga Gap | B2 | 0.3469 | 84,868 | 7,609 | 234,708 | 8,546 |
| SB3 | soil | Barunga Gap | B3 | 0.3469 | 78,110 | 7,670 | 186,826 | 8,710 |
| SB4 | soil | Barunga Gap | B4 | 0.3469 | 106,651 | 8,349 | 275,450 | 10,070 |
| SB5 | soil | Barunga Gap | B5 | 0.3469 | 95,049 | 8,100 | 255,235 | 9,552 |
| SB6 | soil | Barunga Gap | B6 | 0.3469 | 19,462 | 5,592 | 56,302 | 6,131 |
| SC1 | soil | Neagles Rock | C1 | 0.6507 | 74,654 | 8,043 | 171,753 | 9,334 |
| SC2 | soil | Neagles Rock | C2 | 0.6507 | 105,413 | 8,408 | 238,124 | 10,622 |
| SC3 | soil | Neagles Rock | C3 | 0.6507 | 133,725 | 7,414 | 279,538 | 9,167 |
| SC4 | soil | Neagles Rock | C4 | 0.6507 | 72,670 | 7,952 | 169,533 | 9,444 |
| SC5 | soil | Neagles Rock | C5 | 0.6507 | 117,833 | 8,450 | 275,391 | 10,572 |
| SC6 | soil | Neagles Rock | C6 | 0.6507 | 137,826 | 8657 | 315,805 | 11,432 |
| SD1 | soil | Alligator Gorge | D1 | 0.4450 | 118,696 | 8,533 | 291,863 | 11,174 |
| SD2 | soil | Alligator Gorge | D2 | 0.4450 | 146,304 | 8,709 | 380,494 | 11,880 |
| SD3 | soil | Alligator Gorge | D3 | 0.4450 | 113,813 | 8,570 | 288,153 | 10,961 |
| SD4 | soil | Alligator Gorge | D4 | 0.4450 | 166,267 | 8,671 | 354,156 | 12,263 |
| SE3 | soil | Mount Maria | E3 | 0.3179 | 60,442 | 6,726 | 151,270 | 8,240 |
| SF2 | soil | Frahns Farm | F2 | 0.4539 | 53,889 | 7,506 | 121,066 | 8,303 |
| SF3 | soil | Frahns Farm | F3 | 0.4539 | 125,891 | 8,696 | 297,857 | 11,428 |
| SF4 | soil | Frahns Farm | F4 | 0.4539 | 87,930 | 8,301 | 216,252 | 10,307 |
| SF5 | soil | Frahns Farm | F5 | 0.4539 | 87,996 | 8,325 | 196,589 | 10,059 |
| SG1 | soil | Scott Creek | G1 | 0.9031 | 61,711 | 6,388 | 111,736 | 7,574 |
| SG2 | soil | Scott Creek | G2 | 0.9031 | 95,039 | 8,465 | 268,302 | 10,368 |
| SG3 | soil | Scott Creek | G3 | 0.9031 | 110,860 | 8,474 | 308,153 | 11,551 |
| SG4 | soil | Scott Creek | G4 | 0.9031 | 641,625 | 9,342 | 1,530,255 | 15,849 |
| SG5 | soil | Scott Creek | G5 | 0.9031 | 629,842 | 9,368 | 1,417,307 | 15,966 |
| SG6 | soil | Scott Creek | G6 | 0.9031 | 963,770 | 9,289 | 1,976,684 | 16,507 |
| SH1 | soil | Maitland | H1 | 0.4532 | 175,366 | 8,800 | 421,075 | 12,147 |
| SH2 | soil | Maitland | H2 | 0.4532 | 115,035 | 8,513 | 271,665 | 11,007 |
| SH3 | soil | Maitland | H3 | 0.4532 | 107,227 | 7,534 | 264,682 | 9,185 |
| SH4 | soil | Maitland | H4 | 0.4532 | 129,221 | 8,148 | 343,810 | 10,307 |
| SH5 | soil | Maitland | H5 | 0.4532 | 109,460 | 8,397 | 275,408 | 10,378 |
| SH6 | soil | Maitland | H6 | 0.4532 | 156,345 | 8,731 | 389,924 | 11,421 |

**Table S6.** Mean relative abundance of reads, and standard deviations (SD) attributed to six isolated functional categories at SEED subsystem level 1 across the bulk soils, rhizospheres, and endospheres of *T. triandra* plants.

| **Subsystem 1** | **Compartment** | **Mean relative abundance (%)** | **SD relative abundance (%)** |
| --- | --- | --- | --- |
| Motility and Chemotaxis | endosphere | 1.438 | 0.224 |
| Motility and Chemotaxis | rhizosphere | 1.375 | 0.263 |
| Motility and Chemotaxis | soil | 1.052 | 0.314 |
| Nitrogen Metabolism | endosphere | 1.235 | 0.084 |
| Nitrogen Metabolism | rhizosphere | 0.9 | 0.137 |
| Nitrogen Metabolism | soil | 0.677 | 0.122 |
| Phosphorus Metabolism | endosphere | 1.221 | 0.05 |
| Phosphorus Metabolism | rhizosphere | 1.302 | 0.095 |
| Phosphorus Metabolism | soil | 1.304 | 0.081 |
| Regulation and Cell signalling | endosphere | 1.99 | 0.081 |
| Regulation and Cell signalling | rhizosphere | 1.821 | 0.121 |
| Regulation and Cell signalling | soil | 1.655 | 0.155 |
| Secondary Metabolism | endosphere | 0.228 | 0.051 |
| Secondary Metabolism | rhizosphere | 0.207 | 0.029 |
| Secondary Metabolism | soil | 0.209 | 0.035 |
| Stress Response | endosphere | 4.188 | 0.092 |
| Stress Response | rhizosphere | 4.086 | 0.291 |
| Stress Response | soil | 3.67 | 0.469 |

**Table S7.** Full statistical output for taxonomic alpha diversity linear mixed effects models and subsequent pairwise comparisons.

| **Type** | **Test** | **Full model** | **Response variable** | **Predictor** | **Random Effects** | **Df** | **Test statistic type** | **Test statistic value** | **P-value** | **Significance** |
| --- | --- | --- | --- | --- | --- | --- | --- | --- | --- | --- |
| taxonomy alpha diversity | LMEM | Bacterial Richness ~ site + compartment + (1\|plant_id) | Bacterial Richness | site | Plant_ID | 7 | χ2 | 11.3733 | 0.1231 | NS |
|  |  |  |  | compartment | Plant_ID | 2 | χ2 | 2.5627 | 0.2777 | NS |
| taxonomy alpha diversity | LMEM | Bacterial richness ~ compartment + (1\|plant_id) | Bacterial Richness | compartment | Plant_ID | 2 | χ2 | 2.9945 | 0.2237 | NS |
| taxonomy alpha diversity | LMEM | Shannon diversity ~ site + compartment + (1\|plant_id) | Shannon's diveristy | site | Plant_ID | 7 | χ2 | 19.999 | 0.005572 | ** |
|  | Tukey | site pairwise comparisons |  | Barunga Gap - Alligator Gorge |  | - | z value | -2.993 | 0.0549 | NS |
|  |  |  |  | Frahns Farm - Alligator Gorge |  | - |  | 0.133 | 1 | NS |
|  |  |  |  | Maitland - Alligator Gorge |  | - |  | -2.077 | 0.4273 | NS |
|  |  |  |  | Mount Maria - Alligator Gorge |  | - |  | -0.57 | 0.9992 | NS |
|  |  |  |  | Neagles Rock - Alligator Gorge |  | - |  | 0.103 | 1 | NS |
|  |  |  |  | Scott Creek - Alligator Gorge |  | - |  | -1.681 | 0.6978 | NS |
|  |  |  |  | Sturt Gorge - Alligator Gorge |  | - |  | -1.878 | 0.5641 | NS |
|  |  |  |  | Frahns Farm - Barunga Gap |  | - |  | 3.258 | 0.0247 | * |
|  |  |  |  | Maitland - Barunga Gap |  | - |  | 1.104 | 0.9556 | NS |
|  |  |  |  | Mount Maria - Barunga Gap |  | - |  | 2.285 | 0.3 | NS |
|  |  |  |  | Neagles Rock - Barunga Gap |  | - |  | 3.013 | 0.0525 | NS |
|  |  |  |  | Scott Creek - Barunga Gap |  | - |  | 1.477 | 0.8184 | NS |
|  |  |  |  | Sturt Gorge - Barunga Gap |  | - |  | 1.303 | 0.8971 | NS |
|  |  |  |  | Maitland - Frahns Farm |  | - |  | -2.325 | 0.278 | NS |
|  |  |  |  | Mount Maria - Frahns Farm |  | - |  | -0.723 | 0.9963 | NS |
|  |  |  |  | Neagles Rock - Frahns Farm |  | - |  | -0.022 | 1 | NS |
|  |  |  |  | Scott Creek - Frahns Farm |  | - |  | -1.903 | 0.5461 | NS |
|  |  |  |  | Sturt Gorge - Frahns Farm |  | - |  | -2.117 | 0.4013 | NS |
|  |  |  |  | Mount Maria - Maitland |  | - |  | 1.383 | 0.8642 | NS |
|  |  |  |  | Neagles Rock - Maitland |  | - |  | 2.104 | 0.4095 | NS |
|  |  |  |  | Scott Creek - Maitland |  | - |  | 0.408 | 0.9999 | NS |
|  |  |  |  | Sturt Gorge - Maitland |  | - |  | 0.214 | 1 | NS |
|  |  |  |  | Neagles Rock - Mount Maria |  | - |  | 0.644 | 0.9982 | NS |
|  |  |  |  | Scott Creek - Mount Maria |  | - |  | -1.008 | 0.9731 | NS |
|  |  |  |  | Sturt Gorge - Mount Maria |  | - |  | -1.195 | 0.9331 | NS |
|  |  |  |  | Scott Creek - Neagles Rock |  | - |  | -1.731 | 0.665 | NS |
|  |  |  |  | Sturt Gorge - Neagles Rock |  | - |  | -1.908 | 0.5431 | NS |
|  |  |  |  | Sturt Gorge - Scott Creek |  | - |  | -0.197 | 1 | NS |
|  | LMEM |  |  | compartment | Plant_ID | 2 |  | 92.283 | 2.20E-16 | *** |
|  | Tukey | compartment pairwise comparisons |  | rhizosphere - soil |  | - | z value | -2.529 | 0.0304 | * |
|  |  |  |  | endosphere - soil |  | - |  | -9.41 | <0.001 | *** |
|  |  |  |  | rhizosphere - endosphere |  | - |  | -5.379 | <0.001 | *** |
| taxonomy alpha diversity | LMEM | Shannon ~ compartment + (1\|plant_id) | Shannon's diveristy | compartment | Plant_ID | 2 | χ2 | 80.453 | <0.001 | *** |
|  | Tukey | compartment pairwise comparisons |  | rhizosphere - soil |  | - | z value | -2.551 | 0.0286 | * |
|  |  |  |  | endosphere - soil |  | - |  | -8.854 | <0.001 | *** |
|  |  |  |  | endosphere - rhizosphere |  | - |  | -4.858 | <0.001 | *** |
| taxonomy alpha diversity | LMEM | effective no species ~ site + compartment + (1\|plant_id) | Effective no. species | site | Plant_ID | 7 | χ2 | 22.448 | 0.002126 | ** |
|  | Tukey | site pairwise comparisons |  | Barunga Gap - Alligator Gorge |  | - | z value | -3.2 | 0.0294 | * |
|  |  |  |  | Frahns Farm - Alligator Gorge |  | - |  | -0.027 | 1 | NS |
|  |  |  |  | Maitland - Alligator Gorge |  | - |  | -2.436 | 0.2215 | NS |
|  |  |  |  | Mount Maria - Alligator Gorge |  | - |  | -0.978 | 0.9773 | NS |
|  |  |  |  | Neagles Rock - Alligator Gorge |  | - |  | -0.319 | 1 | NS |
|  |  |  |  | Scott Creek - Alligator Gorge |  | - |  | -1.865 | 0.5731 | NS |
|  |  |  |  | Sturt Gorge - Alligator Gorge |  | - |  | -2.62 | 0.1468 | NS |
|  |  |  |  | Frahns Farm - Barunga Gap |  | - |  | 3.307 | 0.0211 | * |
|  |  |  |  | Maitland - Barunga Gap |  | - |  | 0.955 | 0.9803 | NS |
|  |  |  |  | Mount Maria - Barunga Gap |  | - |  | 2.063 | 0.4371 | NS |
|  |  |  |  | Neagles Rock - Barunga Gap |  | - |  | 2.773 | 0.1009 | NS |
|  |  |  |  | Scott Creek - Barunga Gap |  | - |  | 1.508 | 0.802 | NS |
|  |  |  |  | Sturt Gorge - Barunga Gap |  | - |  | 0.751 | 0.9953 | NS |
|  |  |  |  | Maitland - Frahns Farm |  | - |  | -2.526 | 0.1833 | NS |
|  |  |  |  | Mount Maria - Frahns Farm |  | - |  | -0.993 | 0.9753 | NS |
|  |  |  |  | Neagles Rock - Frahns Farm |  | - |  | -0.304 | 1 | NS |
|  |  |  |  | Scott Creek - Frahns Farm |  | - |  | -1.923 | 0.5319 | NS |
|  |  |  |  | Sturt Gorge - Frahns Farm |  | - |  | -2.721 | 0.1151 | NS |
|  |  |  |  | Mount Maria - Maitland |  | - |  | 1.287 | 0.903 | NS |
|  |  |  |  | Neagles Rock - Maitland |  | - |  | 1.994 | 0.4843 | NS |
|  |  |  |  | Scott Creek - Maitland |  | - |  | 0.597 | 0.9989 | NS |
|  |  |  |  | Sturt Gorge - Maitland |  | - |  | -0.213 | 1 | NS |
|  |  |  |  | Neagles Rock - Mount Maria |  | - |  | 0.63 | 0.9985 | NS |
|  |  |  |  | Scott Creek - Mount Maria |  | - |  | -0.75 | 0.9953 | NS |
|  |  |  |  | Sturt Gorge - Mount Maria |  | - |  | -1.469 | 0.8226 | NS |
|  |  |  |  | Scott Creek - Neagles Rock |  | - |  | -1.457 | 0.8292 | NS |
|  |  |  |  | Sturt Gorge - Neagles Rock |  | - |  | -2.166 | 0.3701 | NS |
|  |  |  |  | Sturt Gorge - Scott Creek |  | - |  | -0.804 | 0.9929 | NS |
|  | LMEM |  | Effective no. species | compartment | Plant_ID | 2 | χ2 | 109.074 | <0.001 | *** |
|  | Tukey | compartment pairwise comparisons |  | rhizosphere - endosphere |  | - | z value | 5.49 | <0.001 | *** |
|  |  |  |  | soil - endosphere |  | - |  | 10.308 | <0.001 | *** |
|  |  |  |  | soil - rhizosphere |  | - |  | 3.157 | 0.00455 | ** |
| taxonomy alpha diversity | LMEM | effective no species ~ compartment + (1\|plant_id) | Effective no. species | compartment | Plant_ID | 2 | χ2 | 95.27 | <0.001 | *** |
|  | Tukey | compartment pairwise comparisons |  | rhizosphere - endosphere |  | - | z value | 4.884 | <0.001 | *** |
|  |  |  |  | soil - endosphere |  | - |  | 9.698 | <0.001 | *** |
|  |  |  |  | soil - rhizosphere |  | - |  | 3.206 | 0.00368 | ** |
| taxonomy alpha diversity | LMEM | Pielou's evenness ~ site + compartment + (1\|plant_id) | Pielou's evenness index | site | Plant_ID | 7 | χ2 | 12.744 | 0.0786 | NS |
|  |  |  |  | compartment | Plant_ID | 2 | χ2 | 73.731 | <0.001 | *** |
|  | Tukey | compartment pairwise comparisons |  | rhizosphere - endosphere |  | - | z value | 5.056 | <0.001 | *** |
|  |  |  |  | soil - endosphere |  | - |  | 8.345 | <0.001 | *** |
|  |  |  |  | soil - rhizosphere |  | - |  | 1.966 | 0.12 |  |
| taxonomy alpha diversity | LMEM | Pielou's evenness ~ site + compartment + (1\|plant_id) | Pielou's evenness index | compartment | Plant_ID | 2 | χ2 | 68.538 | <0.001 | *** |
|  | Tukey | compartment pairwise comparisons |  | rhizosphere - endosphere |  | - | z value | 4.784 | <0.001 | *** |
|  |  |  |  | soil - endosphere |  | - |  | 8.108 | <0.001 | *** |
|  |  |  |  | soil - rhizosphere |  | - |  | 2.022 | 0.106 | NS |
| taxonomy alpha diversity | LMEM | Bacterial richness ~ Aridity index + (1\|plant_id) | Bacterial Richness | Aridity index | Plant_ID | 1 | χ2 | 1.667 | 0.1967 | NS |
| taxonomy alpha diversity | LMEM | Shannons diversity ~ Aridity index + (1\|plant_id) | Shannon's diveristy | Aridity index | Plant_ID | 1 | χ2 | 0.2774 | 0.5984 | NS |
| taxonomy alpha diversity | LMEM | Pielou's evenness~ Aridity index + (1\|plant_id) | Pielou's evenness | Aridity index | Plant_ID | 1 | χ2 | 0.2448 | 0.6207 | NS |

**Table S8.** Beta diversity analysis output showing the effects of plant compartment (bulk soil, rhizosphere and endosphere) on bacterial community compositions using PERMANOVAS via the *adonis2* function in the R package Vegan.

| **Response variable** | **Statistical test** | **Predictor variable** | **Degrees of freedom** | **F-statistic** | **R squared value** | **P value** |
| --- | --- | --- | --- | --- | --- | --- |
| Bacterial taxonomy | PERMANOVA | ~Compartment | 2 and 101 | 55.96 | 0.53 | <0.001*** |
| Bacterial taxonomy | PERMANOVA | ~Aridity index | 1 and 102 | 3.53 | 0.03 | 0.027* |
| Bacterial taxonomy | PERMANOVA | ~Sampling site | 7 and 96 | 2.73 | 0.17 | 0.002** |
| Microbial functions | PERMANOVA | ~Compartment | 2 and 102 | 45.73 | 0.47 | <0.001*** |
| Microbial functions | PERMANOVA | ~Aridity index | 2 and 102 | 4.61 | 0.04 | 0.008** |

**Table S9.** Full statistical output for distance to centroid estimates for taxonomic and functional beta diversity

| **Response variable** | **Statistical test** | **Permutations** | **Predictor variable** | **Degrees of freedom** | **F-statistic** | **P value** |
| --- | --- | --- | --- | --- | --- | --- |
| Distance to centroid - taxonomic beta diversity~ | Permutation test for homogeneity of multivariate dispersions | 999 | Compartment | 2 and 102 | 4.7072 | 0.015 |
|  | Pairwise compartment test |  | Rhizosphere-Endosphere |  |  | 0.0090047 |
|  | Pairwise compartment test |  | Soil-Endosphere |  |  | 0.7666314 |
|  | Pairwise compartment test |  | Soil-Rhizosphere |  |  | 0.0488275 |
| Distance to centroid - functional beta diversity~ | Permutation test for homogeneity of multivariate dispersions | 999 | Compartment | 2 and 102 | 14.647 | <0.001 |
|  | Pairwise compartment test |  | Rhizosphere-Endosphere |  |  | 0.0004887 |
|  | Pairwise compartment test |  | Soil-Endosphere |  |  | 0.0000068 |
|  | Pairwise compartment test |  | Soil-Rhizosphere |  |  | 0.9255204 |

**Table S10.** Full statistical output for functional alpha diversity linear mixed effects models and subsequent pairwise comparisons

| **Type** | **Test** | **Full model** | **Response variable** | **predictor** | **Random Effects** | **df** | **Test statistic type** | **Test statistic value** | **P-value** | **Significance** |
| --- | --- | --- | --- | --- | --- | --- | --- | --- | --- | --- |
| functional alpha diversity | LMEM | Functional richness ~ site + compartment + (1\|plant_id) | Functional richness | site | Plant_ID | 7 | χ2 | 2.6844 | 0.912583 | NS |
|  |  |  | Functional richness | compartment | Plant_ID | 2 | χ2 | 10.0471 | 0.006581 | ** |
|  | Tukey | compartment pairwise comparisons |  | rhizosphere - endosphere |  | - | z value | 2.887 | 0.0107 | * |
|  |  |  |  | soil - endosphere |  | - |  | 2.328 | 0.0515 | NS |
|  |  |  |  | soil - rhizosphere |  | - |  | -0.865 | 0.661 | NS |
| functional alpha diversity | LMEM | Functional richness ~ compartment + (1\|plant_id) | Functional richness | compartment | Plant_ID | 2 | χ2 | 10.3 | 0.005799 | ** |
|  | Tukey | compartment pairwise comparisons |  | rhizosphere - endosphere |  | - | z value | 2.84 | 0.0124 | * |
|  |  |  |  | soil - endosphere |  | - |  | 2.519 | 0.0315 | * |
|  |  |  |  | soil - rhizosphere |  | - |  | -0.676 | 0.7765 | NS |
| functional alpha diversity | LMEM | Shannon's diversity ~ site + compartment + (1\|plant_id) | Shannon's diversity | site | Plant_ID | 7 | χ2 | 30.992 | <0.001 | *** |
|  | Tukey | site pairwise comparisons |  | Barunga Gap - Alligator Gorge |  | - | z value | -4.372 | <0.001 | *** |
|  |  |  |  | Frahns Farm - Alligator Gorge |  | - |  | -0.264 | 1 | NS |
|  |  |  |  | Maitland - Alligator Gorge |  | - |  | -2.465 | 0.2095 | NS |
|  |  |  |  | Mount Maria - Alligator Gorge |  | - |  | -0.927 | 0.9833 | NS |
|  |  |  |  | Neagles Rock - Alligator Gorge |  | - |  | -1.583 | 0.7591 | NS |
|  |  |  |  | Scott Creek - Alligator Gorge |  | - |  | -2.028 | 0.4605 | NS |
|  |  |  |  | Sturt Gorge - Alligator Gorge |  | - |  | -0.498 | 0.9997 | NS |
|  |  |  |  | Frahns Farm - Barunga Gap |  | - |  | 4.281 | <0.001 | *** |
|  |  |  |  | Maitland - Barunga Gap |  | - |  | 2.209 | 0.3439 | NS |
|  |  |  |  | Mount Maria - Barunga Gap |  | - |  | 3.242 | 0.0255 | * |
|  |  |  |  | Neagles Rock - Barunga Gap |  | - |  | 2.595 | 0.1565 | NS |
|  |  |  |  | Scott Creek - Barunga Gap |  | - |  | 2.667 | 0.1315 | NS |
|  |  |  |  | Sturt Gorge - Barunga Gap |  | - |  | 4.256 | <0.001 | *** |
|  |  |  |  | Maitland - Frahns Farm |  | - |  | -2.296 | 0.2938 | NS |
|  |  |  |  | Mount Maria - Frahns Farm |  | - |  | -0.709 | 0.9967 | NS |
|  |  |  |  | Neagles Rock - Frahns Farm |  | - |  | -1.381 | 0.865 | NS |
|  |  |  |  | Scott Creek - Frahns Farm |  | - |  | -1.837 | 0.592 | NS |
|  |  |  |  | Sturt Gorge - Frahns Farm |  | - |  | -0.233 | 1 | NS |
|  |  |  |  | Mount Maria - Maitland |  | - |  | 1.371 | 0.8695 | NS |
|  |  |  |  | Neagles Rock - Maitland |  | - |  | 0.66 | 0.9979 | NS |
|  |  |  |  | Scott Creek - Maitland |  | - |  | 0.485 | 0.9997 | NS |
|  |  |  |  | Sturt Gorge - Maitland |  | - |  | 2.178 | 0.3625 | NS |
|  |  |  |  | Neagles Rock - Mount Maria |  | - |  | -0.627 | 0.9985 | NS |
|  |  |  |  | Scott Creek - Mount Maria |  | - |  | -0.95 | 0.9808 | NS |
|  |  |  |  | Sturt Gorge - Mount Maria |  | - |  | 0.521 | 0.9996 | NS |
|  |  |  |  | Scott Creek - Neagles Rock |  | - |  | -0.24 | 1 | NS |
|  |  |  |  | Sturt Gorge - Neagles Rock |  | - |  | 1.221 | 0.9251 | NS |
|  |  |  |  | Sturt Gorge - Scott Creek |  | - |  | 1.694 | 0.6894 | NS |
|  | LMEM |  | Shannon's diversity | compartment | Plant_ID | 2 | χ2 | 182.969 | <0.001 | *** |
|  | Tukey | compartment pairwise comparisons |  | rhizosphere - endosphere |  | - | z value | -8.463 | <0.001 | *** |
|  |  |  |  | soil - endosphere |  | - |  | -13.013 | <0.001 | *** |
|  |  |  |  | soil - rhizosphere |  | - |  | -2.663 | 0.0207 | * |
| functional alpha diversity | LMEM | Shannon's diversity ~ compartment + (1\|plant_id) | Shannon's diversity | compartment | Plant_ID | 2 | χ2 | 159.95 | <0.001 | *** |
|  | Tukey |  |  | rhizosphere - endosphere |  | - | z value | -7.576 | <0.001 | *** |
|  |  |  |  | soil - endosphere |  | - |  | -12.334 | <0.001 | *** |
|  |  |  |  | soil - rhizosphere |  | - |  | -2.93 | 0.00959 | ** |
| functional alpha diversity | LMEM | Effective no. functions ~ site + compartment + (1\|plant_id) | Effective no. functions | site | Plant_ID | 7 | χ2 | 31.449 | <0.001 | *** |
|  | TUkey | site pairwise comparisons |  | Barunga Gap - Alligator Gorge |  | - | z value | -4.376 | <0.001 | *** |
|  |  |  |  | Frahns Farm - Alligator Gorge |  | - |  | -0.282 | 1 | NS |
|  |  |  |  | Maitland - Alligator Gorge |  | - |  | -2.548 | 0.1741 | NS |
|  |  |  |  | Mount Maria - Alligator Gorge |  | - |  | -0.845 | 0.9903 | NS |
|  |  |  |  | Neagles Rock - Alligator Gorge |  | - |  | -1.772 | 0.6368 | NS |
|  |  |  |  | Scott Creek - Alligator Gorge |  | - |  | -1.976 | 0.4959 | NS |
|  |  |  |  | Sturt Gorge - Alligator Gorge |  | - |  | -0.502 | 0.9997 | NS |
|  |  |  |  | Frahns Farm - Barunga Gap |  | - |  | 4.267 | <0.001 | *** |
|  |  |  |  | Maitland - Barunga Gap |  | - |  | 2.126 | 0.3953 | NS |
|  |  |  |  | Mount Maria - Barunga Gap |  | - |  | 3.329 | 0.0196 | * |
|  |  |  |  | Neagles Rock - Barunga Gap |  | - |  | 2.401 | 0.2389 | NS |
|  |  |  |  | Scott Creek - Barunga Gap |  | - |  | 2.726 | 0.1136 | NS |
|  |  |  |  | Sturt Gorge - Barunga Gap |  | - |  | 4.257 | <0.001 | *** |
|  |  |  |  | Maitland - Frahns Farm |  | - |  | -2.364 | 0.258 | NS |
|  |  |  |  | Mount Maria - Frahns Farm |  | - |  | -0.607 | 0.9988 | NS |
|  |  |  |  | Neagles Rock - Frahns Farm |  | - |  | -1.559 | 0.7729 | NS |
|  |  |  |  | Scott Creek - Frahns Farm |  | - |  | -1.764 | 0.6427 | NS |
|  |  |  |  | Sturt Gorge - Frahns Farm |  | - |  | -0.218 | 1 | NS |
|  |  |  |  | Mount Maria - Maitland |  | - |  | 1.539 | 0.7845 | NS |
|  |  |  |  | Neagles Rock - Maitland |  | - |  | 0.536 | 0.9995 | NS |
|  |  |  |  | Scott Creek - Maitland |  | - |  | 0.635 | 0.9984 | NS |
|  |  |  |  | Sturt Gorge - Maitland |  | - |  | 2.266 | 0.3105 | NS |
|  |  |  |  | Neagles Rock - Mount Maria |  | - |  | -0.887 | 0.9872 | NS |
|  |  |  |  | Scott Creek - Mount Maria |  | - |  | -0.989 | 0.9759 | NS |
|  |  |  |  | Sturt Gorge - Mount Maria |  | - |  | 0.429 | 0.9999 | NS |
|  |  |  |  | Scott Creek - Neagles Rock |  | - |  | 0.014 | 1 | NS |
|  |  |  |  | Sturt Gorge - Neagles Rock |  | - |  | 1.421 | 0.8467 | NS |
|  |  |  |  | Sturt Gorge - Scott Creek |  | - |  | 1.632 | 0.7287 | NS |
|  |  |  | Effective no. functions | compartment | Plant_ID | 2 | χ2 | 218.189 | <0.001 | *** |
|  |  | compartment pairwise comparisons |  | rhizosphere - endosphere |  | - | z value | -9.664 | <0.001 | *** |
|  |  |  |  | soil - endosphere |  | - |  | -14.64 | <0.001 | *** |
|  |  |  |  | soil - rhizosphere |  | - |  | -2.858 | 0.0118 | * |
| functional alpha diversity | LMEM | Effective no. functions ~ compartment + (1\|plant_id) | Effective no. functions | compartment | Plant_ID | 2 | χ2 | 202.35 | <0.001 | *** |
|  |  |  |  |  |  | - | z value | -8.632 | <0.001 | *** |
|  |  |  |  |  |  | - |  | -13.841 | <0.001 | *** |
|  |  |  |  |  |  | - |  | -3.16 | 0.00455 | ** |
| functional alpha diversity | LMEM | Pielou's evenness ~ site + compartment + (1\|plant_id) | Pielou's evenness | site | Plant_ID | 7 | χ2 | 4.0765 | 0.7709 | NS |
|  |  |  |  | compartment | Plant_ID | 2 | χ2 | 40.3641 | <0.001 | *** |
|  |  | compartment pairwise comparisons |  | rhizosphere - endosphere |  | - | z value | -4.844 | <0.001 | *** |
|  |  |  |  | soil - endosphere |  | - |  | -5.672 | <0.001 | *** |
|  |  |  |  | soil - rhizosphere |  | - |  | -0.045 | 0.999 | NS |
| functional alpha diversity | LMEM | Pielou's evenness ~ compartment + (1\|plant_id) | Pielou's evenness | compartment | Plant_ID | 2 | χ2 | 159.95 | <0.001 | *** |
|  |  | compartment pairwise comparisons |  | rhizosphere - endosphere |  | - | z value | -7.576 | <0.001 | *** |
|  |  |  |  | soil - endosphere |  | - |  | 12.334 | <0.001 | *** |
|  |  |  |  | soil - rhizosphere |  | - |  | -2.93 | 0.00928 | ** |
| functional alpha diversity | LMEM | Functional richness ~ Aridity index + (1\|plant_id) | Functional Richness | Aridity index | Plant_ID | 1 | χ2 | 1.367 | 0.2423 | NS |
| functional alpha diversity | LMEM | Shannons diversity ~ Aridity index + (1\|plant_id) | Shannon's diveristy | Aridity index | Plant_ID | 1 | χ2 | 0.0817 | 0.775 | NS |
| functional alpha diversity | LMEM | Effective no. functions ~ Aridity index + (1\|plant_id) | Effective no. functions | Aridity index | Plant_ID | 1 | χ2 | 0.1165 | 0.7328 | NS |
| functional alpha diversity | LMEM | Pielou's evenness~ Aridity index + (1\|plant_id) | Pielou's evenness | Aridity index | Plant_ID | 1 | χ2 | 0.4785 | 0.4891 | NS |

**Table S11.** Statistical output for linear mixed effects model (LMEM) comparing bacterial alpha diversity by functional alpha diversity.

| **Test** | **Full model** | **Response variable** | **Predictor variable** | **Random Effects** | **df** | **Test statistic type** | **Test statistic value** | **P-value** | **Significance** |
| --- | --- | --- | --- | --- | --- | --- | --- | --- | --- |
| LMEM | Effective no. functions ~ Effective no. species + compartment + (1\|compartment) | Effective no. functions | Effective no. species | Compartment | 1 | χ2 | 29.0432 | <0.001 | *** |
|  |  |  | Compartment | Compartment | 2 | χ2 | 5.3444 | 0.0691 | NS |

**Table S12**. Statistical output for distance to centroid estimates for functional genes at subsystem level 1.

| **Response variable** | **Statistical test** | **Permutations** | **Predictor variable** | **Degrees of freedom** | **F-statistic** | **P value** |
| --- | --- | --- | --- | --- | --- | --- |
| Motility and chemotaxis~ | Permutation test for homogeneity of multivariate dispersions | 999 | Compartment | 2 and 102 | 8.9856 | 0.002 |
|  | Pairwise compartment test - Rhizosphere-Endosphere |  |  |  |  | 0.001 |
|  | Pairwise compartment test - Soil-Endosphere |  |  |  |  | 0.003 |
|  | Pairwise compartment test - Soil-Rhizosphere |  |  |  |  | 0.716 |
| Phosphorus metabolism~ | Permutation test for homogeneity of multivariate dispersions | 999 | Compartment | 2 and 102 | 8.8053 | 0.002 |
|  | Pairwise compartment test - Rhizosphere-Endosphere |  |  |  |  | 0.0087402 |
|  | Pairwise compartment test - Soil-Endosphere |  |  |  |  | 0.0005252 |
|  | Pairwise compartment test - Soil-Rhizosphere |  |  |  |  | 0.9526302 |
| Nitrogen metabolism~ | Permutation test for homogeneity of multivariate dispersions | 999 | Compartment | 2 and 102 | 0.001 | 0.002 |
|  | Pairwise compartment test - Rhizosphere-Endosphere |  |  |  |  | 0.0000039 |
|  | Pairwise compartment test - Soil-Endosphere |  |  |  |  | 0.0000002 |
|  | Pairwise compartment test - Soil-Rhizosphere |  |  |  |  | 0.9874346 |
| Regulation and cell signalling~ | Permutation test for homogeneity of multivariate dispersions | 999 | Compartment | 2 and 102 | 8.5343 | 0.001 |
|  | Pairwise compartment test - Rhizosphere-Endosphere |  |  |  |  | 0.0008591 |
|  | Pairwise compartment test - Soil-Endosphere |  |  |  |  | 0.00671 |
|  | Pairwise compartment test - Soil-Rhizosphere |  |  |  |  | 0.5392789 |
| Secondary metabolism~ | Permutation test for homogeneity of multivariate dispersions | 999 | Compartment | 2 and 102 | 7.3946 | 0.002 |
|  | Pairwise compartment test - Rhizosphere-Endosphere |  |  |  |  | 0.1191559 |
|  | Pairwise compartment test - Soil-Endosphere |  |  |  |  | 0.0006778 |
|  | Pairwise compartment test - Soil-Rhizosphere |  |  |  |  | 0.4261641 |
| Stress response~ | Permutation test for homogeneity of multivariate dispersions | 999 | Compartment | 2 and 102 | 11.477 | <0.001 |
|  | Pairwise compartment test - Rhizosphere-Endosphere |  |  |  |  | 0.0010103 |
|  | Pairwise compartment test - Soil-Endosphere |  |  |  |  | 0.0027863 |
|  | Pairwise compartment test - Soil-Rhizosphere |  |  |  |  | 0.7161791 |

**Table S13.** Log fold change (LFC) of differentially abundant bacterial phyla across bulk soils, rhizospheres and endospheres. Significance value is indicated by ‘*’ for p < 0.05, ‘**’ for p < 0.01, and ‘***’ for p < 0.001. NS indicates non-significant taxa within a given comparison.

| **Bacterial Phylum** | **LFC Rhizosphere vs. Endosphere** | **LFC Soil vs. Endosphere** | **LFC Soil vs. Rhizosphere** |
| --- | --- | --- | --- |
| Coprothermobacterota | 0.479 NS | 0.824*** | 0.345 NS |
| Chrysiogenota | 0.453* | 0.482** | 0.029 NS |
| Caldisericota | 0.869** | 1.8*** | 0.931** |
| Nitrospinota | 0.609** | 0.203 NS | -0.407* |
| Atribacterota | 1.151*** | 1.915*** | 0.764* |
| Elusimicrobiota | 0.71** | 1.496*** | 0.786** |
| Dictyoglomota | 0.795* | 1.783*** | 0.987** |
| Calditrichota | 1.085*** | 1.292*** | 0.207 NS |
| Aquificota | 0.694*** | 1.355*** | 0.661*** |
| Deferribacterota | 0.755** | 1.481*** | 0.726** |
| Candidatus_Bipolaricaulota | 0.082 NS | -0.906*** | -0.988*** |
| Candidatus_Fervidibacteria | 0.854*** | 0.981*** | 0.127 NS |
| Candidatus_Absconditabacteria | 0.756* | 1.565*** | 0.809* |
| Candidatus_Saccharibacteria | 1.04** | 1.055*** | 0.015 NS |
| Bdellovibrionota | 0.699** | 1.134*** | 0.435* |
| Thermotogota | 0.818*** | 1.515*** | 0.697** |
| Nitrospirota | 0.834*** | 0.798*** | -0.036 NS |
| Fusobacteriota | 0.465* | 1.387*** | 0.923*** |
| Myxococcota | -1.02*** | -1.984*** | -0.964** |
| Spirochaetota | 0.546** | 0.991*** | 0.446* |
| Acidobacteriota | 0.41 NS | -0.438 NS | -0.848* |
| Thermodesulfobacteriota | 0.254 NS | 0.125 NS | -0.129 NS |
| Campylobacterota | 0.555* | 1.282*** | 0.727** |
| Candidatus_Omnitrophota | 0.965*** | 0.848*** | -0.117 NS |
| Lentisphaerota | 0.786*** | 1.376*** | 0.59* |
| Kiritimatiellota | 0.438* | 0.205 NS | -0.233 NS |
| Chlamydiota | 1.192*** | 2.084*** | 0.893*** |
| Verrucomicrobiota | 0.321 NS | -0.123 NS | -0.444* |
| Planctomycetota | -0.055 NS | -0.661*** | -0.606* |
| Pseudomonadota | -0.735*** | -1.374*** | -0.639** |
| Fibrobacterota | 0.808*** | 0.925*** | 0.116 NS |
| Candidatus_Cloacimonadota | 0.9** | 1.692*** | 0.792** |
| Gemmatimonadota | 0.047 NS | -1.201*** | -1.247** |
| Balneolota | 0.827*** | 0.926*** | 0.099 NS |
| Rhodothermota | 0.054 NS | -0.627*** | -0.681** |
| Ignavibacteriota | 1.438*** | 2.033*** | 0.596* |
| Chlorobiota | 0.675*** | 0.675*** | -0.001 NS |
| Bacteroidota | 0.929*** | 1.231*** | 0.302 NS |
| Thermomicrobiota | 0.024 NS | -0.714*** | -0.738* |
| Armatimonadota | 0.562* | -0.092 NS | -0.654* |
| Deinococcota | -0.328 NS | -0.972*** | -0.644* |
| Chloroflexota | 0.346* | 0.618*** | 0.272 NS |
| Mycoplasmatota | 0.455* | 1.319*** | 0.864** |
| Cyanobacteriota | 0.712*** | 1.234*** | 0.522** |
| Bacillota | 0.623*** | 1.094*** | 0.47* |
| Actinomycetota | -1.794*** | -2.691*** | -0.897* |

**Table S14.** Statistical output for differential abundance analysis of each functional subsystem, across each plant compartment (soil, rhizosphere, and endosphere). Table shows statistical output for global models from ANCOMBC differential abundance analysis, and pairwise log fold change differences.

| **Subsystem 1** | **Subsystem 3** | **Test statistic (W)** | **P value** | **Adjusted P value** | **LFC Endosphere vs Rhizosphere** | **LFC Endosphere vs Soil** | **LFC Soil vs Rhizosphere** |
| --- | --- | --- | --- | --- | --- | --- | --- |
| Motility and Chemotaxis | Archaeal Flagellum | 23.874 | 4E-06 | 4E-06 | 0.521 | 0.876 | 0.355 |
| Motility and Chemotaxis | Bacterial Chemotaxis | 90.032 | 6E-23 | 4E-22 | -0.449 | -0.830 | -0.381 |
| Motility and Chemotaxis | Bacterial motility:Gliding | 68.997 | 2E-19 | 7E-19 | 0.675 | 0.606 | -0.070 |
| Motility and Chemotaxis | Flagellar motility | 25.574 | 2E-09 | 3E-09 | -0.199 | -0.458 | -0.259 |
| Motility and Chemotaxis | Flagellum | 19.312 | 2E-07 | 2E-07 | -0.224 | -0.327 | -0.102 |
| Motility and Chemotaxis | Flagellum in Campylobacter | 39.728 | 3E-13 | 7E-13 | -0.469 | -0.634 | -0.165 |
| Nitrogen metabolism | Allantoin Utilization | 46.818 | 8E-15 | 2E-14 | -0.260 | -0.770 | -0.510 |
| Nitrogen metabolism | Amidase clustered with urea and nitrile hydratase functions | 110.377 | 6E-26 | 4E-25 | -0.767 | -1.960 | -1.193 |
| Nitrogen metabolism | Ammonia assimilation | 22.530 | 2E-08 | 3E-08 | 0.193 | -0.458 | -0.650 |
| Nitrogen metabolism | Cyanate hydrolysis | 6.088 | 6E-03 | 6E-03 | 0.093 | -0.240 | -0.333 |
| Nitrogen metabolism | Denitrification | 6.869 | 3E-03 | 3E-03 | 0.569 | 0.120 | -0.449 |
| Nitrogen metabolism | Dissimilatory nitrite reductase | 25.879 | 2E-09 | 3E-09 | -0.358 | -0.653 | -0.295 |
| Nitrogen metabolism | Nitrate and nitrite ammonification | 69.727 | 2E-19 | 7E-19 | 0.026 | -0.833 | -0.860 |
| Nitrogen metabolism | Nitric oxide synthase | 180.086 | 7E-34 | 8E-33 | -0.914 | -1.425 | -0.510 |
| Nitrogen metabolism | Nitrilase | 9.940 | 2E-04 | 3E-04 | 0.452 | -0.263 | -0.715 |
| Nitrogen metabolism | Nitrogen fixation | 26.420 | 1E-09 | 3E-09 | 0.794 | 0.400 | -0.394 |
| Nitrogen metabolism | Nitrogen Metabolism in Aspergillus nidulans | 8.452 | 8E-04 | 1E-03 | -0.643 | -0.522 | 0.121 |
| Nitrogen metabolism | Nitrosative stress | 16.237 | 2E-06 | 2E-06 | 0.681 | -0.194 | -0.876 |
| Phosphorus metabolism | High affinity phosphate transporter and control of PHO regulon | 22.747 | 1E-08 | 8E-08 | 0.144 | 0.423 | 0.279 |
| Phosphorus metabolism | P uptake (cyanobacteria) | 14.151 | 2E-05 | 3E-05 | 0.703 | 0.816 | 0.113 |
| Phosphorus metabolism | Phosphate metabolism | 15.917 | 2E-06 | 4E-06 | 0.155 | 0.320 | 0.165 |
| Phosphorus metabolism | Phosphonate metabolism | 21.438 | 4E-08 | 1E-07 | -0.313 | -0.660 | -0.347 |
| Secondary metabolism | 2-isocapryloyl-3R-hydroxymethyl-gamma-butyrolactone and other bacterial morphogens | 145.431 | 2E-21 | 9E-21 | -1.782 | -2.480 | -0.699 |
| Secondary metabolism | Alkaloid biosynthesis from L-lysine | 72.838 | 4E-20 | 2E-19 | 0.873 | 1.434 | 0.561 |
| Secondary metabolism | Apigenin derivatives | 4.609 | 3E-02 | 4E-02 | -0.205 | 0.348 | 0.554 |
| Secondary metabolism | Auxin biosynthesis | 91.308 | 2E-16 | 5E-16 | -1.829 | -2.190 | -0.361 |
| Secondary metabolism | Auxin degradation | 8.747 | 6E-04 | 9E-04 | -0.156 | -0.527 | -0.371 |
| Secondary metabolism | Biflavanoid biosynthesis | 21.011 | 5E-08 | 8E-08 | -0.582 | -0.829 | -0.247 |
| Secondary metabolism | Caffeic acid derivatives | 110.847 | 7E-24 | 5E-23 | -1.790 | -1.888 | -0.099 |
| Secondary metabolism | Clavulanic acid biosynthesis | 51.818 | 5E-15 | 1E-14 | -1.311 | -1.388 | -0.076 |
| Secondary metabolism | Flavanones and dihydroflavonols biosynthesis in plants | 17.417 | 2E-05 | 2E-05 | -0.258 | -0.867 | -0.609 |
| Secondary metabolism | Homomethionine biosynthesis and methionine chain elongation pathway for glucosinolates in plants | 54.358 | 1E-10 | 2E-10 | -0.901 | -1.600 | -0.699 |
| Secondary metabolism | Nonribosomal peptide synthetases (NRPS) in Frankia sp. Ccl3 | 204.046 | 4E-29 | 8E-28 | -2.536 | -2.981 | -0.445 |
| Secondary metabolism | Paerucumarin Biosynthesis | 5.402 | 1E-02 | 2E-02 | -0.569 | -0.162 | 0.407 |
| Secondary metabolism | Phenazine biosynthesis | 35.076 | 5E-12 | 1E-11 | -0.806 | -1.151 | -0.345 |
| Secondary metabolism | Phenylpropanoids general biosynthesis in plants | 157.371 | 1E-26 | 1E-25 | -2.333 | -2.364 | -0.031 |
| Secondary metabolism | Phenylpropionate Degradation | 67.171 | 6E-17 | 2E-16 | -1.341 | -1.633 | -0.292 |
| Secondary metabolism | Salicylic acid biosynthesis1 | 16.722 | 2E-06 | 3E-06 | -0.956 | -0.842 | 0.114 |
| Secondary metabolism | Sinapate ester biosynthesis in plants | 110.145 | 5E-21 | 2E-20 | -2.519 | -2.001 | 0.517 |
| Secondary metabolism | Tannin biosynthesis | 21.011 | 5E-08 | 8E-08 | -0.582 | -0.829 | -0.247 |
| Regulation and cell signalling | A conserved operon linked to TyrR and possibly involved in virulence | 44.364 | 4E-07 | 7E-07 | 0.412 | -0.757 | -1.170 |
| Regulation and cell signalling | Acyl Homoserine Lactone (AHL) Autoinducer Quorum Sensing_ | 14.921 | 8E-06 | 1E-05 | 0.048 | -0.583 | -0.631 |
| Regulation and cell signalling | Autoinducer 2 (AI-2) transport and processing (lsrACDBFGE operon) | 39.295 | 7E-13 | 2E-12 | 0.908 | 1.595 | 0.687 |
| Regulation and cell signalling | Bacterial Caspases | 6.951 | 4E-03 | 5E-03 | 0.191 | -0.324 | -0.515 |
| Regulation and cell signalling | Biofilm Adhesin Biosynthesis | 9.597 | 5E-04 | 8E-04 | -0.010 | -0.484 | -0.474 |
| Regulation and cell signalling | Biofilm formation in Staphylococcus | 318.381 | 3E-42 | 4E-41 | -1.277 | -2.957 | -1.681 |
| Regulation and cell signalling | cAMP signaling in bacteria | 4.604 | 2E-02 | 3E-02 | 0.242 | -0.145 | -0.387 |
| Regulation and cell signalling | Cell envelope-associated LytR-CpsA-Psr transcriptional attenuators | 58.856 | 2E-17 | 6E-17 | -0.749 | -1.282 | -0.533 |
| Regulation and cell signalling | Coagulation cascade | 6.302 | 9E-03 | 1E-02 | 0.541 | 0.028 | -0.513 |
| Regulation and cell signalling | CytR regulation | 32.491 | 7E-10 | 2E-09 | -0.067 | -0.915 | -0.848 |
| Regulation and cell signalling | DNA-binding regulatory proteins, strays | 103.573 | 6E-25 | 4E-24 | -0.558 | -1.538 | -0.980 |
| Regulation and cell signalling | G-protein-coupled receptor (GPCR) system in Actinobacteria | 662.394 | 5E-51 | 2E-49 | -2.840 | -4.664 | -1.824 |
| Regulation and cell signalling | Global Two-component Regulator PrrBA in Proteobacteria | 438.940 | 7E-46 | 1E-44 | -1.138 | -3.187 | -2.049 |
| Regulation and cell signalling | HPr catabolite repression system | 9.896 | 2E-04 | 4E-04 | 0.524 | 0.251 | -0.273 |
| Regulation and cell signalling | Iojap | 5.976 | 7E-03 | 9E-03 | 0.328 | -0.159 | -0.487 |
| Regulation and cell signalling | MazEF toxin-antitoxing (programmed cell death) system | 84.684 | 4E-22 | 2E-21 | 1.100 | 1.187 | 0.087 |
| Regulation and cell signalling | Murein hydrolase regulation and cell death | 81.755 | 2E-21 | 7E-21 | -0.454 | -1.095 | -0.641 |
| Regulation and cell signalling | Orphan regulatory proteins | 40.181 | 3E-13 | 8E-13 | -0.132 | -0.771 | -0.638 |
| Regulation and cell signalling | Oxygen and light sensor PpaA-PpsR | 25.022 | 3E-09 | 7E-09 | 0.712 | 0.427 | -0.285 |
| Regulation and cell signalling | P38 MAP kinase pathways | 9.067 | 5E-04 | 7E-04 | 0.287 | 0.552 | 0.265 |
| Regulation and cell signalling | Phd-Doc, YdcE-YdcD toxin-antitoxin (programmed cell death) systems | 29.771 | 1E-10 | 4E-10 | 0.647 | 0.550 | -0.098 |
| Regulation and cell signalling | Phosphoinositides biosynthesis in plants | 4.052 | 4E-02 | 5E-02 | -0.521 | 0.008 | 0.529 |
| Regulation and cell signalling | Plastidial (p)ppGpp-mediated response in plants | 19.437 | 1E-07 | 3E-07 | -0.023 | -0.598 | -0.575 |
| Regulation and cell signalling | Quorum sensing in Yersinia | 12.947 | 2E-05 | 4E-05 | 0.518 | 0.474 | -0.045 |
| Regulation and cell signalling | Rcs phosphorelay signal transduction pathway | 10.875 | 1E-04 | 2E-04 | 0.228 | -0.536 | -0.764 |
| Regulation and cell signalling | Sex pheromones in Enterococcus faecalis and other Firmicutes | 157.903 | 1E-31 | 1E-30 | -0.595 | -1.423 | -0.828 |
| Regulation and cell signalling | Stringent Response, (p)ppGpp metabolism | 9.938 | 2E-04 | 4E-04 | 0.474 | 0.118 | -0.356 |
| Regulation and cell signalling | Symbiotic colonization and sigma-dependent biofilm formation gene cluster | 5.699 | 1E-02 | 1E-02 | 0.242 | -0.235 | -0.477 |
| Regulation and cell signalling | The Chv regulatory system of Alphaproteobacteria | 110.951 | 1E-25 | 9E-25 | -0.631 | -2.022 | -1.391 |
| Regulation and cell signalling | Toxin-antitoxin replicon stabilization systems | 20.147 | 9E-07 | 2E-06 | 1.005 | 0.586 | -0.419 |
| Regulation and cell signalling | Toxin-antitoxin system in Mycobacterium | 72.721 | 2E-18 | 8E-18 | -0.381 | -1.390 | -1.009 |
| Regulation and cell signalling | Toxin-antitoxin systems (other than RelBE and MazEF) | 61.093 | 7E-18 | 2E-17 | 1.011 | 0.832 | -0.179 |
| Regulation and cell signalling | Trans-envelope signaling system VreARI in Pseudomonas | 70.536 | 6E-18 | 2E-17 | -0.511 | -1.777 | -1.266 |
| Regulation and cell signalling | Two-component regulatory systems in Campylobacter | 21.357 | 4E-08 | 8E-08 | 0.606 | 0.394 | -0.212 |
| Regulation and cell signalling | Two-component Response Regulator of Virulence ResDE | 114.817 | 2E-23 | 1E-22 | 1.380 | 2.078 | 0.698 |
| Regulation and cell signalling | WhiB and WhiB-type regulatory proteins_ | 5.741 | 9E-03 | 1E-02 | 0.286 | 0.221 | -0.065 |
| Regulation and cell signalling | Zinc regulated enzymes | 4.717 | 2E-02 | 3E-02 | 0.009 | -0.225 | -0.234 |
| Stress response | At5g63290 | 30.123 | 1E-10 | 2E-10 | 0.436 | 0.359 | -0.077 |
| Stress response | Bacterial hemoglobins | 79.606 | 3E-21 | 1E-20 | -0.567 | -0.774 | -0.206 |
| Stress response | Carbon Starvation | 24.907 | 3E-09 | 6E-09 | 0.027 | -0.528 | -0.555 |
| Stress response | Choline and Betaine Uptake and Betaine Biosynthesis | 150.352 | 8E-31 | 7E-30 | -0.614 | -1.014 | -0.399 |
| Stress response | Cold shock, CspA family of proteins | 96.177 | 7E-24 | 4E-23 | 0.783 | 0.744 | -0.039 |
| Stress response | Commensurate regulon activation | 4.878 | 3E-02 | 3E-02 | 0.386 | 0.375 | -0.011 |
| Stress response | D-tyrosyl-tRNA(Tyr) deacylase | 74.126 | 4E-20 | 1E-19 | 0.786 | 0.806 | 0.020 |
| Stress response | Dimethylarginine metabolism | 5.332 | 1E-02 | 2E-02 | -0.250 | -0.194 | 0.056 |
| Stress response | Ectoine biosynthesis and regulation | 223.447 | 6E-36 | 7E-35 | -1.522 | -2.116 | -0.594 |
| Stress response | Flavohaemoglobin | 55.767 | 9E-17 | 2E-16 | -0.315 | -0.671 | -0.356 |
| Stress response | FOL Commensurate regulon activation | 9.338 | 4E-04 | 6E-04 | -0.009 | -0.496 | -0.488 |
| Stress response | Glutamate transporter involved in acid tolerance in Streptococcus | 44.877 | 1E-07 | 2E-07 | 0.966 | -0.080 | -1.046 |
| Stress response | Glutathione analogs: mycothiol | 511.520 | 3E-49 | 1E-47 | -2.443 | -3.722 | -1.279 |
| Stress response | Glutathione-dependent pathway of formaldehyde detoxification | 205.311 | 5E-36 | 7E-35 | -0.757 | -1.466 | -0.709 |
| Stress response | Glutathione: Biosynthesis and gamma-glutamyl cycle | 86.491 | 2E-22 | 1E-21 | -0.379 | -0.732 | -0.353 |
| Stress response | Glutathione: Non-redox reactions | 73.049 | 4E-20 | 2E-19 | -0.454 | -0.706 | -0.251 |
| Stress response | Glutathionylspermidine and Trypanothione | 101.258 | 6E-24 | 4E-23 | -0.457 | -1.448 | -0.991 |
| Stress response | Gycosylglycerates | 12.866 | 2E-05 | 4E-05 | 0.575 | 0.675 | 0.101 |
| Stress response | Heat shock dnaK gene cluster extended | 20.420 | 7E-08 | 1E-07 | 0.257 | 0.296 | 0.039 |
| Stress response | Hfl operon | 5.171 | 1E-02 | 2E-02 | 0.104 | 0.167 | 0.063 |
| Stress response | Housecleaning nucleoside triphosphate pyrophosphatases | 8.075 | 1E-03 | 1E-03 | 0.124 | 0.248 | 0.124 |
| Stress response | Nucleoside triphosphate pyrophosphohydrolase MazG | 12.449 | 3E-05 | 5E-05 | 0.163 | 0.364 | 0.200 |
| Stress response | Nudix KE | 11.854 | 5E-05 | 7E-05 | -0.120 | -0.285 | -0.165 |
| Stress response | Nudix proteins (nucleoside triphosphate hydrolases) | 107.852 | 1E-25 | 1E-24 | -0.473 | -0.731 | -0.259 |
| Stress response | O-antigen capsule important for environmental persistence | 5.752 | 2E-02 | 2E-02 | -0.471 | -0.448 | 0.024 |
| Stress response | Osmoprotectant ABC transporter YehZYXW of Enterobacteriales | 101.394 | 3E-19 | 1E-18 | -0.228 | -1.419 | -1.191 |
| Stress response | Osmoregulation | 10.347 | 2E-04 | 2E-04 | 0.092 | 0.322 | 0.230 |
| Stress response | Osmotic stress cluster | 30.560 | 2E-10 | 4E-10 | -0.446 | -0.894 | -0.447 |
| Stress response | Oxidative stress | 9.698 | 3E-04 | 4E-04 | 0.172 | 0.193 | 0.021 |
| Stress response | Oxygen stress response / Human gut microbiome | 30.906 | 8E-11 | 2E-10 | 0.483 | 0.779 | 0.295 |
| Stress response | Periplasmic Acid Stress Response in Enterobacteria | 23.817 | 2E-06 | 3E-06 | -0.042 | 0.593 | 0.635 |
| Stress response | Periplasmic Stress Response | 52.341 | 5E-16 | 1E-15 | 0.392 | 0.574 | 0.182 |
| Stress response | Phage shock protein (psp) operon | 61.473 | 1E-17 | 4E-17 | 0.007 | -1.018 | -1.025 |
| Stress response | Redox-dependent regulation of nucleus processes | 7.726 | 2E-03 | 2E-03 | -0.161 | -0.189 | -0.028 |
| Stress response | Regulation of Oxidative Stress Response | 9.146 | 4E-04 | 6E-04 | -0.050 | -0.273 | -0.223 |
| Stress response | Rubrerythrin | 65.849 | 9E-19 | 3E-18 | 0.526 | 0.681 | 0.155 |
| Stress response | SigmaB stress responce regulation | 62.569 | 4E-18 | 1E-17 | -0.364 | -0.613 | -0.249 |
| Stress response | Synthesis of osmoregulated periplasmic glucans | 15.679 | 2E-06 | 4E-06 | 0.028 | -0.402 | -0.430 |
| Stress response | Tellurite resistance: Chromosomal determinants | 12.926 | 3E-05 | 5E-05 | 0.001 | 0.847 | 0.846 |
| Stress response | Uptake of selenate and selenite | 223.777 | 1E-37 | 2E-36 | -0.710 | -1.261 | -0.551 |

**Table S15.** Statistical output for Kruskal-Wallis test and Dunn’s Test comparing edge average weights from the network analysis of functional genes from subsystem 1 with. These tests compared bulk soils, rhizospheres and endospheres networks low, medium and high aridity levels. Global significance denotes significance level at P<0.05 = ‘*’, P<0.01 = ‘**’, P<0.001 = ‘***’, and P≥0.05 = ‘NS’ (non-significant result).

| **Subsystem 1** | **Zone** | **Test** | **Aridity level comparison** | **df** | **Test statistic** | **Statistic value** | **P value** | **Adj. P value** | **Global Significance** | |
| --- | --- | --- | --- | --- | --- | --- | --- | --- | --- | --- |
| Motility and chemotaxis | Soil | Kruskal-Wallis | | 2 | χ2 | 4.696 | 0.096 |  | NS | |
|  | Rhizosphere | Kruskal-Wallis | | 2 | χ2 | 15.628 | 0.000 |  | *** | |
|  |  | Dunn's Test | High aridity - Low aridity | | z | -3.773 | 0.000 | 0.000 |  | |
|  |  |  | High aridity - Medium aridity | | z | -3.428 | 0.001 | 0.001 |  | |
|  |  |  | Low aridity - Medium aridity | | z | 1.230 | 0.219 | 0.219 |  | |
|  | Endosphere | Kruskal-Wallis | | 2 | χ2 | 9.732 | 0.008 |  | ** | |
|  |  | Dunn's Test | High aridity - Low aridity | | z | 3.045 | 0.002 | 0.007 |  | |
|  |  |  | High aridity - Medium aridity | | z | 2.511 | 0.012 | 0.024 |  | |
|  |  |  | Low aridity - Medium aridity | | z | -0.729 | 0.466 | 0.466 |  | |
| Nitrogen | Soil | Kruskal-Wallis | | 2 | χ2 | 4.164 | 0.125 |  | NS | |
|  | Rhizosphere |  |  | 2 | χ2 | 0.523 | 0.770 |  | NS | |
|  | Endosphere |  |  | 2 | χ2 | 7.750 | 0.021 |  | * | |
|  |  | Dunn's Test | High aridity - Low aridity | | z | -2.363 | 0.018 | 0.036 |  | |
|  |  |  | High aridity - Medium aridity | | z | -2.698 | 0.007 | 0.021 |  | |
|  |  |  | Low aridity - Medium aridity | | z | -0.422 | 0.673 | 0.673 |  | |
| Phosphorus | Soil | Kruskal-Wallis | | 2 | χ2 | 1.678 | 0.432 |  | NS | |
|  | Rhizosphere | Kruskal-Wallis | | 2 | χ2 | 36.122 | 1.432e-08 | | *** | |
|  |  | Dunn's Test | High aridity - Low aridity | | z | -5.762 | 8.305e-09 | 2.491e-08 |  |  |
|  |  |  | High aridity - Medium aridity | | z | -5.061 | 4.176e-07 | 8.353e-07 |  |  |
|  |  |  | Low aridity - Medium aridity | | z | 1.495 | 0.135 | 0.135 |  |  |
|  | Endosphere | Kruskal-Wallis | | 2 | χ2 | 3.437 | 0.179 |  | NS |  |
| Secondary metabolism | Soil | Kruskal-Wallis | | 2 | χ2 | 3.822 | 0.148 |  | NS | |
|  | Rhizosphere | Kruskal-Wallis | | 2 | χ2 | 10.547 | 0.005 |  | ** | |
|  |  | Dunn's Test | High aridity - Low aridity | | z | 3.190 | 0.001 | 0.004 |  | |
|  |  |  | High aridity - Medium aridity | | z | 0.984 | 0.325 | 0.325 |  | |
|  |  |  | Low aridity - Medium aridity | | z | -2.252 | 0.024 | 0.049 |  | |
|  | Endosphere | Kruskal-Wallis | | 2 | χ2 | 1.031 | 0.597 |  | NS | |
| Regulation and cell signalling | Soil | Kruskal-Wallis | | 2 | χ2 | 7.107 | 0.029 |  | * | |
|  |  | Dunn's Test | High aridity - Low aridity | | z | -2.098 | 0.036 | 0.108 |  | |
|  |  |  | High aridity - Medium aridity | | z | -1.627 | 0.104 | 0.104 |  | |
|  |  |  | Low aridity - Medium aridity | | z | 1.843 | 0.065 | 0.131 |  | |
|  | Rhizosphere | Kruskal-Wallis | | 2 | χ2 | 60.927 | 5.886e-14 | | *** | |
|  |  | Dunn's Test | High aridity - Low aridity | | z | -7.760 | 8.526e-15 | 2.558e-14 |  |  |
|  |  |  | High aridity - Medium aridity | | z | -6.831 | 8.445e-12 | 1.689e-11 |  |  |
|  |  |  | Low aridity - Medium aridity | | z | 2.506 | 0.012 | 0.012 |  | |
|  | Endosphere | Kruskal-Wallis | | 2 | χ2 | 6.949 | 0.031 |  | * | |
|  |  | Dunn's Test | High aridity - Low aridity | | z | -2.491 | 0.013 | 0.038 |  | |
|  |  |  | High aridity - Medium aridity | | z | -2.169 | 0.030 | 0.060 |  | |
|  |  |  | Low aridity - Medium aridity | | z | 0.077 | 0.939 | 0.939 |  | |
| Stress response | Soil | Kruskal-Wallis | | 2 | χ2 | 4.232 | 0.120 |  | NS | |
|  | Rhizosphere | Kruskal-Wallis | | 2 | χ2 | 51.016 | 8.355e-12 | | *** | |
|  |  | Dunn Test | High aridity - Low aridity | | Z | -6.891 | 5.526e-12 | 1.658e-11 |  |  |
|  |  |  | High aridity - Medium aridity | | Z | -6.515 | 7.290e-11 | 1.458e-10 |  |  |
|  |  |  | Low aridity - Medium aridity | | Z | 1.748 | 0.080 | 0.080 |  | |
|  | Endosphere | Kruskal-Wallis | | 2 | χ2 | 7.785 | 0.020 |  | * | |
|  |  | Dunn Test | High aridity - Low aridity | | Z | -2.788 | 0.005 | 0.016 |  | |
|  |  |  | High aridity - Medium aridity | | Z | -1.762 | 0.078 | 0.156 |  | |
|  |  |  | Low aridity - Medium aridity | | Z | 0.993 | 0.321 | 0.321 |  | |

**Table S16.** Hub functions for the stress response (subsystem 1) networks for each plant compartment (bulk soil, rhizosphere and endosphere) at three different aridity levels (low aridity, medium aridity, and high aridity). The top 20 bub functions in each network were selected based on highest node degree, then closeness centrality.

| **Compartment** | **Aridity** | **Function** | **Subsystem 2** | **Subsystem 3** | **Degree** | **Closeness centrality** |
| --- | --- | --- | --- | --- | --- | --- |
| Bulk soil~ | Low aridity~ | FUN28191 | - | Dimethylarginine metabolism | 108 | 0.0049 |
|  |  | FUN28255 | - | Flavohaemoglobin | 111 | 0.0053 |
|  |  | FUN28405 | - | Universal stress protein family | 114 | 0.005 |
|  |  | FUN28464 | Detoxification | Housecleaning nucleoside triphosphate pyrophosphatases | 124 | 0.0051 |
|  |  | FUN28467 | Detoxification | Housecleaning nucleoside triphosphate pyrophosphatases | 110 | 0.0052 |
|  |  | FUN28636 | Heat shock | At5g63290 | 113 | 0.0049 |
|  |  | FUN28649 | Heat shock | At5g63290 | 119 | 0.0052 |
|  |  | FUN28655 | Heat shock | At5g63290 | 108 | 0.0051 |
|  |  | FUN28661 | Heat shock | At5g63290 | 112 | 0.0054 |
|  |  | FUN28686 | Heat shock | Heat shock dnaK gene cluster extended | 106 | 0.0052 |
|  |  | FUN28697 | Heat shock | Heat shock dnaK gene cluster extended | 112 | 0.0052 |
|  |  | FUN28706 | Heat shock | Heat shock dnaK gene cluster extended | 117 | 0.0053 |
|  |  | FUN28707 | Heat shock | Heat shock dnaK gene cluster extended | 107 | 0.0049 |
|  |  | FUN28717 | Heat shock | Heat shock dnaK gene cluster extended | 110 | 0.0051 |
|  |  | FUN28718 | Heat shock | Heat shock dnaK gene cluster extended | 108 | 0.0048 |
|  |  | FUN28719 | Heat shock | Heat shock dnaK gene cluster extended | 110 | 0.0048 |
|  |  | FUN28725 | Heat shock | Heat shock dnaK gene cluster extended | 118 | 0.005 |
|  |  | FUN28735 | Heat shock | Heat shock dnaK gene cluster extended | 114 | 0.0052 |
|  |  | FUN28871 | Osmotic stress | Osmoregulation | 108 | 0.0052 |
|  |  | FUN28893 | Osmotic stress | Synthesis of osmoregulated periplasmic glucans | 107 | 0.0053 |
|  |  | FUN28901 | Oxidative stress | Glutaredoxins | 114 | 0.0054 |
|  |  | FUN29012 | Oxidative stress | Glutathione: Biosynthesis and gamma-glutamyl cycle | 112 | 0.0049 |
|  |  | FUN29123 | Oxidative stress | Oxidative stress | 123 | 0.0053 |
|  |  | FUN29158 | Oxidative stress | Oxidative stress | 107 | 0.0052 |
|  |  | FUN29191 | Oxidative stress | Oxidative stress | 108 | 0.0051 |
|  |  | FUN29240 | Oxidative stress | Oxidative stress | 112 | 0.0051 |
|  |  | FUN29356 | Oxidative stress | Rubrerythrin | 117 | 0.0049 |
|  |  | FUN29365 | Oxidative stress | Rubrerythrin | 126 | 0.0053 |
|  |  | FUN29376 | Periplasmic Stress | Periplasmic Stress Response | 129 | 0.0052 |
|  |  | FUN29382 | Periplasmic Stress | Periplasmic Stress Response | 110 | 0.0049 |
|  | Medium aridity~ | FUN281911 | - | Dimethylarginine metabolism | 111 | 0.0066 |
|  |  | FUN282721 | - | Flavohaemoglobin | 110 | 0.0067 |
|  |  | FUN283001 | - | Flavohaemoglobin | 111 | 0.007 |
|  |  | FUN283311 | - | Hfl operon | 115 | 0.0066 |
|  |  | FUN284641 | Detoxification | Housecleaning nucleoside triphosphate pyrophosphatases | 115 | 0.0065 |
|  |  | FUN284671 | Detoxification | Housecleaning nucleoside triphosphate pyrophosphatases | 109 | 0.0065 |
|  |  | FUN286031 | Detoxification | Uptake of selenate and selenite | 110 | 0.0063 |
|  |  | FUN286301 | Heat shock | At5g63290 | 115 | 0.0066 |
|  |  | FUN286361 | Heat shock | At5g63290 | 117 | 0.0064 |
|  |  | FUN286411 | Heat shock | At5g63290 | 116 | 0.007 |
|  |  | FUN286491 | Heat shock | At5g63290 | 104 | 0.0068 |
|  |  | FUN286531 | Heat shock | At5g63290 | 112 | 0.0065 |
|  |  | FUN286581 | Heat shock | At5g63290 | 106 | 0.0065 |
|  |  | FUN286861 | Heat shock | Heat shock dnaK gene cluster extended | 118 | 0.0069 |
|  |  | FUN286971 | Heat shock | Heat shock dnaK gene cluster extended | 114 | 0.0069 |
|  |  | FUN287061 | Heat shock | Heat shock dnaK gene cluster extended | 109 | 0.007 |
|  |  | FUN287131 | Heat shock | Heat shock dnaK gene cluster extended | 106 | 0.0066 |
|  |  | FUN287171 | Heat shock | Heat shock dnaK gene cluster extended | 113 | 0.0068 |
|  |  | FUN287251 | Heat shock | Heat shock dnaK gene cluster extended | 116 | 0.0067 |
|  |  | FUN287711 | Osmotic stress | Choline and Betaine Uptake and Betaine Biosynthesis | 112 | 0.0065 |
|  |  | FUN288571 | Osmotic stress | Gycosylglycerates | 104 | 0.0066 |
|  |  | FUN288881 | Osmotic stress | Synthesis of osmoregulated periplasmic glucans | 108 | 0.0062 |
|  |  | FUN289541 | Oxidative stress | Glutaredoxins | 117 | 0.0066 |
|  |  | FUN290981 | Oxidative stress | Glutathione: Redox cycle | 104 | 0.0066 |
|  |  | FUN291581 | Oxidative stress | Oxidative stress | 110 | 0.0065 |
|  |  | FUN291951 | Oxidative stress | Oxidative stress | 111 | 0.0066 |
|  |  | FUN292911 | Oxidative stress | Protection from Reactive Oxygen Species | 114 | 0.0065 |
|  |  | FUN293251 | Oxidative stress | Redox-dependent regulation of nucleus processes | 112 | 0.0068 |
|  |  | FUN293561 | Oxidative stress | Rubrerythrin | 107 | 0.0066 |
|  |  | FUN293681 | Oxidative stress | Rubrerythrin | 104 | 0.0063 |
|  | High aridity~ | FUN281542 | - | Bacterial hemoglobins | 22 | 0.0085 |
|  |  | FUN281912 | - | Dimethylarginine metabolism | 14 | 0.0079 |
|  |  | FUN283312 | - | Hfl operon | 18 | 0.0085 |
|  |  | FUN283732 | - | SigmaB stress responce regulation | 12 | 0.0083 |
|  |  | FUN283802 | - | SigmaB stress responce regulation | 14 | 0.0085 |
|  |  | FUN284052 | - | Universal stress protein family | 23 | 0.0085 |
|  |  | FUN284192 | Cold shock | Cold shock, CspA family of proteins | 20 | 0.0085 |
|  |  | FUN284232 | Cold shock | Cold shock, CspA family of proteins | 17 | 0.0092 |
|  |  | FUN284902 | Detoxification | Nucleoside triphosphate pyrophosphohydrolase MazG | 9 | 0.0081 |
|  |  | FUN285002 | Detoxification | Nudix KE | 25 | 0.0093 |
|  |  | FUN286362 | Heat shock | At5g63290 | 15 | 0.0093 |
|  |  | FUN286532 | Heat shock | At5g63290 | 12 | 0.0083 |
|  |  | FUN286712 | Heat shock | At5g63290 | 9 | 0.0083 |
|  |  | FUN286772 | Heat shock | At5g63290 | 16 | 0.0085 |
|  |  | FUN287072 | Heat shock | Heat shock dnaK gene cluster extended | 18 | 0.0086 |
|  |  | FUN287112 | Heat shock | Heat shock dnaK gene cluster extended | 17 | 0.0087 |
|  |  | FUN287352 | Heat shock | Heat shock dnaK gene cluster extended | 14 | 0.0081 |
|  |  | FUN287732 | Osmotic stress | Choline and Betaine Uptake and Betaine Biosynthesis | 25 | 0.0096 |
|  |  | FUN287962 | Osmotic stress | Choline and Betaine Uptake and Betaine Biosynthesis | 16 | 0.0084 |
|  |  | FUN289342 | Oxidative stress | Glutaredoxins | 10 | 0.0086 |
|  |  | FUN289972 | Oxidative stress | Glutathione: Biosynthesis and gamma-glutamyl cycle | 21 | 0.0095 |
|  |  | FUN290302 | Oxidative stress | Glutathione: Non-redox reactions | 18 | 0.0085 |
|  |  | FUN290982 | Oxidative stress | Glutathione: Redox cycle | 19 | 0.0092 |
|  |  | FUN291232 | Oxidative stress | Oxidative stress | 21 | 0.0084 |
|  |  | FUN291752 | Oxidative stress | Oxidative stress | 9 | 0.0064 |
|  |  | FUN292872 | Oxidative stress | Protection from Reactive Oxygen Species | 9 | 0.0065 |
|  |  | FUN292932 | Oxidative stress | Protection from Reactive Oxygen Species | 9 | 0.0065 |
|  |  | FUN293562 | Oxidative stress | Rubrerythrin | 18 | 0.0083 |
|  |  | FUN293822 | Periplasmic Stress | Periplasmic Stress Response | 17 | 0.0088 |
|  |  | FUN293902 | Periplasmic Stress | Periplasmic Stress Response | 19 | 0.0084 |
| Rhizosphere | Low aridity~ | FUN281702 | - | Carbon Starvation | 63 | 0.0054 |
|  |  | FUN281772 | - | Carbon Starvation | 55 | 0.0054 |
|  |  | FUN281913 | - | Dimethylarginine metabolism | 58 | 0.0059 |
|  |  | FUN282452 | - | Flavohaemoglobin | 47 | 0.0055 |
|  |  | FUN283372 | - | Hfl operon | 67 | 0.0057 |
|  |  | FUN283722 | - | SigmaB stress responce regulation | 52 | 0.0057 |
|  |  | FUN284053 | - | Universal stress protein family | 55 | 0.0051 |
|  |  | FUN284193 | Cold shock | Cold shock, CspA family of proteins | 45 | 0.0053 |
|  |  | FUN284803 | Detoxification | Housecleaning nucleoside triphosphate pyrophosphatases | 44 | 0.0056 |
|  |  | FUN286202 | Detoxification | Uptake of selenate and selenite | 47 | 0.0054 |
|  |  | FUN286323 | Heat shock | At5g63290 | 50 | 0.0053 |
|  |  | FUN286552 | Heat shock | At5g63290 | 53 | 0.0058 |
|  |  | FUN287233 | Heat shock | Heat shock dnaK gene cluster extended | 53 | 0.0057 |
|  |  | FUN287362 | Heat shock | Heat shock dnaK gene cluster extended | 62 | 0.0056 |
|  |  | FUN287733 | Osmotic stress | Choline and Betaine Uptake and Betaine Biosynthesis | 49 | 0.0053 |
|  |  | FUN287862 | Osmotic stress | Choline and Betaine Uptake and Betaine Biosynthesis | 47 | 0.0052 |
|  |  | FUN287963 | Osmotic stress | Choline and Betaine Uptake and Betaine Biosynthesis | 43 | 0.0055 |
|  |  | FUN287992 | Osmotic stress | Choline and Betaine Uptake and Betaine Biosynthesis | 47 | 0.0052 |
|  |  | FUN288181 | Osmotic stress | Choline and Betaine Uptake and Betaine Biosynthesis | 51 | 0.0059 |
|  |  | FUN288552 | Osmotic stress | Gycosylglycerates | 46 | 0.005 |
|  |  | FUN288612 | Osmotic stress | Gycosylglycerates | 57 | 0.0055 |
|  |  | FUN289222 | Oxidative stress | Glutaredoxins | 59 | 0.0054 |
|  |  | FUN289343 | Oxidative stress | Glutaredoxins | 44 | 0.0053 |
|  |  | FUN290303 | Oxidative stress | Glutathione: Non-redox reactions | 67 | 0.0059 |
|  |  | FUN290422 | Oxidative stress | Glutathione: Non-redox reactions | 51 | 0.0057 |
|  |  | FUN290613 | Oxidative stress | Glutathione: Non-redox reactions | 48 | 0.0056 |
|  |  | FUN291682 | Oxidative stress | Oxidative stress | 67 | 0.0055 |
|  |  | FUN291912 | Oxidative stress | Oxidative stress | 53 | 0.0057 |
|  |  | FUN292263 | Oxidative stress | Oxidative stress | 67 | 0.0057 |
|  |  | FUN293222 | Oxidative stress | Redox-dependent regulation of nucleus processes | 48 | 0.0057 |
|  | Medium aridity~ | FUN281603 | - | Bacterial hemoglobins | 119 | 0.0052 |
|  |  | FUN281703 | - | Carbon Starvation | 116 | 0.0054 |
|  |  | FUN281773 | - | Carbon Starvation | 117 | 0.0054 |
|  |  | FUN281914 | - | Dimethylarginine metabolism | 117 | 0.0052 |
|  |  | FUN282723 | - | Flavohaemoglobin | 115 | 0.0053 |
|  |  | FUN284644 | Detoxification | Housecleaning nucleoside triphosphate pyrophosphatases | 124 | 0.0053 |
|  |  | FUN284673 | Detoxification | Housecleaning nucleoside triphosphate pyrophosphatases | 121 | 0.0054 |
|  |  | FUN285572 | Detoxification | Nudix proteins (nucleoside triphosphate hydrolases) | 115 | 0.0051 |
|  |  | FUN286304 | Heat shock | At5g63290 | 120 | 0.0053 |
|  |  | FUN286363 | Heat shock | At5g63290 | 120 | 0.0051 |
|  |  | FUN286864 | Heat shock | Heat shock dnaK gene cluster extended | 118 | 0.0051 |
|  |  | FUN287024 | Heat shock | Heat shock dnaK gene cluster extended | 115 | 0.0054 |
|  |  | FUN287074 | Heat shock | Heat shock dnaK gene cluster extended | 115 | 0.0053 |
|  |  | FUN287613 | Osmotic stress | Choline and Betaine Uptake and Betaine Biosynthesis | 115 | 0.0055 |
|  |  | FUN288884 | Osmotic stress | Synthesis of osmoregulated periplasmic glucans | 124 | 0.0054 |
|  |  | FUN289974 | Oxidative stress | Glutathione: Biosynthesis and gamma-glutamyl cycle | 118 | 0.0054 |
|  |  | FUN289992 | Oxidative stress | Glutathione: Biosynthesis and gamma-glutamyl cycle | 115 | 0.0052 |
|  |  | FUN290124 | Oxidative stress | Glutathione: Biosynthesis and gamma-glutamyl cycle | 116 | 0.005 |
|  |  | FUN290271 | Oxidative stress | Glutathione: Non-redox reactions | 117 | 0.0056 |
|  |  | FUN290984 | Oxidative stress | Glutathione: Redox cycle | 120 | 0.0052 |
|  |  | FUN291343 | Oxidative stress | Oxidative stress | 117 | 0.0054 |
|  |  | FUN291834 | Oxidative stress | Oxidative stress | 122 | 0.0051 |
|  |  | FUN291953 | Oxidative stress | Oxidative stress | 115 | 0.0053 |
|  |  | FUN292044 | Oxidative stress | Oxidative stress | 118 | 0.0053 |
|  |  | FUN292264 | Oxidative stress | Oxidative stress | 117 | 0.0052 |
|  |  | FUN292773 | Oxidative stress | Protection from Reactive Oxygen Species | 116 | 0.0053 |
|  |  | FUN292914 | Oxidative stress | Protection from Reactive Oxygen Species | 116 | 0.0055 |
|  |  | FUN293074 | Oxidative stress | Protection from Reactive Oxygen Species | 116 | 0.0051 |
|  |  | FUN293653 | Oxidative stress | Rubrerythrin | 115 | 0.0052 |
|  |  | FUN293823 | Periplasmic Stress | Periplasmic Stress Response | 116 | 0.0053 |
|  | High aridity~ | FUN281915 | - | Dimethylarginine metabolism | 23 | 0.0027 |
|  |  | FUN283344 | - | Hfl operon | 21 | 0.0027 |
|  |  | FUN283514 | - | SigmaB stress responce regulation | 20 | 0.0027 |
|  |  | FUN283735 | - | SigmaB stress responce regulation | 20 | 0.0027 |
|  |  | FUN285495 | Detoxification | Nudix proteins (nucleoside triphosphate hydrolases) | 23 | 0.0027 |
|  |  | FUN286134 | Detoxification | Uptake of selenate and selenite | 22 | 0.0026 |
|  |  | FUN286204 | Detoxification | Uptake of selenate and selenite | 20 | 0.0027 |
|  |  | FUN286614 | Heat shock | At5g63290 | 20 | 0.0029 |
|  |  | FUN286694 | Heat shock | At5g63290 | 21 | 0.0027 |
|  |  | FUN287143 | Heat shock | Heat shock dnaK gene cluster extended | 25 | 0.0027 |
|  |  | FUN287255 | Heat shock | Heat shock dnaK gene cluster extended | 27 | 0.003 |
|  |  | FUN287295 | Heat shock | Heat shock dnaK gene cluster extended | 24 | 0.0027 |
|  |  | FUN287715 | Osmotic stress | Choline and Betaine Uptake and Betaine Biosynthesis | 20 | 0.0027 |
|  |  | FUN287735 | Osmotic stress | Choline and Betaine Uptake and Betaine Biosynthesis | 21 | 0.0029 |
|  |  | FUN287903 | Osmotic stress | Choline and Betaine Uptake and Betaine Biosynthesis | 20 | 0.0026 |
|  |  | FUN287965 | Osmotic stress | Choline and Betaine Uptake and Betaine Biosynthesis | 22 | 0.0025 |
|  |  | FUN288613 | Osmotic stress | Gycosylglycerates | 22 | 0.0026 |
|  |  | FUN289913 | Oxidative stress | Glutathione: Biosynthesis and gamma-glutamyl cycle | 22 | 0.0027 |
|  |  | FUN289975 | Oxidative stress | Glutathione: Biosynthesis and gamma-glutamyl cycle | 22 | 0.0026 |
|  |  | FUN289993 | Oxidative stress | Glutathione: Biosynthesis and gamma-glutamyl cycle | 25 | 0.0027 |
|  |  | FUN290272 | Oxidative stress | Glutathione: Non-redox reactions | 25 | 0.0027 |
|  |  | FUN290565 | Oxidative stress | Glutathione: Non-redox reactions | 24 | 0.0028 |
|  |  | FUN290615 | Oxidative stress | Glutathione: Non-redox reactions | 20 | 0.0026 |
|  |  | FUN291004 | Oxidative stress | Glutathione: Redox cycle | 23 | 0.0026 |
|  |  | FUN291324 | Oxidative stress | Oxidative stress | 21 | 0.0026 |
|  |  | FUN291425 | Oxidative stress | Oxidative stress | 22 | 0.0028 |
|  |  | FUN291914 | Oxidative stress | Oxidative stress | 20 | 0.0025 |
|  |  | FUN292275 | Oxidative stress | Oxidative stress | 20 | 0.0028 |
|  |  | FUN293805 | Periplasmic Stress | Periplasmic Stress Response | 23 | 0.0025 |
|  |  | FUN293905 | Periplasmic Stress | Periplasmic Stress Response | 21 | 0.0025 |
| Endosphere | Low aridity~ | FUN281705 | - | Carbon Starvation | 63 | 0.0054 |
|  |  | FUN281774 | - | Carbon Starvation | 55 | 0.0054 |
|  |  | FUN281916 | - | Dimethylarginine metabolism | 58 | 0.0059 |
|  |  | FUN282454 | - | Flavohaemoglobin | 47 | 0.0055 |
|  |  | FUN283375 | - | Hfl operon | 67 | 0.0057 |
|  |  | FUN283723 | - | SigmaB stress responce regulation | 52 | 0.0057 |
|  |  | FUN284056 | - | Universal stress protein family | 55 | 0.0051 |
|  |  | FUN284196 | Cold shock | Cold shock, CspA family of proteins | 45 | 0.0053 |
|  |  | FUN284806 | Detoxification | Housecleaning nucleoside triphosphate pyrophosphatases | 44 | 0.0056 |
|  |  | FUN286205 | Detoxification | Uptake of selenate and selenite | 47 | 0.0054 |
|  |  | FUN286326 | Heat shock | At5g63290 | 50 | 0.0053 |
|  |  | FUN286555 | Heat shock | At5g63290 | 53 | 0.0058 |
|  |  | FUN287236 | Heat shock | Heat shock dnaK gene cluster extended | 53 | 0.0057 |
|  |  | FUN287365 | Heat shock | Heat shock dnaK gene cluster extended | 62 | 0.0056 |
|  |  | FUN287736 | Osmotic stress | Choline and Betaine Uptake and Betaine Biosynthesis | 49 | 0.0053 |
|  |  | FUN287864 | Osmotic stress | Choline and Betaine Uptake and Betaine Biosynthesis | 47 | 0.0052 |
|  |  | FUN287966 | Osmotic stress | Choline and Betaine Uptake and Betaine Biosynthesis | 43 | 0.0055 |
|  |  | FUN287995 | Osmotic stress | Choline and Betaine Uptake and Betaine Biosynthesis | 47 | 0.0052 |
|  |  | FUN288184 | Osmotic stress | Choline and Betaine Uptake and Betaine Biosynthesis | 51 | 0.0059 |
|  |  | FUN288554 | Osmotic stress | Gycosylglycerates | 46 | 0.005 |
|  |  | FUN288614 | Osmotic stress | Gycosylglycerates | 57 | 0.0055 |
|  |  | FUN289225 | Oxidative stress | Glutaredoxins | 59 | 0.0054 |
|  |  | FUN289346 | Oxidative stress | Glutaredoxins | 44 | 0.0053 |
|  |  | FUN290306 | Oxidative stress | Glutathione: Non-redox reactions | 67 | 0.0059 |
|  |  | FUN290425 | Oxidative stress | Glutathione: Non-redox reactions | 51 | 0.0057 |
|  |  | FUN290616 | Oxidative stress | Glutathione: Non-redox reactions | 48 | 0.0056 |
|  |  | FUN291685 | Oxidative stress | Oxidative stress | 67 | 0.0055 |
|  |  | FUN291915 | Oxidative stress | Oxidative stress | 53 | 0.0057 |
|  |  | FUN292266 | Oxidative stress | Oxidative stress | 67 | 0.0057 |
|  |  | FUN293225 | Oxidative stress | Redox-dependent regulation of nucleus processes | 48 | 0.0057 |
|  | Medium aridity~ | FUN281605 | - | Bacterial hemoglobins | 119 | 0.0052 |
|  |  | FUN281706 | - | Carbon Starvation | 116 | 0.0054 |
|  |  | FUN281775 | - | Carbon Starvation | 117 | 0.0054 |
|  |  | FUN281917 | - | Dimethylarginine metabolism | 117 | 0.0052 |
|  |  | FUN282726 | - | Flavohaemoglobin | 115 | 0.0053 |
|  |  | FUN284647 | Detoxification | Housecleaning nucleoside triphosphate pyrophosphatases | 124 | 0.0053 |
|  |  | FUN284676 | Detoxification | Housecleaning nucleoside triphosphate pyrophosphatases | 121 | 0.0054 |
|  |  | FUN285573 | Detoxification | Nudix proteins (nucleoside triphosphate hydrolases) | 115 | 0.0051 |
|  |  | FUN286307 | Heat shock | At5g63290 | 120 | 0.0053 |
|  |  | FUN286365 | Heat shock | At5g63290 | 120 | 0.0051 |
|  |  | FUN286867 | Heat shock | Heat shock dnaK gene cluster extended | 118 | 0.0051 |
|  |  | FUN287027 | Heat shock | Heat shock dnaK gene cluster extended | 115 | 0.0054 |
|  |  | FUN287077 | Heat shock | Heat shock dnaK gene cluster extended | 115 | 0.0053 |
|  |  | FUN287615 | Osmotic stress | Choline and Betaine Uptake and Betaine Biosynthesis | 115 | 0.0055 |
|  |  | FUN288887 | Osmotic stress | Synthesis of osmoregulated periplasmic glucans | 124 | 0.0054 |
|  |  | FUN289977 | Oxidative stress | Glutathione: Biosynthesis and gamma-glutamyl cycle | 118 | 0.0054 |
|  |  | FUN289995 | Oxidative stress | Glutathione: Biosynthesis and gamma-glutamyl cycle | 115 | 0.0052 |
|  |  | FUN290127 | Oxidative stress | Glutathione: Biosynthesis and gamma-glutamyl cycle | 116 | 0.005 |
|  |  | FUN290274 | Oxidative stress | Glutathione: Non-redox reactions | 117 | 0.0056 |
|  |  | FUN290987 | Oxidative stress | Glutathione: Redox cycle | 120 | 0.0052 |
|  |  | FUN291346 | Oxidative stress | Oxidative stress | 117 | 0.0054 |
|  |  | FUN291837 | Oxidative stress | Oxidative stress | 122 | 0.0051 |
|  |  | FUN291955 | Oxidative stress | Oxidative stress | 115 | 0.0053 |
|  |  | FUN292047 | Oxidative stress | Oxidative stress | 118 | 0.0053 |
|  |  | FUN292267 | Oxidative stress | Oxidative stress | 117 | 0.0052 |
|  |  | FUN292776 | Oxidative stress | Protection from Reactive Oxygen Species | 116 | 0.0053 |
|  |  | FUN292917 | Oxidative stress | Protection from Reactive Oxygen Species | 116 | 0.0055 |
|  |  | FUN293077 | Oxidative stress | Protection from Reactive Oxygen Species | 116 | 0.0051 |
|  |  | FUN293655 | Oxidative stress | Rubrerythrin | 115 | 0.0052 |
|  |  | FUN293824 | Periplasmic Stress | Periplasmic Stress Response | 116 | 0.0053 |
|  | High aridity~ | FUN281918 | - | Dimethylarginine metabolism | 23 | 0.0027 |
|  |  | FUN283347 | - | Hfl operon | 21 | 0.0027 |
|  |  | FUN283517 | - | SigmaB stress responce regulation | 20 | 0.0027 |
|  |  | FUN283738 | - | SigmaB stress responce regulation | 20 | 0.0027 |
|  |  | FUN285498 | Detoxification | Nudix proteins (nucleoside triphosphate hydrolases) | 23 | 0.0027 |
|  |  | FUN286137 | Detoxification | Uptake of selenate and selenite | 22 | 0.0026 |
|  |  | FUN286207 | Detoxification | Uptake of selenate and selenite | 20 | 0.0027 |
|  |  | FUN286617 | Heat shock | At5g63290 | 20 | 0.0029 |
|  |  | FUN286696 | Heat shock | At5g63290 | 21 | 0.0027 |
|  |  | FUN287146 | Heat shock | Heat shock dnaK gene cluster extended | 25 | 0.0027 |
|  |  | FUN287258 | Heat shock | Heat shock dnaK gene cluster extended | 27 | 0.003 |
|  |  | FUN287298 | Heat shock | Heat shock dnaK gene cluster extended | 24 | 0.0027 |
|  |  | FUN287718 | Osmotic stress | Choline and Betaine Uptake and Betaine Biosynthesis | 20 | 0.0027 |
|  |  | FUN287738 | Osmotic stress | Choline and Betaine Uptake and Betaine Biosynthesis | 21 | 0.0029 |
|  |  | FUN287905 | Osmotic stress | Choline and Betaine Uptake and Betaine Biosynthesis | 20 | 0.0026 |
|  |  | FUN287968 | Osmotic stress | Choline and Betaine Uptake and Betaine Biosynthesis | 22 | 0.0025 |
|  |  | FUN288615 | Osmotic stress | Gycosylglycerates | 22 | 0.0026 |
|  |  | FUN289916 | Oxidative stress | Glutathione: Biosynthesis and gamma-glutamyl cycle | 22 | 0.0027 |
|  |  | FUN289978 | Oxidative stress | Glutathione: Biosynthesis and gamma-glutamyl cycle | 22 | 0.0026 |
|  |  | FUN289996 | Oxidative stress | Glutathione: Biosynthesis and gamma-glutamyl cycle | 25 | 0.0027 |
|  |  | FUN290275 | Oxidative stress | Glutathione: Non-redox reactions | 25 | 0.0027 |
|  |  | FUN290568 | Oxidative stress | Glutathione: Non-redox reactions | 24 | 0.0028 |
|  |  | FUN290618 | Oxidative stress | Glutathione: Non-redox reactions | 20 | 0.0026 |
|  |  | FUN291007 | Oxidative stress | Glutathione: Redox cycle | 23 | 0.0026 |
|  |  | FUN291327 | Oxidative stress | Oxidative stress | 21 | 0.0026 |
|  |  | FUN291428 | Oxidative stress | Oxidative stress | 22 | 0.0028 |
|  |  | FUN291917 | Oxidative stress | Oxidative stress | 20 | 0.0025 |
|  |  | FUN292278 | Oxidative stress | Oxidative stress | 20 | 0.0028 |
|  |  | FUN293808 | Periplasmic Stress | Periplasmic Stress Response | 23 | 0.0025 |
|  |  | FUN293908 | Periplasmic Stress | Periplasmic Stress Response | 21 | 0.0025 |

**REFERENCES**

ALA 2014. Mean annual aridity index. Atlas of Living Australia. https://spatial.ala.org.au/layers. https://researchdata.edu.au/mean-annual-aridity-index/340830 [Accessed 21 Dec. 2021].

BELBIN, L. The Atlas of Livings Australia’s Spatial Portal. In: JONES, M. & GRIES, B., eds. In, Proceedings of the Environmental Information Management Conference 2011 (EIM 2011), 2011.

BLAIR, G., CHINOIM, N., LEFROY, R., ANDERSON, G., & CROCKER, G. 1991. A soil sulfur test for pastures and crops. Soil Research 29:619-626.

BONHAM, C. D. 2013. Measurements for terrestrial vegetation. 2nd edition. Wiley-Blackwell, West Sussex, England.

COLWELL, J. D. 1965. An automatic procedure for the determination of Phosphorus in sodium hydrogen carbonate extracts of soils. Chemistry Industry 22:893-895.

COMMONWEALTH OF AUSTRALIA, 2009. Elevation (metres above mean sea level), 0.01 degree (~1 km) resolution. Bureau of Rural Sciences. Derived from 9-second DEM v3. Available at: http://www.daff.gov.au/abares/data/mcass [Accessed 27 Aug. 2025].

CSIRO Ecosystem Sciences, 2010a. Precipitation – annual (Bio12), 0.01° (~1 km) gridded dataset. Derived using ANUCLIM v6 (beta) with 1990-centred climate surfaces by Dr Kristen Williams. Reference year: 2008. Available at: http://fennerschool.anu.edu.au/publications/software/ [Accessed 27 Aug. 2025].

CSIRO Ecosystem Sciences, 2010b. Temperature – annual mean (Bio01), 0.01° (~1 km) gridded dataset. Derived using ANUCLIM v6 (beta) with 1990-centred climate surfaces by Dr Kristen Williams. Reference year: 2008. Available at: http://fennerschool.anu.edu.au/publications/software/ [Accessed 27 Aug. 2025].

GBIF.org. 2023. Occurrence Download. The Global Biodiversity Information Facility.

HIJMANS, R., 2023. terra: Spatial Data Analysis.

MCPHERSON, M. R., WANG, P., MARSH, E. L., MITCHELL, R. B., and SCHACHTMAN, D. P. 2018. Isolation and analysis of microbial communities in soil, rhizosphere, and roots in perennial grass experiments. JoVE:e57932.

WALKLEY, A. & ARMSTRONG, B. I. 1934. An examination of the Degtjareff method for determining soil organic matter, and a proposed modification of the chromic acid titration method. Soil Science 37:29-38.

ZOMER, R. J., Xu, J. & TRABUCCO, A. 2022. Version 3 of the Global Aridity Index and Potential Evapotranspiration Database. Scientific Data 9:409.
